# Molecular Basis for Inhibition of Heparanases and β-Glucuronidases by Siastatin B

**DOI:** 10.1021/jacs.3c04162

**Published:** 2023-12-20

**Authors:** Yurong Chen, Adrianus M. C. H. van den Nieuwendijk, Liang Wu, Elisha Moran, Foteini Skoulikopoulou, Vera van Riet, Hermen S. Overkleeft, Gideon J. Davies, Zachary Armstrong

**Affiliations:** † Leiden Institute of Chemistry, Leiden University, Einsteinweg 55, 2300 RA Leiden, The Netherlands; ‡ York Structural Biology Laboratory, Department of Chemistry, The University of York, YO10 5DD York, U.K.

## Abstract

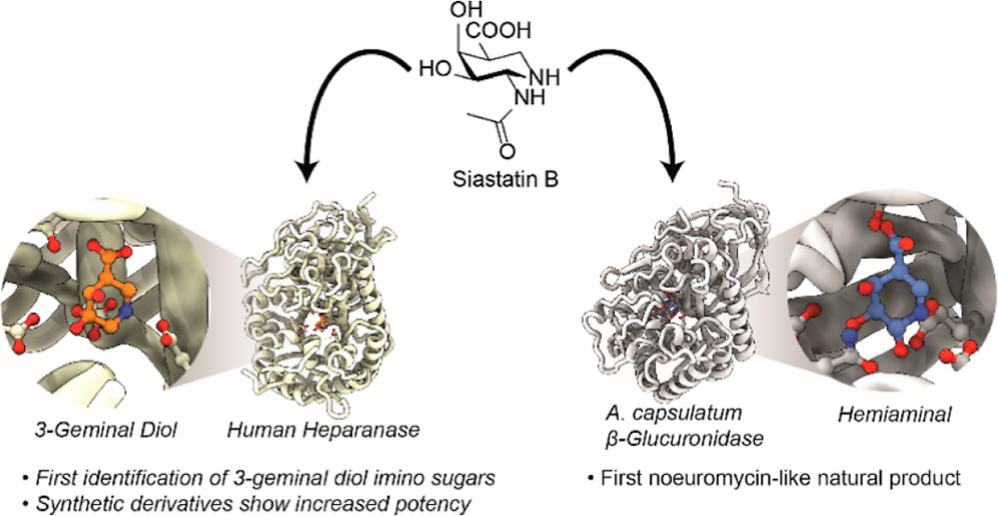

Siastatin B is a potent and effective iminosugar inhibitor of three diverse glycosidase classes, namely, sialidases, β-*d*-glucuronidases, and *N*-acetyl-glucosaminidases. The mode of inhibition of glucuronidases, in contrast to sialidases, has long been enigmatic as siastatin B appears too bulky and incorrectly substituted to be accommodated within a β-*d*-glucuronidase active site pocket. Herein, we show through crystallographic analysis of protein-inhibitor complexes that siastatin B generates both a hemiaminal and a 3-geminal diol iminosugar (3-GDI) that are, rather than the parent compound, directly responsible for enzyme inhibition. The hemiaminal product is the first observation of a natural product that belongs to the noeuromycin class of inhibitors. Additionally, the 3-GDI represents a new and potent class of the iminosugar glycosidase inhibitor. To substantiate our findings, we synthesized both the *gluco*- and *galacto*-configured 3-GDIs and characterized their binding both structurally and kinetically to exo-β-*d*-glucuronidases and the anticancer target human heparanase. This revealed submicromolar inhibition of exo-β-*d*-glucuronidases and an unprecedented binding mode by this new class of inhibitor. Our results reveal the mechanism by which siastatin B acts as a broad-spectrum glycosidase inhibitor, identify a new class of glycosidase inhibitor, and suggest new functionalities that can be incorporated into future generations of glycosidase inhibitors.

## Introduction

Iminosugars are carbohydrate mimetics that contain an endocyclic nitrogen in place of an endocyclic oxygen. This class of compounds can be both potent and selective inhibitors of carbohydrate processing enzymes.^1−3^ As a result of their potency, the iminosugars migalastat and miglustat are used clinically to treat lysosomal storage disorders^4,5^ while miglitol are used to treat diabetes.^6^ Furthermore, iminosugars have been investigated as potential therapeutics for tumor metastasis,^7,8^ cystic fibrosis,^9^ and as antiviral therapeutics.^10,11^

**Figure 1:**
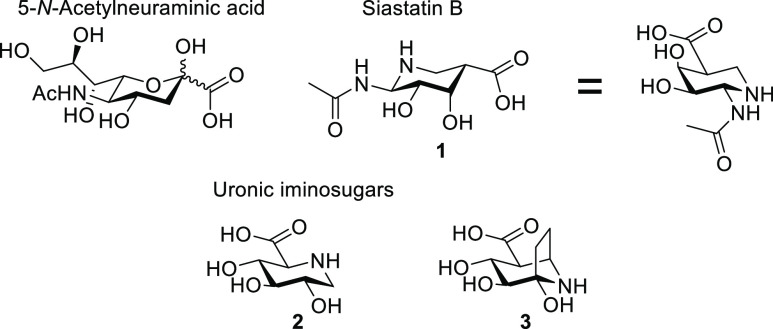
Structures of 5-*N*-acetylneuraminic acid, siastatin B (**1**), and the uronic acid-configured iminosugars uronic-deoxynojirimycin (**2**) and uronic-noeurostegine (**3**).

Siastatin B (**1**) is a natural product 1-*N*-iminosugar, first identified in 1974 from a *Streptomyces verticillus* culture by Umezewa and co-workers.^12^ This iminosugar is a geminal diamine, in which the anomeric carbon is replaced by a nitrogen, and the 2-position has an *N*-acetyl functional group (see Figure 1). Siastatin B effectively inhibits a wide range of sialidases from viral,^13^ bacterial,^14^ and human origin^15^ with micromolar potency as it structurally resembles 5*N*-acetylneuraminic acid. Surprisingly, siastatin B also inhibits both β-glucuronidases and human heparanase,^12,16^ which cleave β-glucuronic acid residues but not sialic acid or *N*-acetylated sugars. Although siastatin B resembles uronic acid-configured iminosugars [such as the β-glucuronidase inhibitors uronic-deoxynojirimycin (**2**),^17,18^ uronic-noeurostegine (**3**),^19^ and uronic-isofagamine^20,21^], it bears two major modifications that one would expect to interfere with β-glucuronidase activity. The first of these is the presence of the acetamido group at the 2-position. Glycosidases are typically extremely specific for the substituents at the 2-position of their substrates^22−24^ as interactions with these substituents enable the stabilization of a planar C5–O–C1–C2 oxocarbenium ion transition state.^25,26^ Furthermore, investigation of the active site of human heparanase in complex with heparan oligomers^27,28^ and inhibitors^29^ reveals that there is little space within the active site to accommodate an *N*-acetyl group at the 2-position. The second stereochemical problem that may interfere with the binding of siastatin B to β-glucuronidases is the “*galacto*” configuration of the 4-position. In siastatin B, this hydroxyl is axial (in the ^4^C_1_ conformation), while in the natural substrates of these enzymes–glucuronides–this position is “*gluco*”, that is, equatorial. Together, these modifications raise the possibility that the inhibition of β-glucuronidases by siastatin B may occur in an unanticipated fashion.

One hint toward the inhibition caused by siastatin B comes from the synthesis of a series of siastatin analogues reported by Nishimura and co-authors that were modified with a trifluoroacetamido substituent at the 2-position.^30,31^ These 2-trifluoroacetamido are potent inhibitors of bovine liver β-*d*-glucuronidase^30,31^ and micromolar inhibitors of recombinant human heparanase (HPSE).^31^ NMR analyses of 2-trifluoroacetamido siastatin in the media of enzyme assays have suggested that it can undergo pH-dependent decomposition and rearrangement in solution, yielding compounds that could act as the true enzyme inhibitors.^32^ However, this solvent-mediated rearrangement has not been demonstrated for siastatin B, which has always been regarded as stable in aqueous solution.^33^

Our interest in heparanase inhibitors^29,34^ has led us to re-examine the inhibition of heparanases and β-glucuronidases by siastatin B. Human heparanase (HPSE) is an *endo*-β-glucuronidase that catalyzes the cleavage of heparan sulfate (HS). Overexpression of HPSE strongly drives the growth of aggressive metastatic cancers^35^ and leads to excessive HS degradation within the extracellular matrix, thereby facilitating cancer cell migration,^36,37^ while growth factors and cytokines liberated upon HS degradation stimulate proliferation and angiogenesis.^38,39^ Inhibitors of HPSE are potential anticancer therapeutics and have shown antimetastatic activity in animal models.^29,40,41^ It is our hope that by understanding how glycosidases are inhibited by siastatin B, we can identify and design potent inhibitors for this enzyme. Here, we used structural biology to investigate the mode of binding of siastatin B to β-glucuronidases. This unexpectedly revealed that contaminating breakdown products are instead responsible for the inhibition of β-glucuronidases. In particular, we identify both the first natural product noeuromycin-type inhibitor and a new class of potent glycosidase inhibitor–the 3-geminal-diol iminosugars (3-GDIs). To substantiate our findings, we have synthesized both the *gluco*- and *galacto*-configured 3-GDIs and characterized their binding to HPSE and β-glucuronidases through detailed enzyme kinetics, competitive activity-based protein profiling, and structural biology.

## Results and Discussion

To determine how siastatin B inhibits β-glucuronidases, we first sought to determine whether inhibition was due to the presence of contaminants from either the commercial material or degradation products resulting from prolonged incubation in an aqueous solution or in the presence of a β-glucuronidase. NMR analysis of commercial siastatin B showed no contaminants present, and no contaminating peaks appeared after incubation at pH 5.0 for 18 h, see Figure S1. We next determined whether siastatin B degradation could be caused by prolonged incubation with a β-glucuronidase. ^1^H NMR analysis of siastatin B incubation with recombinant human heparanase (present at concentration of 1.2 μM) showed very minor additional peaks in the 3.2–3.0 range, with no loss in the starting peaks observed.

**Figure 2:**
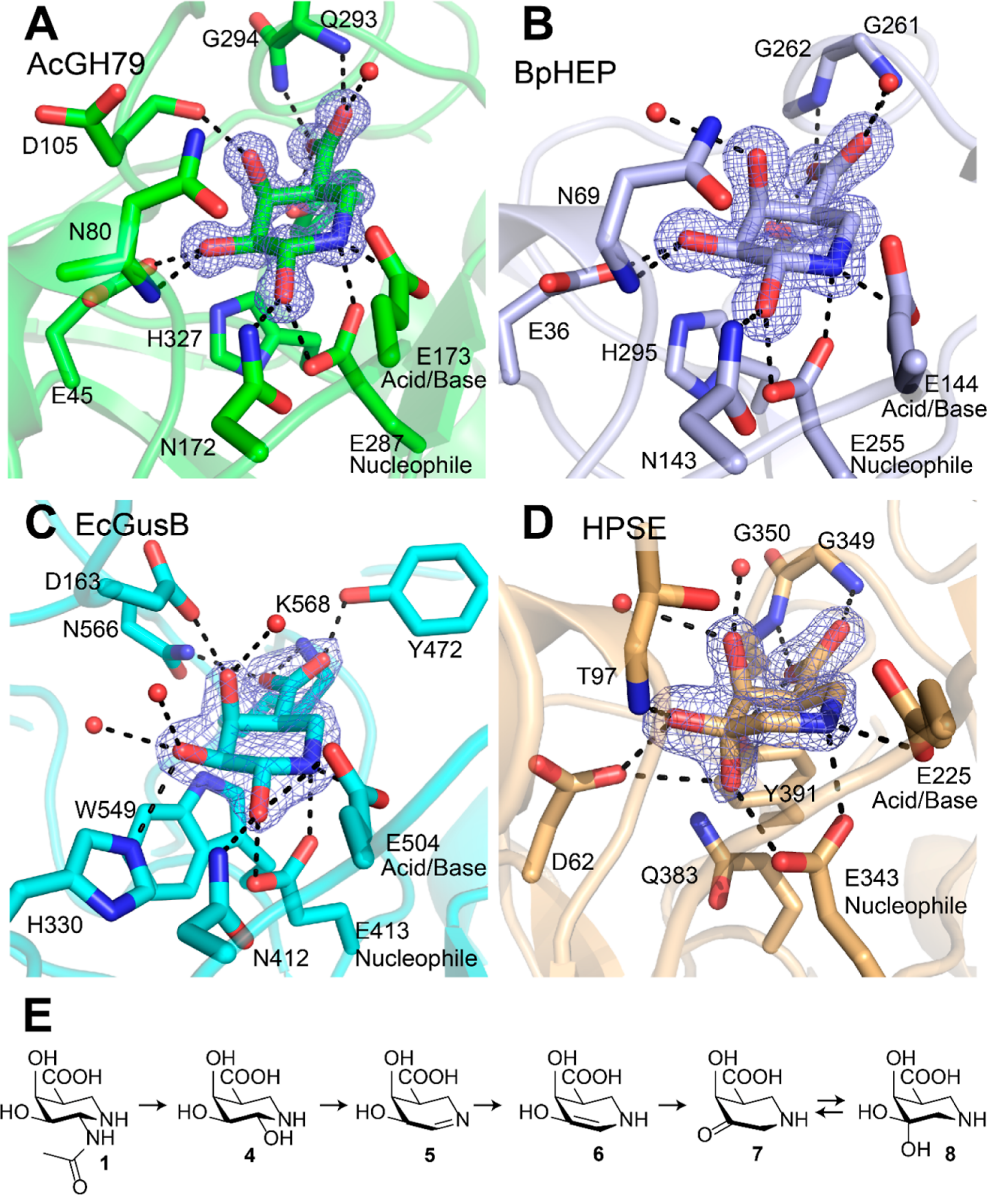
Structures of heparanases and β-glucuronidases soaked with siastatin B and the proposed breakdown mechanism. Siastatin breakdown products are shown to bound to (A) AcGH79, (B) BpHEP, (C) EcGusB, and (D) HPSE. Electron density (2*F*_o_ – *F*_c_) is shown for the ligand as a blue mesh contoured at 2 σ (AcGH79 = 0.92 e^–^/Å^3^, BpHep = 0.77 e^–^/Å^3^, EcGusB = 0.41 e^–^/Å^3^, and HPSE = 0.61 e^–^/Å^3^). The polypeptide is shown in the cartoon form, with active site residues shown as sticks. Apparent hydrogen bonding interactions are shown as dotted black lines. (E) Proposed degradation of the siastatin B **1** into galacturonic-noeuromycin **4** and the 3-geminial diol iminosugar **8.**

We next performed high-resolution mass spectrometry to determine whether there were any minor contaminants present in our siastatin B sample (Figure S2). In addition to an *m*/*z* corresponding to siastatin B (219 in the positive-ion mode, 217 in the negative-ion mode), we also observed a peak at *m*/*z* = 160 (in the positive-ion mode, and 158 in the negative-ion mode) that could belong to the siastatin break down products **5**, **6**, or **7** (Figure 2). Additionally, we observed a peak at *m*/*z* = 178 in the positive-ion mode that could belong to a noeuromycin like breakdown product (**4**) or a 3-geminal-diol (**8**). These minor species could be separated and identified by LC–MS (Figure S2). We next purified the major peak using preparative HPLC–MS, although we were unable to completely remove all contaminants (see Figure S3), the resulting material had substantially reduced concentrations of the *m*/*z* = 158 and *m*/*z* = 176 peaks. This HPLC-purified material was used in inhibition assays of *E. coli* β-glucuronidase (EcGusB) and *Acidobacterium capsulatum* β-glucuronidase (AcGH79), see Figure S3. Both enzymes were inhibited less by the purified material, indicating that siastatin B contaminants are indeed inhibitors of β-glucuronidases. As the contaminating substances could be one of several isomers, uncertainty in the active species remained.

### Structural Basis for Siastatin B Inhibition of Heparanases and β-Glucuronidases

To determine how siastatin B inhibits both heparanases and β-glucuronidases, we determined crystal structures of co-complexes between siastatin B and each of AcGH79, EcGusB, human heparanase (HPSE), and *Burkholderia pseudomallei* heparanase (BpHEP), see Table S1 for data collection and refinement statistics. As we showed by NMR that siastatin B is stable under enzymatic assay conditions, we expected to observe it present in the active site of the enzymes. However, we were surprised to find that although each of the structures contains a ligand present with full occupancy in the active site, none of the observed ligands are siastatin B (Figure 2). Instead, siastatin B breakdown products are present in all structures.

Remarkably, not all active sites contained the same inhibitor. Three of the four enzymes (AcGH79, BpHEP, and EcGusB) contained “galacturonic-noeuromycin” (**4**) bound in the −1 position of the active site, as opposed to siastatin B (Figure 2). We confirmed the presence of an alcohol at the 2-position–as opposed to an amine, which would result from deacetylation–through examination of the *B*-factors within the high-resolution crystal structures of AcGH79 and BpHEP, see Figure S4. When the 2-position is modeled as an alcohol, the *B*-factor of the 2-substituent is consistent with the 2-carbon. However, when the 2-position is modeled as an amine, the *B*-factor of this functional group is depressed, which is anticipated for a nitrogen being accommodated within the electron density of an oxygen.

We expect that the *galacturonic*-noeuromycin (**4**), observed in these active sites is formed through the elimination of the *N*-acetyl group of siastatin B, resulting in imine **5** that is subsequently hydrated to form hemiaminal **4**, see Figure 2E. The smaller functional group at the 2-position is both well accommodated within the active sites of these enzymes and the correct functional group (OH instead of NH-acetyl). The 2-hydroxyl is within hydrogen bonding distance of the catalytic nucleophile and an active site asparagine in all three of these structures, thereby mimicking the interactions seen for natural substrates^28^ and other inhibitors.^20,29^ The 4-hydroxyl of **4** has *galacto*-stereochemistry in contrast to the *gluco*-configuration of the natural enzyme substrate; however, this position also appears to have productive binding to the active site. The 4-hydroxyl of **4** forms hydrogen bond interactions with the enzyme active site, for both AcGH79 and EcGusB, see Figure 2AC. The 4-hydroxyl of **4** complexed to BpHEP does not form a hydrogen bond with the amino acids in the enzyme active site; however it forms a hydrogen bond with an active site water, see Figure 2B.

Unlike the three other co-complex structures, the structure of HPSE crystals soaked with siastatin B contained a 3-geminial diol iminosugar (**8**). Compound **8** is a further breakdown product of **4**, formed by the elimination of the 2-hydroxyl to form imine **5** which rearranges to enamine **6**, and the enol can then tautomerize to give ketoamine **7**. This ketoamine can then become hydrated at the 3-position to give the observed geminal diol **8**. A similar breakdown of noeuromycin and fuco-noeuromycin, which are unstable at neutral pH, has been previously reported and also resulted in iminosugars bearing a hydrated ketone.^42^ Despite the additional hydroxyl present at the 3-position of **8**, when compared to glucuronic acid, this compound appears to be well accommodated in the active site of HPSE. Indeed, both hydroxyls present at the 3-position form hydrogen bond interactions with the active site. As well, the hydroxyl on the β-face forms a hydrogen bond with Asp62 and the backbone nitrogen of Thr97, while the hydroxyl on the α-face is positioned to form hydrogen bonds with the catalytic nucleophile Glu343 and Asp62. The hydroxyl at the 4-position of inhibitor **3** lacks the hydrogen bond with Trp391 which is seen for *gluco*-configured inhibitors.^20,29^ However, as in the costructure of **4** and BpHEP, the axial 4-hydroxyl forms a hydrogen bond with water present in the crystal structure.

### Synthesis of Siastatin B Breakdown Products

As 3-GDI inhibitors represent a new class of glycosidase inhibitors, we sought to chemically synthesize them to better understand their binding and potency. We synthesized the geminal diol siastatin B breakdown product observed in cocrystal structure with HPSE (**8**) and its *gluco*-configured 4-epimer **9**, to probe whether this compound would be more potent. We also synthesized both galacturonic acid isofagomine **10** and glucuronic acid isofagomine **11** to gain further insights into the influence of the geminal diol at the 3-position on inhibition.

**Scheme 1:**
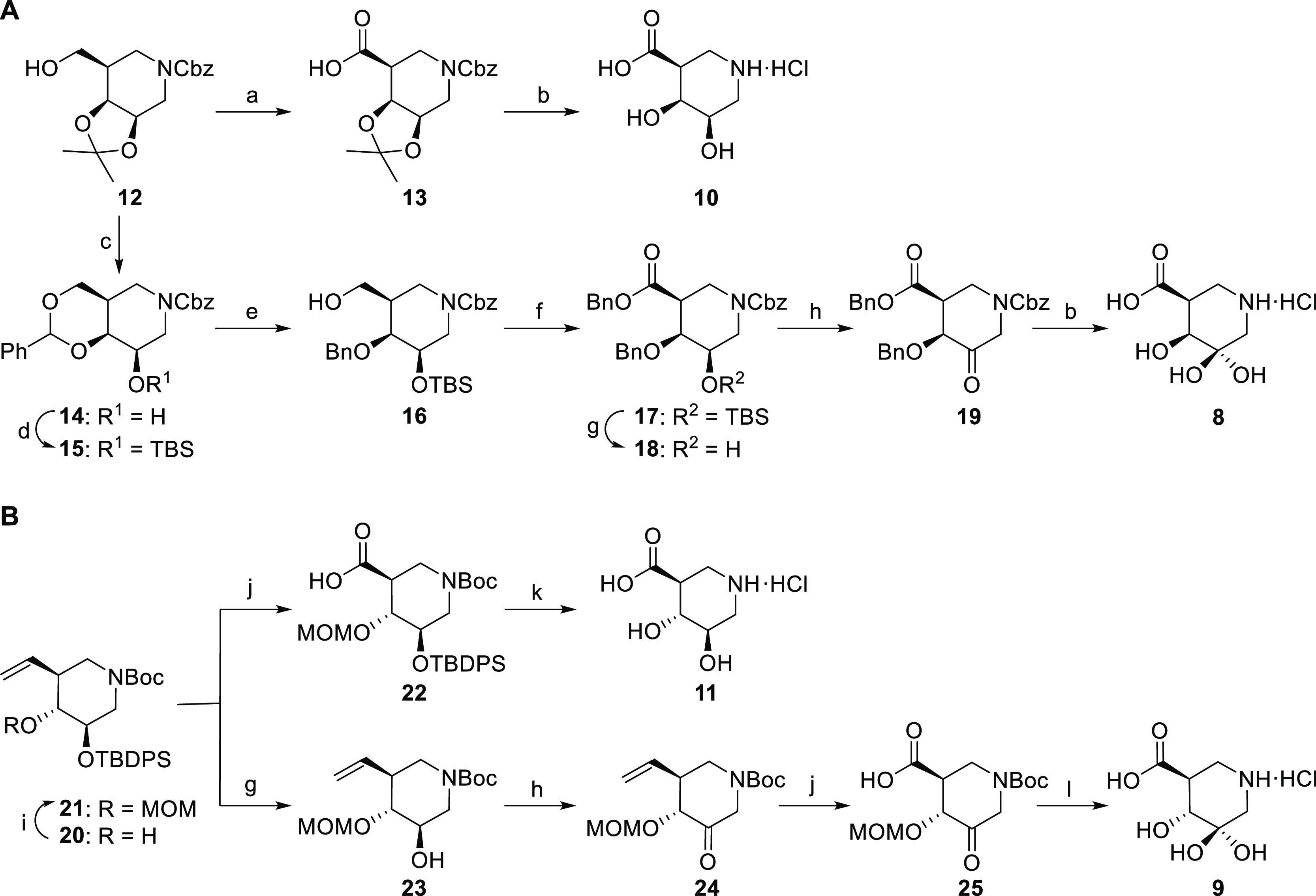
Synthesis of d-Galacturonic Acid-Type 1-*N*-Iminosugars **8** and **10** (A) and d-Glucuronic Acid-Type 1-*N*-Iminosugars **9** and **11** (B). Reagents and Conditions: (a) (i) Dess–Martin Periodinane, DCM, rt; (ii) NaClO_2_, NaH_2_PO_4_, 30% H_2_O_2_, CH_3_CN, H_2_O, 0 °C to rt, 65% over Two Steps; (b) H_2_, 10% Pd/C, H_3_O^+^, THF, rt, **10** Quant, **8** Quant; (c) (i) 8 M HCl, MeOH, rt; (ii) PhCH(OMe)_2_, CSA, DMF, 60 °C, 72% Over Two Steps; (d) TBSCl, Imidazole, DCM, rt, 94%; (e) THF·BH_3_, TMSOTf, DCM, 0 °C to rt, 97%; (f) (i) Jones Reagent, Acetone, rt; (ii) Benzyl Alcohol, DIC, DMAP, DCM, rt, 46% Over Two Steps; (g) TBAF, THF, **18** 0 °C, 94%, **23** rt, 94%; (h) Dess–Martin Periodinane, DCM, rt, **19** 71%, **24** 93%; (i) MOMCl, DIPEA, DCM, Reflux, 87%; (j) RuCl_3_·3H_2_O, NaIO_4_, CCl_4_/CH_3_CN/H_2_O, rt, **22** 64%, **25** 83%; (k) 3 M HCl in H_2_O, dioxane, 100 °C, 95%; and (l) 4 M HCl in dioxane/H_2_O, rt, 70%

Primary alcohol **12** (see Supporting Information and Scheme S1 for its synthesis) was oxidized to carboxylic acid **13** via a two-step oxidation process (Scheme 1A). Deprotection of the isopropylidene acetal and the carboxybenzyl (Cbz) group in **13** was achieved by palladium-catalyzed hydrogenolysis in the presence of acid, affording galacturonic acid isofagomine **10** in the quantitative yield. The synthesis of geminal diol **8** proceeded through the same intermediate (**12**). The 3,4-isopropylidene acetal in **12** was removed under acidic conditions followed by a thermodynamically controlled installation of the benzylidene acetal and subsequent silylation of the remaining 3-hydroxyl group, affording fully protected **15**. Regioselective cleavage of the benzylidene acetal yielded compound **16**, the primary alcohol of which was then oxidized to carboxylic acid followed by benzyl protection to afford benzyl ester **17**. Desilylation of **17** and subsequent oxidation of the resulting secondary alcohol gave ketone **19**, which after global deprotection by catalytic hydrogenation under acidic conditions finally afforded target compound **8** (see the Supporting Information and Scheme S2 for an alternative synthetic route toward **8** via a *tert*-butyl ester intermediate).

The synthesis of *gluco*-configured iminosugars **9** and **11** commenced from common intermediate **20** (see Supporting Information and Scheme S3 for its synthesis), which was treated with methoxymethyl chloride in the presence of excessive base under heating to afford compound **21** (Scheme 1B). Oxidative cleavage of the terminal alkene using ruthenium tetroxide (generated in situ from ruthenium chloride and sodium periodate) gave carboxylic acid **22**. The acid labile protecting groups in **22** were removed by treatment with aqueous 3 M HCl to afford glucuronic acid isofagomine **11** in an excellent yield. All spectroscopic data obtained for **11** proved to be in agreement with those reported in the literature.^43−45^ On the other hand, the silyl group in **21** could be readily removed by tetrabutylammonium fluoride (TBAF) to afford alcohol **23**, which was then oxidized to ketone **24** with Dess–Martin periodinane. Oxidative cleavage of the terminal alkene in **24** followed by global deprotection under acidic conditions finally yielded the target geminal diol **9**.

### 3-GDIs Inhibit Glucuronidases

To assess the potency of the synthesized 1-*N*-iminosugars, we first turned to the exo-acting β-glucuronidases AcGH79 and EcGusB, whose activity can be readily monitored with the use of the fluorogenic substrate 4-methylumbelliferyl glucuronide (MU-GlcUA). As these β-glucuronidases belong to different families–AcGH79 to the GH79 family and EcGusB to the GH2 family^46^—assessing inhibition with these enzymes also gives some insights into the applicability of these molecules. All four synthesized compounds (**8**–**11**) are able to inhibit both enzymes (see Table 1, Figures S5 and S6). The *galacto*-configured 3-GDI **8**—the breakdown product observed in the active site of HPSE—inhibited AcGH79 (*K*_i_ = 5.8 ± 0.5 μM) more potently than did EcGusB (*K*_i_ = 137 ± 3 μM). Comparison of these inhibition constants to those obtained with the *galacturonic*-isofagomine derivative (**10**) reveals that both enzymes are more strongly inhibited by *galacturonic*-isofagomine **10**, with ΔΔ*G* values for *K*_d_ of more than −3 kJ·mol^–1^ for both enzymes.

**Table 1:** Inhibition Constants for the Synthesized Inhibitors

inhibitor	AcGH79 *K*_i_ (μM)	EcGusB *K*_i_ (μM)
**8**	5.8 ± 0.5	137 ± 3
**9**	0.520 ± 0.030	28 ± 1
**10**	1.5 ± 0.4	23 ± 3
**11**	0.022 ± 0.003	1.5 ± 0.3

We also synthesized the *gluco*-configured 1-*N*-iminosugars **9** and **11** in the hopes that by changing the 4-position stereochemistry to that of the natural substrate, we could enhance inhibition. This, indeed, turned out to be the case. The *gluco*-configured 3-GDI (**9**) was significantly more potent than the *galacto*-configured (**8**), with inhibition constants of 520 ± 30 nM and 28 ± 1 μM for AcGH79 and EcGusB, respectively. The *gluco*-configured isofagomine (**11**) proved even more potent, mirroring the trend seen with **10**, resulting in the potent inhibition of AcGH79 and EcGusB that was more than an order of magnitude better than the geminal-diol, see Tables 1 and 2.

**Table 2:** Free Energy Changes Resulting from 4-Epimerization and Conversion of a *gem*-Diol to an Alcohol

inhibitor	AcGH79 ΔΔ*G*, K_*d*_ (kJ mol^–^^1^)	EcGusB ΔΔ*G*, K_*d*_ (kJ mol^–^^1^)
4_*ax*_-OH → 4_eq_-OH		
3-OH	–10.3	–6.7
3-*gem*-diol	–5.9	–3.9
3-*gem*-diol → 3-OH		
4_ax_-OH	–3.3	–4.3
4_eq_-OH	–7.7	–7.1

**Figure 3:**
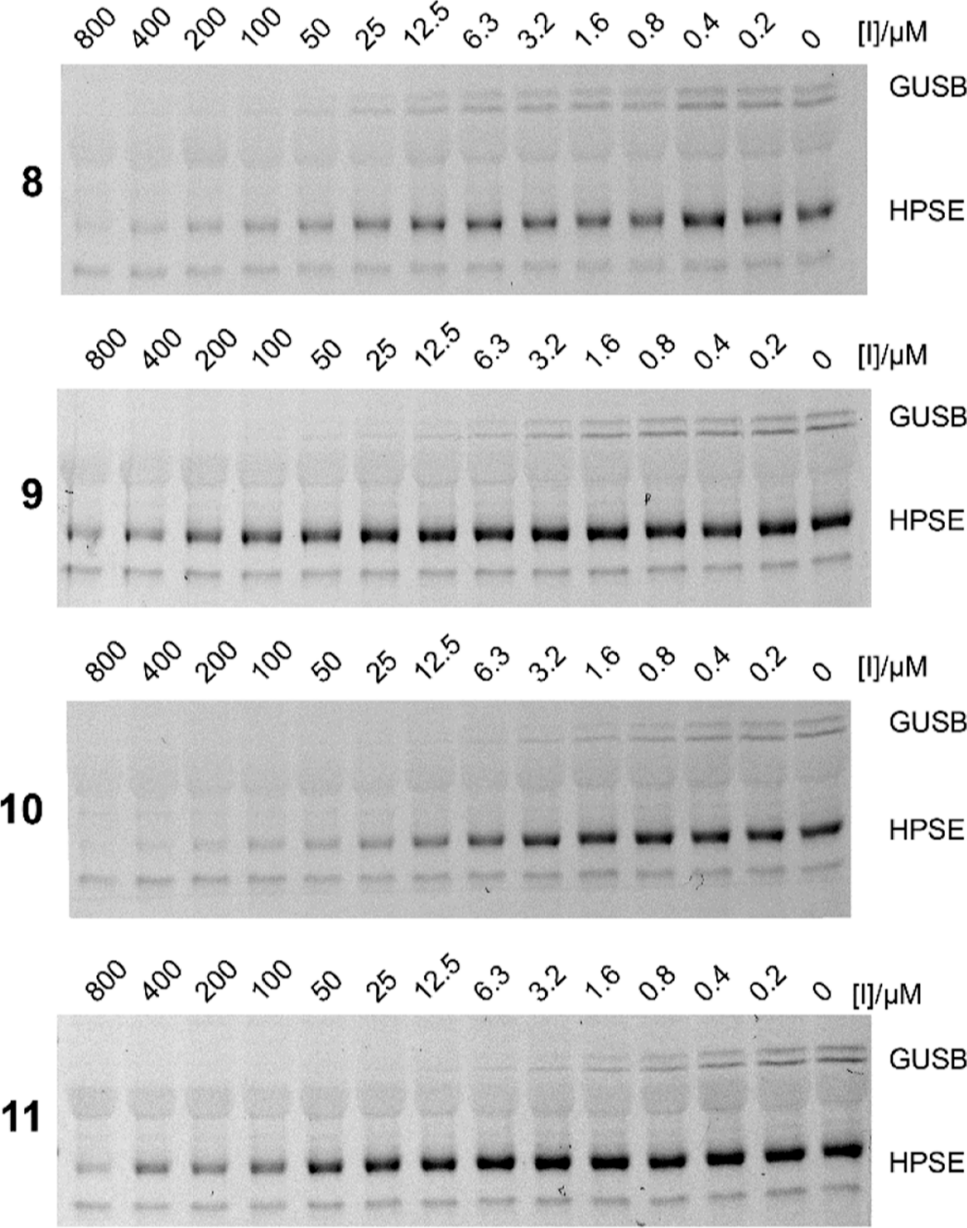
Competitive ABPP of platelet lysate with synthetic iminosugars. Iminosugars **8**–**11** inhibit both GusB and HPSE present in human platelet lysate. Quantitated fluorescence plots used to determine IC_50_ values are available in Figure S7. Full length gel images and replicates are available in Figure S8, and Coomassie stained gels are available in Figure S9.

We next examined whether the synthetic 1-*N*-iminosugars could inhibit HPSE and human β-glucuronidase. To assess the potency of these inhibitors, we used a competitive activity-based protein profiling (cABPP) assay, that measures the ability of the inhibitor to reduce fluorescent labeling of an enzyme by a fluorescent pan-β-glucuronidase activity-based probe (ABP).^16,34^ Using this cABPP assay, we can simultaneously monitor the inhibition of HPSE and GUSB that are both present in platelet lysates.^6,29^ As for the bacterial β-glucuronidases, human GUSB was also effectively inhibited by all the synthesized inhibitors (**8**–**11**), with low or submicromolar IC_50_’s observed, see Table 3 and Figures 3, S7–S9. As observed with the bacterial β-glucuronidases, the *gluco*-configured inhibitors **9** and **11** were more potent inhibitors of human GUSB than their 4-axial *galacto*-analogues. Human heparanase was also inhibited by all four synthetic inhibitors. The IC_50_’s determined for HPSE were greater than that observed for GUSB, which is anticipated for monomer-like inhibitors acting on multisubsite *endo*-acting enzymes. Surprisingly, for HPSE both the *galacto*-configured inhibitors (**8** and **10**) were more potent inhibitors than their *gluco*-configured analogues (**9** and **11**), reversing the trend seen with all of the other β-glucuronidases. However, the trend that inhibition was improved with the removal of the 3-geminal diol was maintained for both GUSB and HPSE. Furthermore, all the synthetic inhibitors were more potent than previously reported for the inhibition of GUSB or HPSE by siastatin B.^47^

**Table 3:** Inhibitory Potency of the Synthesized Inhibitors in the Platelet Lysate

inhibitor	HsGUSB IC_50_ (μM)	HPSE IC_50_ (μM)
**8**	4 ± 2	27 ± 3
**9**	1.7 ± 0.2	200 ± 80
**10**	0.70 ± 0.04	8 ± 1
**11**	0.60 ± 0.06	80 ± 20

### Structural Basis for Inhibition by Synthetic Iminosugars

**Figure 4:**
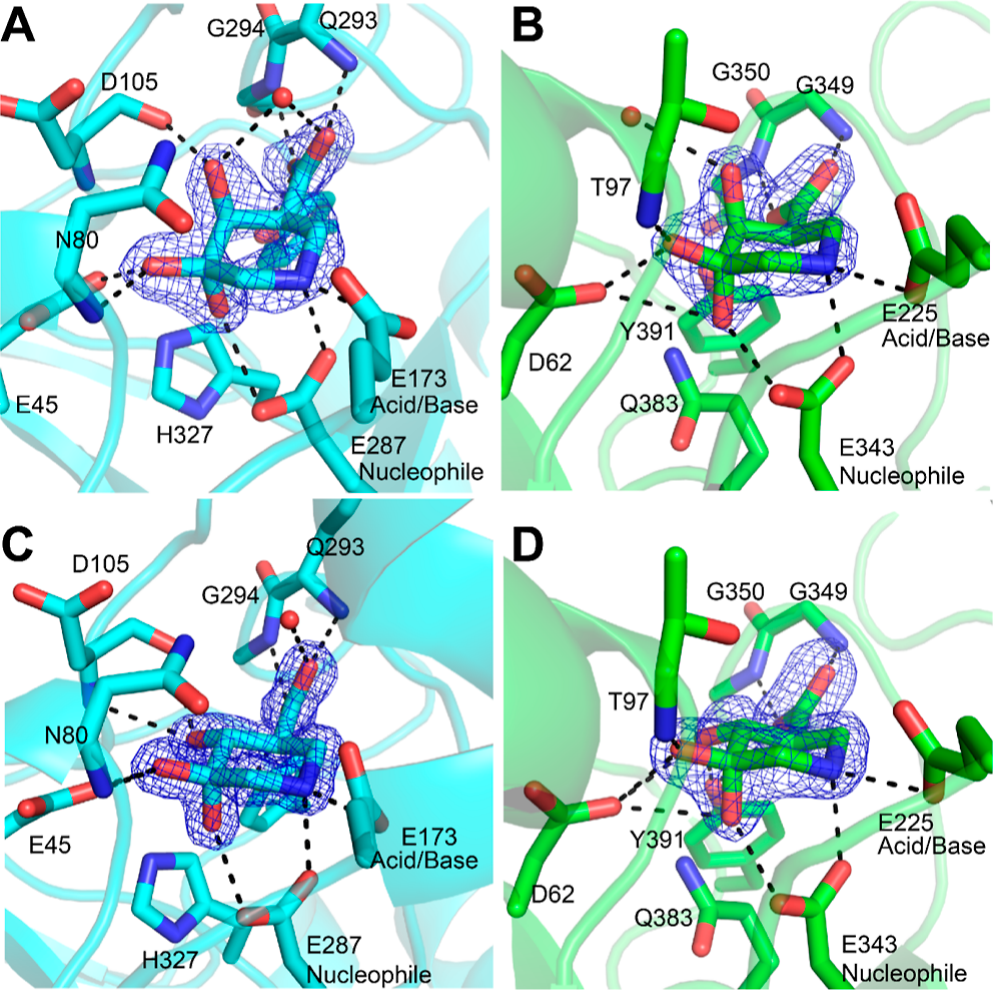
Structures of synthetic iminosugars bound to HPSE and AcGH79. (A) Complex between **8** and AcGH79. (B) Complex between **8** and HPSE. (C) Complex between **9** and AcGH79. (D) Complex between **9** and AcGH79. Electron density (2*F*_o_ – *F*_c_) is shown for the ligand as a blue mesh contoured at 1.5 σ for *A* and *D* (*A* = 0.51 e^–^/Å^3^, *D* = 0.30 e^–^/Å^3^) and 2 σ for *B* and *C* (*B* = 0.45 e^–^/Å^3^, *C* = 0.69 e^–^/Å^3^). The polypeptide is shown in the cartoon form with active site residues shown as sticks. Apparent hydrogen bonding interactions are shown as dotted black lines. Water molecules are shown as red spheres.

To examine the structural basis for inhibition by our panel of synthetic *gluco*- and *galacto*-configured iminosugars, we determined cocrystal structures of the 3-GDIs (**8**, **9**) and either HPSE or AcGH79 (Figure 4). Crystal structures with **8** and **9** bound to AcGH79 show that both of these inhibitors are situated in the same binding pocket as that of the *galacturonic*-noeuromycin **4**. Within the iminosugar ring, there is very little conformational change between **8** and **9**, and both maintain the ^4^C_1_ ring conformation seen for **4**. Furthermore, the axial 4-OH present in **8** forms the same H-bond interactions with the protein backbone (with the carbonyl of Asp105) as seen for **4**; however, it also has an additional H-bond to an active site water, see Figure 4. In the structure between the *gluco*-configured geminal diol **9** and AcGH79, the H-bond between Asp105 at the 4-position is still present—but at a different angle—and additional H-bonds between 4-OH and the backbone nitrogen of Asp105 and the carboxylate of Glu45 are present. These new H-bond interactions are the same as those observed for natural substrates^48^ and other *gluco*-configured inhibitors^16,48^ and are expected to be the cause of the increased inhibition of **9** over **8** for AcGH79. The axial 3-OH of geminal diol inhibitors **8** and **9** is found within hydrogen bonding distance of the catalytic nucleophile in both structures. The axial 3-OH is also within 3.0 Å of the εN of His327 in both structures; however, it is unlikely that there is a genuine 3_ax_-HO···H-εN H-bond interaction as the 3_ax_-OH is oriented approximately 80° to the plane of the histidine–rather than being in line with the plane, as would be expected. Furthermore, the εN of His327 forms an H-bond, at the appropriate angle, with Glu45.

As a point of comparison, we also determined a structure between AcGH79 and *gluco*-configured isofagomine **11** at 1.25 Å resolution, see Figure S10. This inhibitor was positioned nearly identically to geminal diol **9** in the active site of AcGH79. **11** also maintained all of the same H-bond interactions with the protein, with the exception of the H-bond between the axial-3-OH and the nucleophile seen for **9**. The binding of hydroxyl-containing inhibitors to enzyme active sites is associated with an energetic penalty due to desolvation that can be up to 26 kJ mol^–1^, by some estimates.^49^ Although the additional H-bond seen for **9** compensates for the desolvation of the axial hydroxyl, estimates of 18–21 kJ mol^–1^ have been made for the maximal interaction energy of a single hydroxyl H-bond.^50−52^ Therefore, this single interaction is likely not enough to compensate for the additional desolvation energy, and therefore, the removal of the geminal diol is energetically favorable.

Structures of human heparanase soaked with **8** and **9** revealed interactions similar to those seen in the active site of AcGH79. The costructure of **8** soaked into crystals of HPSE showed the inhibitor in the exact same conformation and forming the same protein-inhibitor interactions as the degradation product observed when crystals were soaked with siastatin B. The costructure of **9** and HPSE has hydrogen bonds with the 4_eq_-OH and Tyr391 and Asp62, as opposed to the hydrogen bond with water seen for **8**. The geminal diol **9** has the same hydrogen bonding network as the structure containing **11**, reported by Doherty et al.,^20^ and additional H-bond between the 3_ax_-OH with the nucleophile (Glu343), similar to what was observed in the active site of AcGH79. The 3_ax_-OH in both structures is also within H-bonding distance of Asp62, an additional interaction that is not seen in the AcGH79-geminal diol structures. The 3_ax_-OH in both the **8** and **9** structures is also within 3 Å of the εN of Gln383, however, as for His327 in the active site of AcGH79, this atom is positioned incorrectly to form a hydrogen bond with the axial-hydroxyl. The plane of the amide bond of Gln383 is oriented perpendicular to the axial 3-OH, and the εN of Gln383 already forms two hydrogen bonds with Tyr391 and Asp62. The presence of two additional hydrogen bonds with the active site of HPSE may be able to compensate more fully for the desolvation of an additional hydroxyl in the geminal-diol containing inhibitors. This in turn may be the reason why geminal diol inhibitor **8** is observed in HPSE, when soaked with siastatin B, instead of noeuromycin derivative **4** that is observed in the other β-glucuronidases.

## Discussion

The breakdown products of a synthetic 2-trifluoroacetamido derivative of siastatin B observed by Kondo et al. in the media of enzyme assays are the same hemiaminal **4** and geminal-diol **8** that we observed as breakdown products of siastatin B.^32^ However, the 2-trifluoroacetamido derivative is a more potent inhibitor of β-glucuronidases than the unmodified parent compound.^30,31^ We attribute the enhanced inhibition of 2-trifluoroacetamido-derived siastatin B to the increased rate of elimination that occurs for 2-trifluoroacetamido when compared to the *N*-acetyl group present at the 2-position, this thereby generates the active compounds at higher concentrations than that observed for siastatin B. This “pro-inhibitor” molecular design opens interesting avenues for the future design of glycosidase inhibitors. First, there is potential to optimize the leaving group at the 2-position to generate a molecule that decays into noeuromycin-like and 3-GDIs in hours or days rather than the rapid decay observed for the 2-trifluoroacetamido derivatives of siastatin B, thereby producing a prodrug molecule with a delayed release. Second, there is potential for the generation of prodrug-like molecules that only decay into their active iminosugar form when acted upon by another physiological enzyme—a strategy that could be useful for the development of chaperones for lysosomal storage diseases—or by light-activated decomposition, thereby enabling targeted delivery of inhibitors.

The class of compounds known as noeuromycins were first synthesized and reported by Bols et al. in 2001.^53^ These potent glycosidase inhibitors were inspired by the natural product 1-deoxynojirimycin but were made purely by synthetic means. To the best of our knowledge, there has been no report of a natural product glycosidase inhibitor with a noeuromycin like structure. We however show here that three of the four enzymes that were soaked with siastatin B have a galacturonic acid-configured noeuromycin bound in their active site. This surprising “re-discovery” of noeuromycin-type inhibitors more than 20 years after the first synthesis of this class of molecule is a striking example of the convergence of molecular designs by both man and nature.

## Conclusions

In conclusion, we have shown here through X-ray crystallography that two different types of 1-*N*-iminosugar inhibitors are generated from siastatin B: hemiaminal **4** and 3-GDI **8**. Notably, 3-GDIs are a new class of iminosugar glycoside hydrolase inhibitor, that have been shown to inhibit both GH2 and GH79 β-glucuronidases. Synthesis of 3-GDIs and their 3-hydroxy counterparts allowed us to dissect the mechanism of their inhibition both kinetically and structurally. These 3-GDIs are well accommodated within the active site pocket and form an additional H-bond interaction with the catalytic nucleophile. We also improved the inhibition of these 3-geminal diol inhibitors through epimerization of the 4-hydroxyl. Together these results give new insights into how the broad-spectrum inhibitor siastatin B is able to act on several families of glycoside hydrolase with very different substrate specificities. This information, in turn, should enable the future design of broad-spectrum inhibitors that can target other classes of glycosidase, beyond those targeted by siastatin B.

## Supporting Information Available

Detailed methods for gene expression, protein purification, biochemical assays, structural biology, and complete synthetic protocols; atomic coordinates and structure factors deposited in the Protein Data Bank (PDB ID codes: 8CQI, 8OGX, 8OHQ, 8OHR, 8OHT, 8OHU, 8OHV, 8OHW, and 8OHX); NMR experiments, LC–MS experiments, enzyme kinetics experiments, full-length SDS-page gels, and additional structures; and details on crystal structure data collection and refinement (PDF)
