# Supplementary material for: Molecular Basis for Inhibition of Heparanases and β-Glucuronidases by Siastatin B
Source: J Am Chem Soc. 2023 Dec 20;146(1):125–33. doi: 10.1021/jacs.3c04162 (PMC10785800; doi:10.1021/jacs.3c04162)

Supplemental Information

for

The molecular basis for inhibition of heparanases and β-glucuronidases by siastatin B

Yurong Chen[†,§], Adrianus M. C. H. van den Nieuwendijk[†,§], Liang Wu[‡,#,§], Elisha Moran[‡], Foteini Skoulikopoulou[†], Vera van Riet[†], Hermen S. Overkleeft[†], Gideon J. Davies[‡*], Zachary Armstrong[‡,†,*]

[†]Leiden Institute of Chemistry, Leiden University, Einsteinweg 55, 2300 RA Leiden, The Netherlands

[‡]York Structural Biology Laboratory, Department of Chemistry, The University of York, York YO10 5DD, U.K.

[#]Present Address: Structural Biology, The Rosalind Franklin Institute, Harwell Science & Innovation Campus, Didcot OX11 0QX, U.K;

[§]Authors contributed equally

[*]Corresponding author

Supplemental Figures and Tables

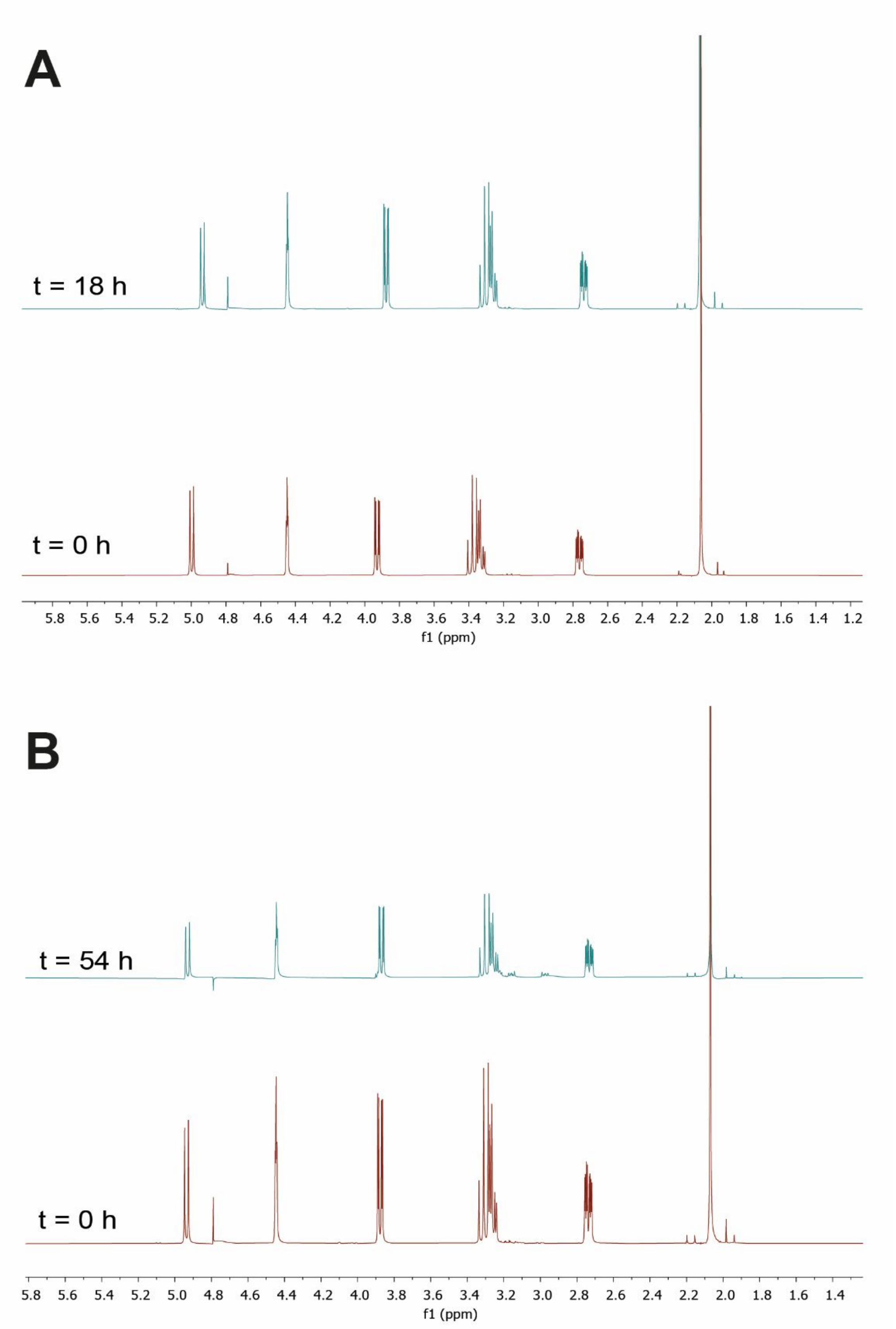

Figure S1. Stability of siastatin B. **A**) ^{1}H NMR experiment showing siastatin B (2 mg) in D_2O phosphate buffer (50 mM, 300 mM NaCl, pH 5.0), taken for two different time points (t=0 min and after 18 h). **B**) Prolonged incubation of siastatin B with recombinant human heparanase. ^{1}H NMR spectra were taken before and after prolonged incubation with 1.2 μM of recombinant human heparanase. The x-axis of each spectra shows the chemical shifts (ppm).Water signal is partially suppressed in both spectra at 4.79 ppm.

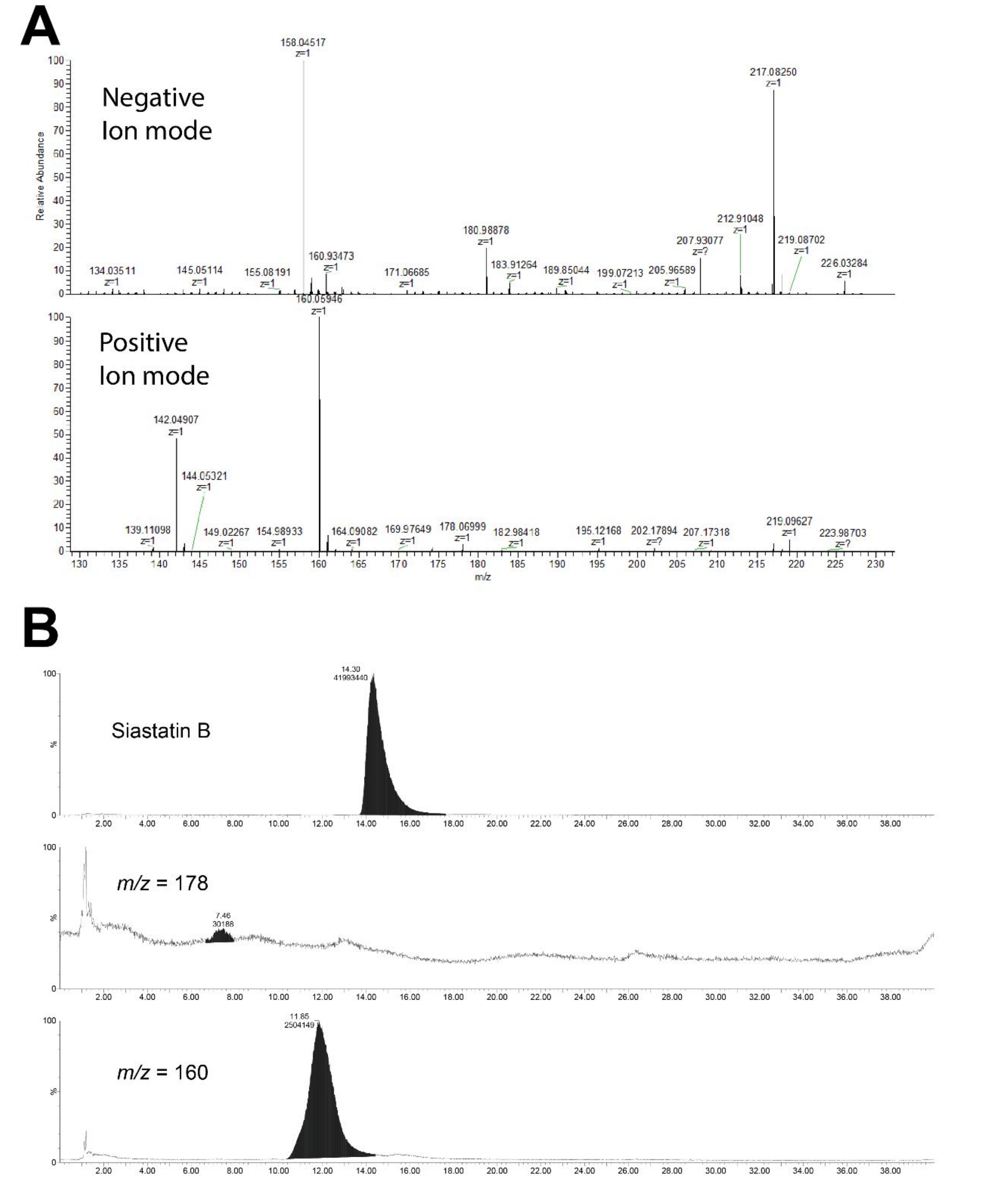

Figure S2. Mass spectrometry of a siastatin B sample dissolved in water. **A)** High-resolution mass spectrometry of a sample of siastatin B was determined using a LTQ Orbitrap mass spectrometer equipped with an electronspray ion source in positive mode (top) and negative ion mode (bottom). **B**) LC-MS of siastatin B dissolved in waster was performed using a Waters UPLC-Xevo-TQS micro equipped with a Acquity UPLC BEH Amide column (100 × 2.1 mm,1.7 µm, Waters, USA). Peaks are labeled with retention time and signal area.

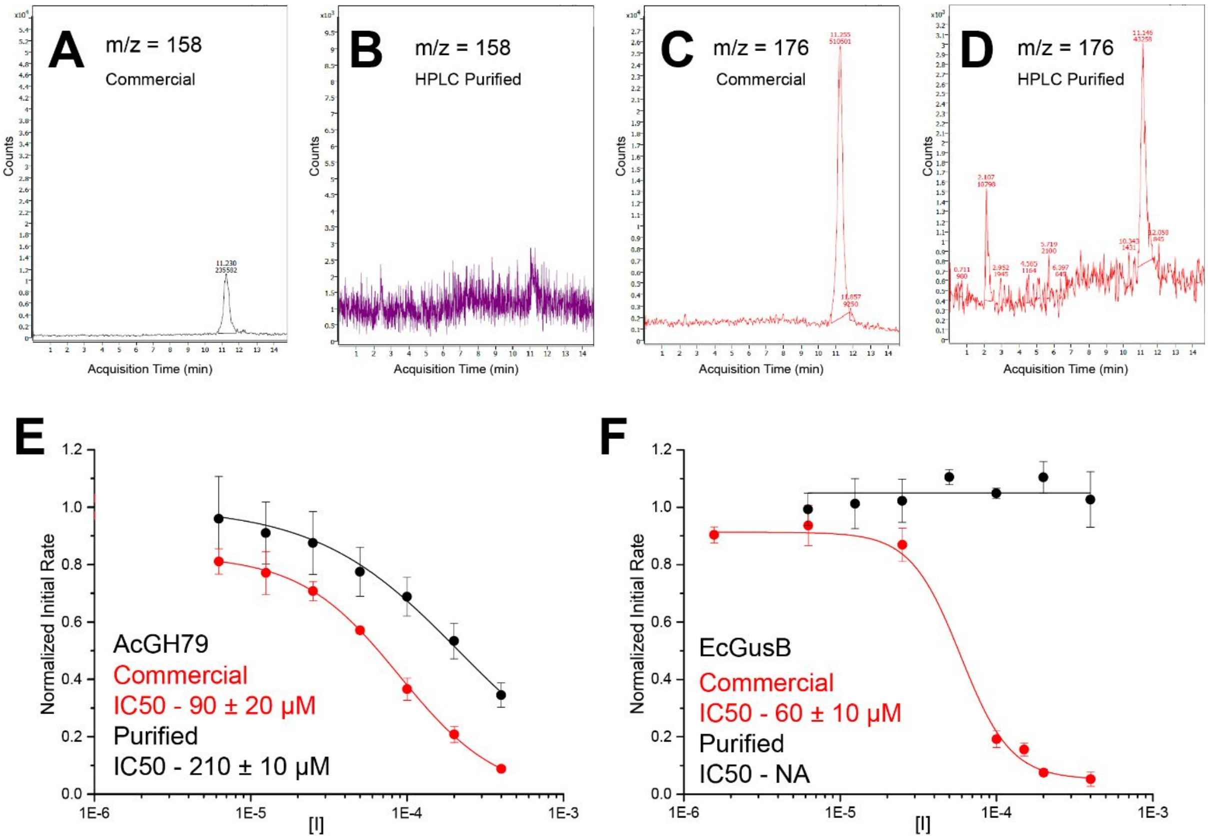

Figure S3. Purification of commercial siastatin B and inhibition of bacterial β-glucuronidases. Ultra-high performance liquid chromatography was performed before (panels **A** and **C**) and after (panels **B** and **D**) purification. These measurements were performed by reverse-phase liquid chromatography using an Agilent 6475 Triple Quadruple LC/MS System equipped with a Nucleodur 100-5 NH2-RP 5um 4.6 x 150 mm column. Peaks are labeled with retention time and signal area. Inhibition of Acidobacterium capsulatum β-glucuronidase (**E**) and E. coli β-glucuronidase (**F**) hydrolysis was determined for both the commercial material (red) and HPLC purified material (black). Curves are fit to a Hill equation. Datapoints are mean ± SD (n ≥ 3).

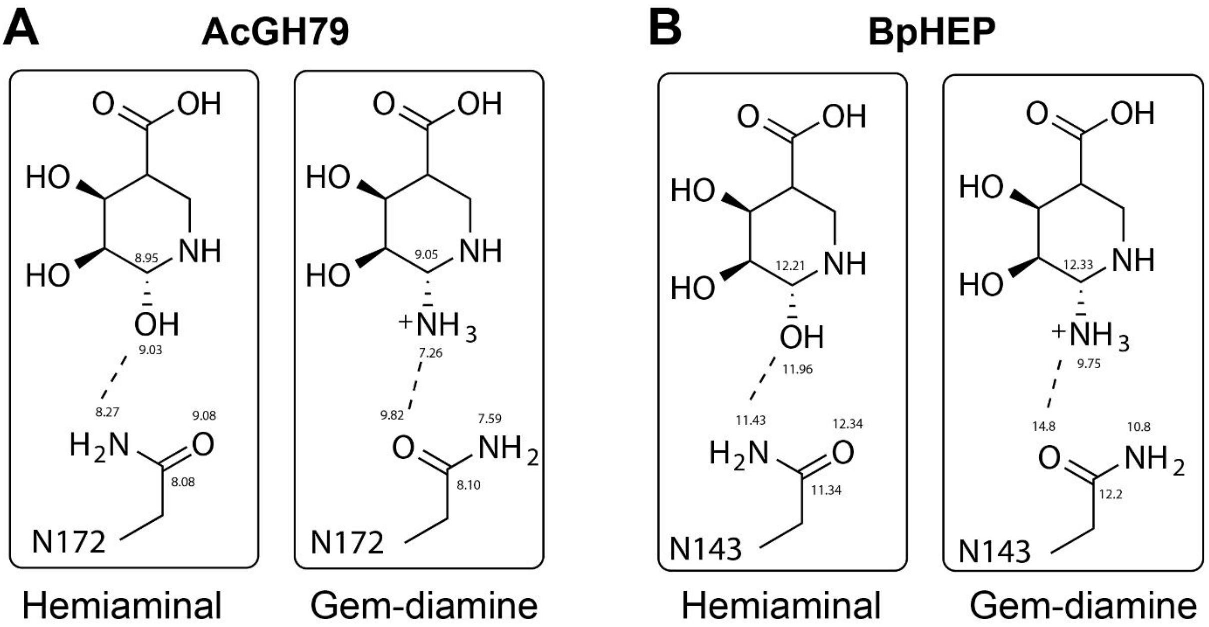

<u>Figure S4.</u> *B*-factor modelling of siastatin B derived inhibitors. Siastatin B derived inhibitors present in the active site of AcGH79 (**A**) or BpHEP (**B**) were modelled as either a hemiaminal or a deacetylated geminal-diamine. B-factors were determined after 40 rounds of refinement with Refmac5 (Ref) and are shown next to the carbon, oxygen or nitrogen atoms under investigation.

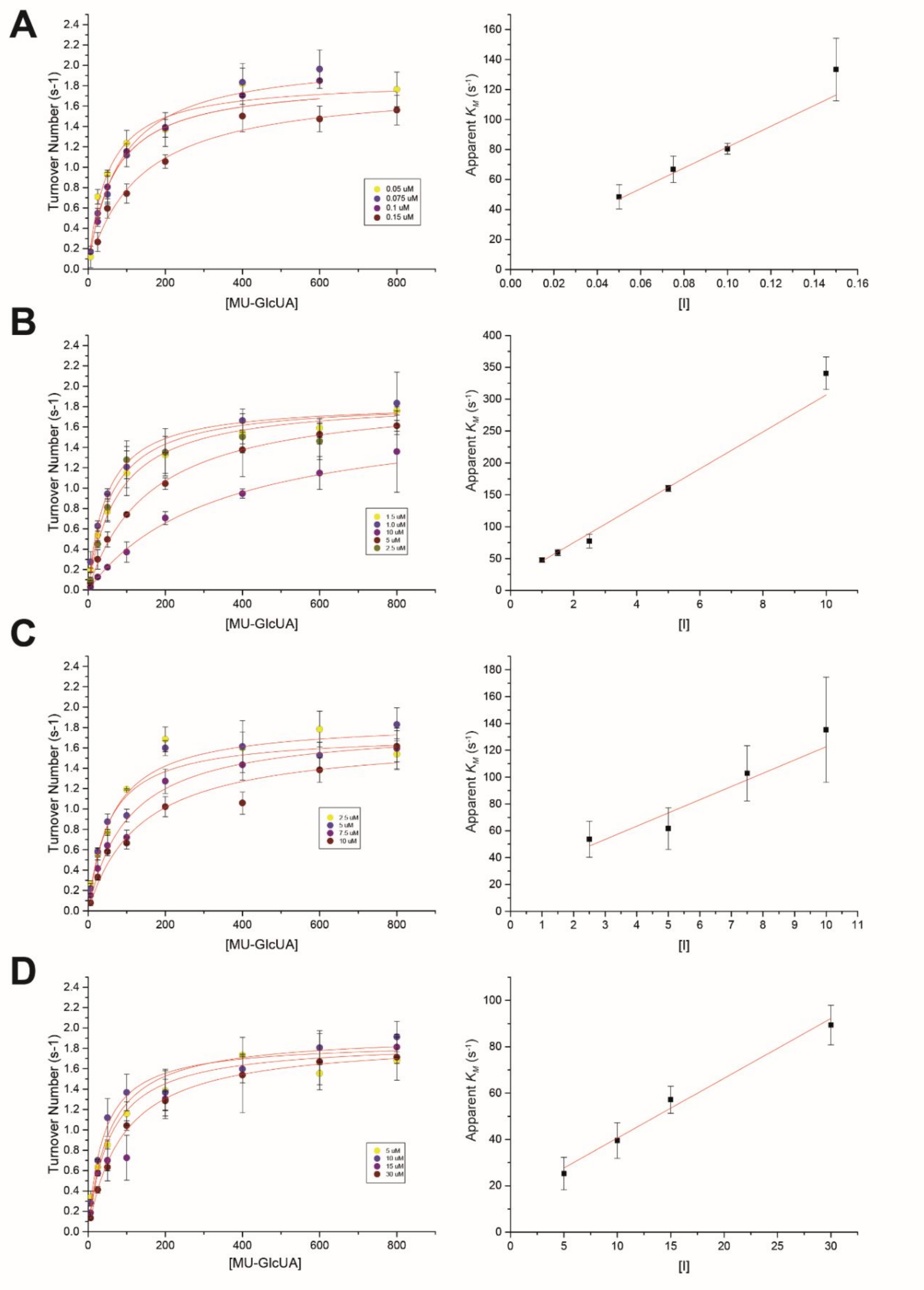

Figure S5. Inhibition kinetics of AcGH79 treated with synthetic inhibitors. The inhibition constants for AcGH79 treated with **A**) **11**, **B**) **9**, **C**) **10**, and **D**) **8** were determined. Michaelis-Menten saturation curves were determined in the presence of various concentration of inhibitor (left panel). These curves were fit to a hyperbolic equation, resulting in a Michaelis constant (K_M). The K_M values were then plotted against the inhibitor concentration (right panel). A linear fit of this plot was then used to determine the inhibition constant (K_I).

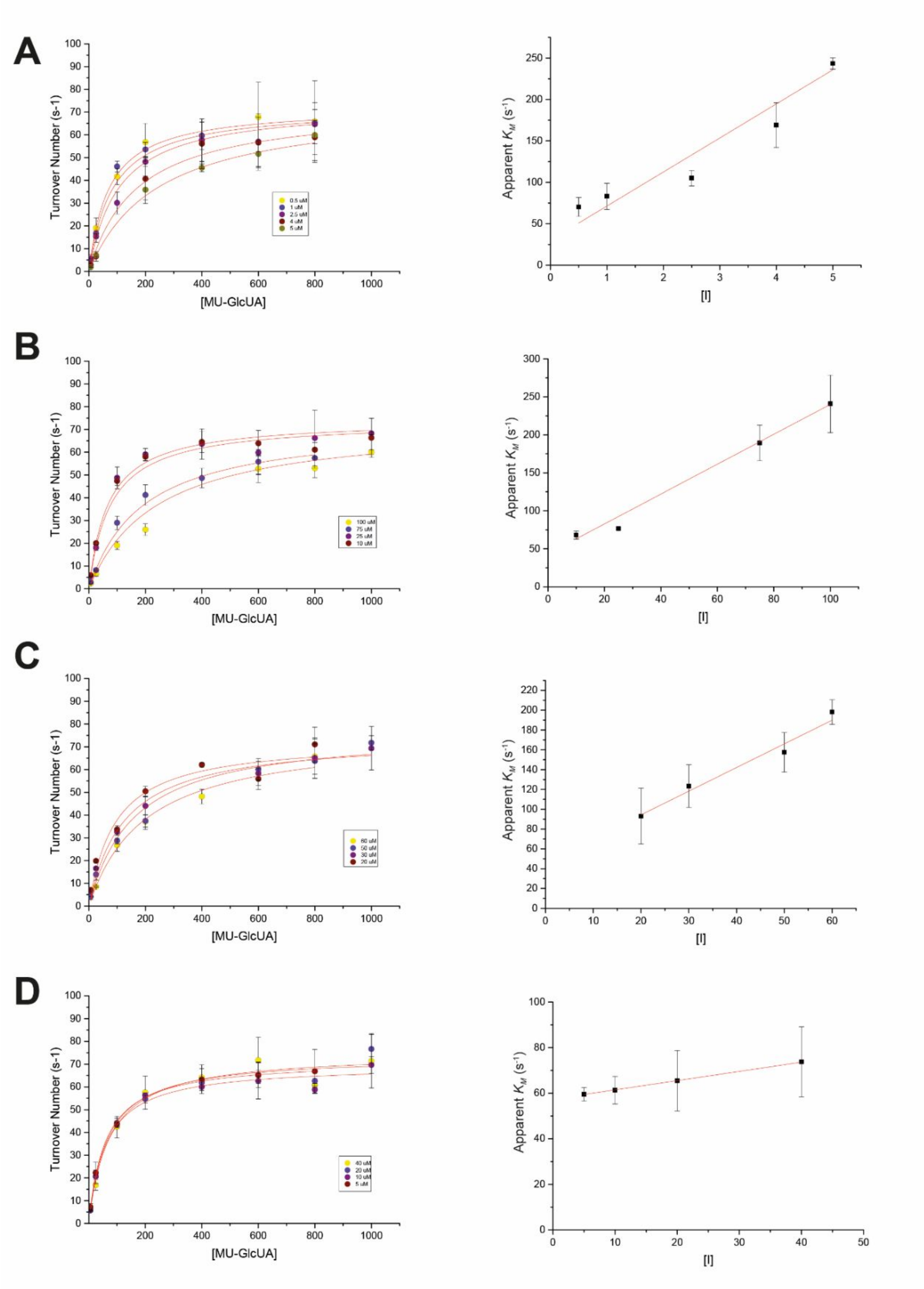

Figure S6. Inhibition kinetics of EcGusB treated with synthetic inhibitors. The inhibition constants for EcGusB treated with **A) 11**, **B) 9**, **C) 10**, and **D) 8** were determined. Michaelis-Menten saturation curves were determined in the presence of various concentration of inhibitor (left panel). These curves were fit to a hyperbolic equation, resulting in a Michaelis constant (K_M). The K_M values were then plotted against the inhibitor concentration (right panel). A linear fit of this plot was then used to determine the inhibition constant (K_I).

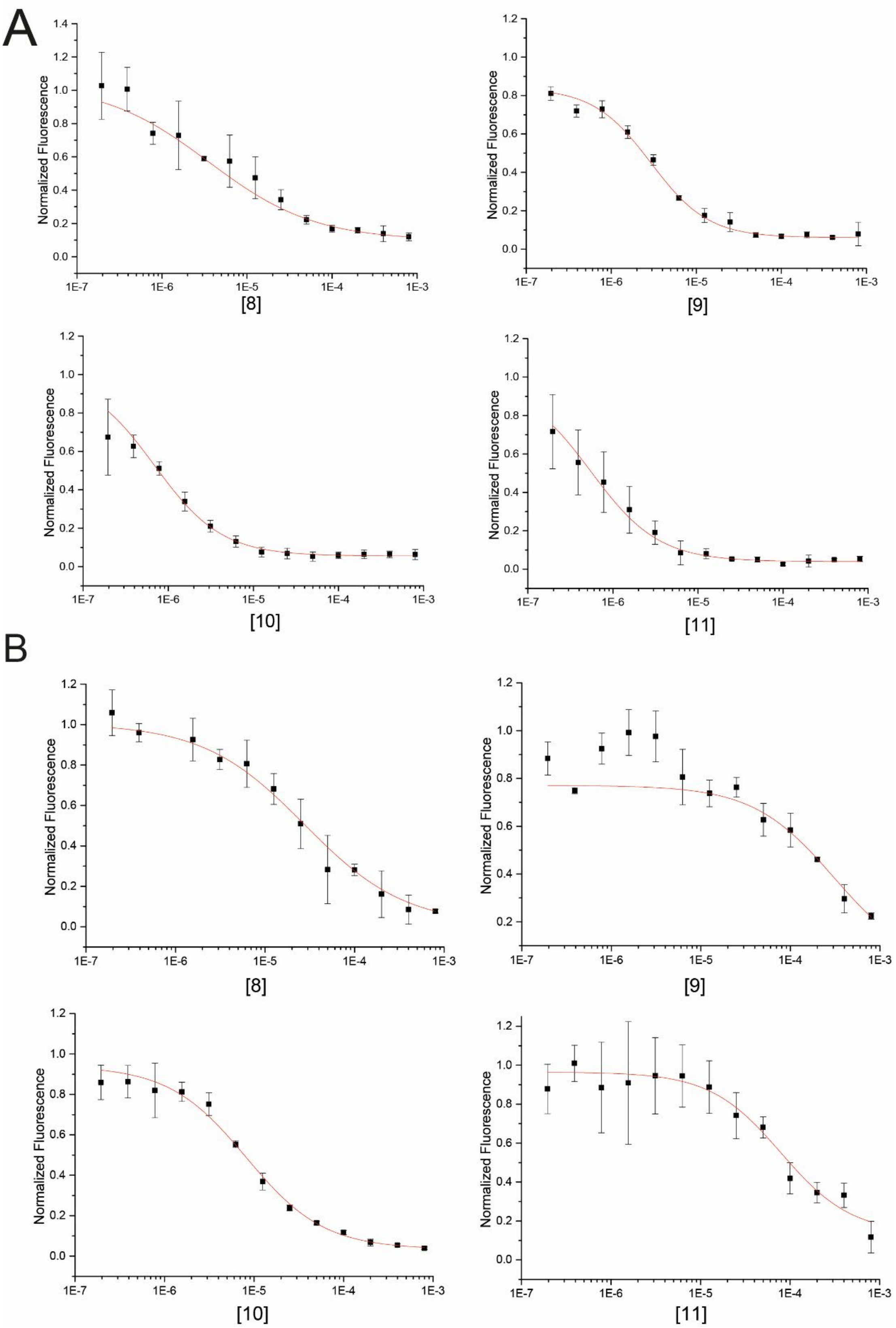

<u>Figure S7.</u> Inhibition of HPSE and GusB in human platelet lysates by inhibitors **8**, **9**, **10** and **11**. **A** shows normalized quantitated band intensities for GusB against inhibitor concentration. **B** shows normalized quantitated band intensities for HPSE against inhibitor concentration. Datapoints are mean ± SD (n ≥ 3).

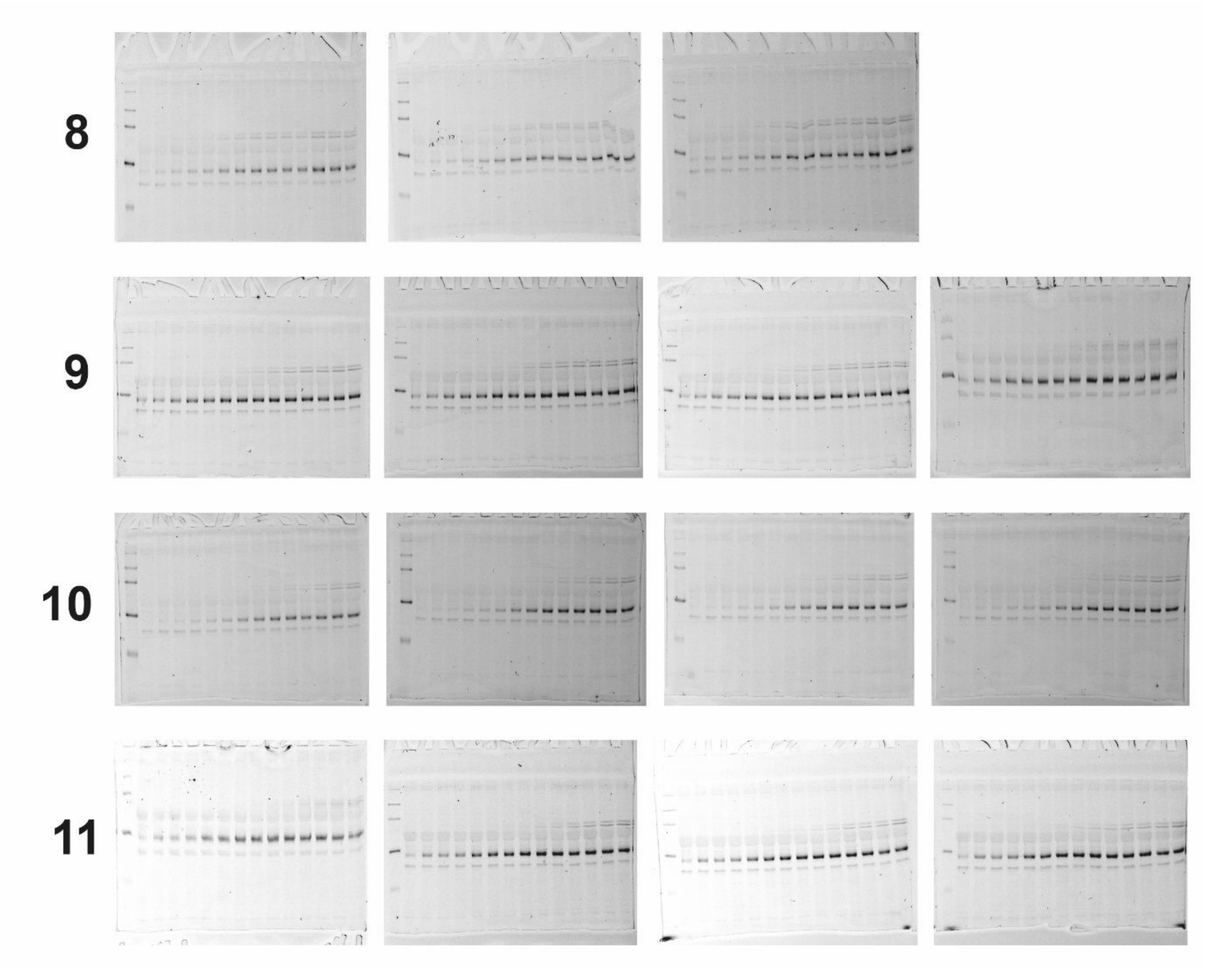

Figure S8. Full length SDS-PAGE gels of residual fluorescent ABP labelling of retaining β-D-glucuronidases in the presence of **8-11** in platelet lysates. Protein loading was visualised with Coomassie Brilliant Blue staining as control (Figure S7).

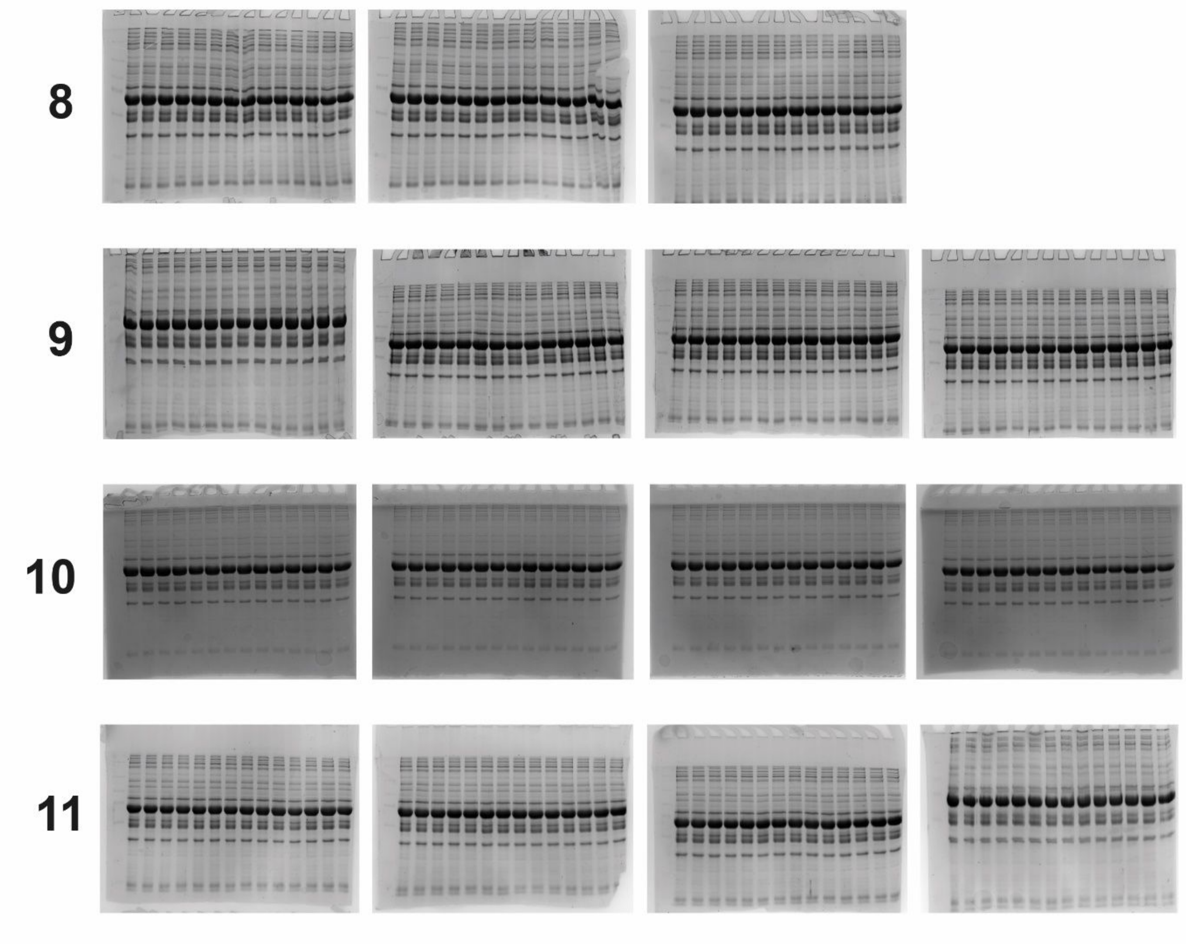

Figure S9. Coomassie Brilliant Blue staining of SDS-PAGE gels for competitive ABPP in platelets shown in Figure S6.

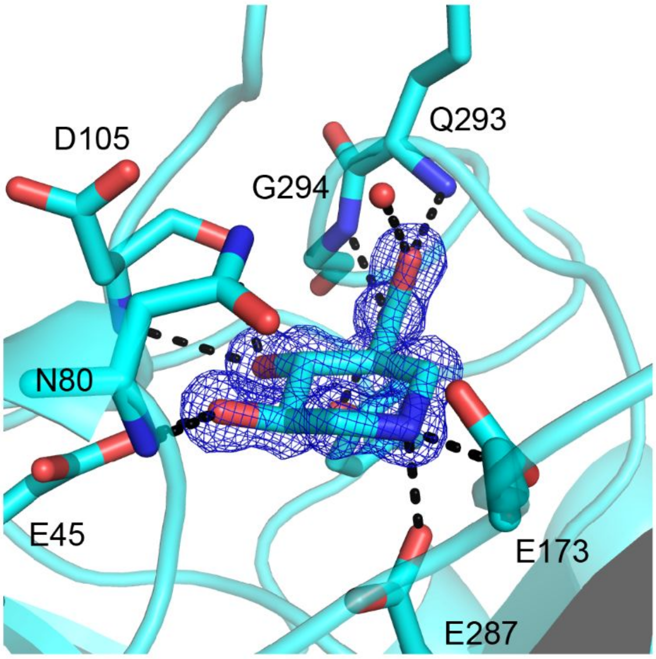

Figure S10. Structure of synthetic iminosugar **11** bound to AcGH79. Electron density ($2F_o - F_c$) is shown for the ligand as a blue mesh contoured at 2 σ (0.85 $e^-/Å^3$). The polypeptide is shown in cartoon form with active site residues shown as sticks. Apparent hydrogen bonding interactions are shown as dotted black lines. Waters molecules are shown as red spheres.

Table 1. Data collection and refinement statistics (molecular replacement)

	HPSE – Siastatin B (PDBID: 8OHQ)	HPSE – [**9**] (PDBID: 8OHR)	HPSE – [**8**] (PDBID: 8CQI)
**Data collection**			
Space group	$P2_1$	$P2_1$	$P2_1$
Cell dimensions			
a, *b*, *c* (Å)	46.11, 70.92, 78.56	44.44, 71.08, 78.23	46.8, 71.5, 79.3
α, β, γ (°)	90.00, 95.53, 90.00	90.00, 98.43, 90.00	90.00, 95.30, 90.00
Resolution (Å)	45.90-1.70 (1.73-1.70)	43.96-1.80 (1.84-1.80)	46.57-2.10 (2.16-2.10)
$I / \sigma I$	10.8 (1.6)	8.8 (1.4)	10.8 (0.8)
Completeness (%)	99.6 (99.9)	99.1 (96.2)	99.7 (100)
Redundancy	1.9 (1.9)	4.6 (4.3)	6.4 (6.6)
$CC_{1/2}$	0.99 (0.60)	0.99 (0.61)	0.99 (0.42)
**Refinement**			
Resolution (Å)	1.70	1.80	2.10
No. reflections	104,973	306,166	30,450
R_{work} / R_{free}	0.17/0.21	0.20/0.23	0.200/0.253
No. atoms			
Protein	3,674	3,643	3,600
Ligand	104/3	34/1	97/3
Water	249	183	135
B-factors			
Protein	29.83	30.97	45.35
Ligand/ion	61.33/ 35.52	42.17/35.28	64.22/54.57
Water	35.04	35.92	43.06
R.m.s. deviations			
Bond lengths (Å)	0.01	0.01	0.02
Bond angles (°)	1.7	1.6	2.0

*Values in parentheses are for highest-resolution shell.

Table 1. Continued.

	AcGH79 – Siastatin B (PDBID: 8OHT)	AcGH79 – **11** (PDBID: 8OHU)	AcGH79 – **9** (PDBID: 8OHV)
**Data collection**			
Space group	$I2_1$	$I2_1$	$I2_1$
Cell dimensions			
a, *b*, *c* (Å)	82.75, 44.67, 136.27	83.05, 44.67, 137.17	82.04, 42.61, 140.14
α, β, γ (°)	90.00, 97.46, 90.00	90.00, 97.61, 90.00	90.00, 99.42, 90.00
Resolution (Å)	67.56-1.05 (1.07-1.05)	42.44-1.18 (1.20-1.18)	40.72-1.50 (1.53-1.50)
$I / \sigma I$	15.1 (1.4)	9.3 (1.1)	11.0 (1.0)
Completeness (%)	96.5 (88.8)	93.7 (53.6)	100 (100)
Redundancy	6.3 (4.5)	5.8 (3.0)	6.4 (6.3)
$CC_{1/2}$	0.99 (0.58)	0.99 (0.38)	0.99 (0.545)
**Refinement**			
Resolution (Å)	1.05	1.25	1.50
No. reflections	1,385,607	840,749	489,904
R_{work} / R_{free}	0.17/0.18	0.16/0.18	0.20/0.22
No. atoms			
Protein	3,613	3,487	3,435
Ligand	17/0	11/0	17
Water	585	357	185
B-factors			
Protein	13.12	15.07	24.02
Ligand/ion	14.48	10.47	22.25
Water	24.68	22.38	26.77
R.m.s. deviations			
Bond lengths (Å)	0.02	0.01	0.01
Bond angles (°)	2.0	1.9	1.8

Table 1. Continued.

	AcGH79 – **8** (PDBID: 8OGX)	BpHEP – Siastatin B (PDBID: 8OHW)	EcGusB – Siastatin B (PDBID: 8OHX)
**Data collection**			
Space group	$I2_1$	$P2_12_12_1$	$I2_1$
Cell dimensions			
a, *b*, *c* (Å)	82.90, 44.70, 137.02	76.37, 104.61, 113.71	126.28, 76.71, 141.02
α, β, γ (°)	90.00, 97.46, 90.00	90.00, 90.00, 90.00	90.00, 102.01, 90.00
Resolution (Å)	41.15-2.00 (2.05-2.00)	61.68-1.27 (1.29-1.27)	67.04-1.95 (1.98-1.95)
$I / \sigma I$	20.5 (7.5)	12.6 (1.1)	8.1 (1.2)
Completeness (%)	99.8 (100)	99.9 (98.5)	98.4 (97.4)
Redundancy	6.4 (6.7)	8.2 (7.4)	4.1 (4.2)
$CC_{1/2}$	0.99 (0.99)	0.99 (0.52)	0.99 (0.72)
**Refinement**			
Resolution (Å)	2.00	1.27	1.95
No. reflections	33,997	1,960,048	384,016
R_{work} / R_{free}	0.19/0.24	0.16/0.18	0.22/0.28
No. atoms			
Protein	3,487	6,441	9,574
Ligand	17/0	56	24
Water	441	1037	316
B-factors			
Protein	19.14	18.44	54.8
Ligand/ion	24.78/0	25.01	42.05
Water	28.35	33.14	43.74
R.m.s. deviations			
Bond lengths (Å)	0.01	0.02	0.01
Bond angles (°)	1.9	1.9	1.6

Supplemental Schemes

The synthesis of compound **12**, a key intermediate in the synthesis of galacturonic acid-type 1-*N*-iminosugars, proceeded through hemiacetal **S1**, which was obtained from commercially available D-lyxose in five steps following procedures described by Ichikawa *et al*[1]. Hydrogenation of the azido group in **S1** using palladium catalyst, followed by *N*-Cbz protection afforded compound **S2**, which was oxidized with Dess-Martin periodinane and then subjected to Wittig methylenation to give **S3**. Hydroboration of **S3** with a sterically bulky hydroborating agent, 9-BBN followed by oxidative work-up finally gave product **12** (Scheme S1).

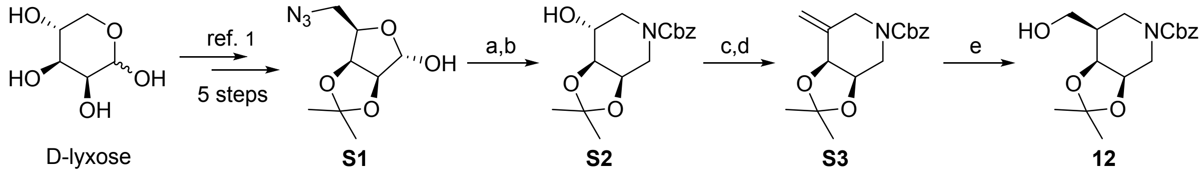

Scheme S1. Synthesis of key intermediate **12**. Reagents and conditions: a) H_2, 10% Pd/C, MeOH, rt; b) CbzCl, sat. aq. $NaHCO_3$, THF, 0 °C to rt, 74% over two steps; c) Dess-Martin periodinane, DCM, 0 °C to rt; d) Ph_3PCH_3Br, ^{t}BuOK, THF, -20 °C to rt, 84% over two steps; (e) *i*) 9-BBN, THF, 0 °C to rt; *ii*) 30% H_2O_2, 1 M NaOH in H_2O, rt, 73%.

The *galacto*-configured geminal diol (**8**) can also be synthesized from *tert*-butyl ester **S4** (Scheme S2). For this purpose, the primary alcohol in **16** (main text, Scheme 1A) was oxidized to a carboxylic acid via a two-step oxidation sequence, followed by esterification with the commercially available *tert*-butyl *N,N'*-diisopropylcarbamimidate in toluene to afford ester **S4**, which after desilylation and subsequent oxidation gave protected ketone **S6**. Unfortunately, reductive deprotection of **S6** by palladium catalyzed hydrogenation under acidic conditions resulted in a complex mixture of compounds. Inspired by the preparation of the *gluco*-configured geminal diol (main text, Scheme 1B), a route via a precursor fully protected with acid-labile groups was investigated. Treatment of compound **S4** with hydrogen and a catalytic amount of Pd/C-10% in methanol for two hours resulted in selective removal of the Cbz group to afford a free amine, which was subsequently protected with a Boc group. After work-up, the resulting Boc-protected intermediate was directly subjected to a second palladium catalyzed hydrogenolysis for prolonged reaction times (48 h) to remove the benzyl group at O4, affording alcohol **S7** which was then treated with MOMCl to give compound **S8**. Desilylation of **S8** and subsequent oxidation yielded ketone **S10** which was deprotected under acidic conditions to afford compound **8**, of which the spectroscopic data were in accordance with those prepared by hydrogenolysis of **19** (main text, Scheme 1A).

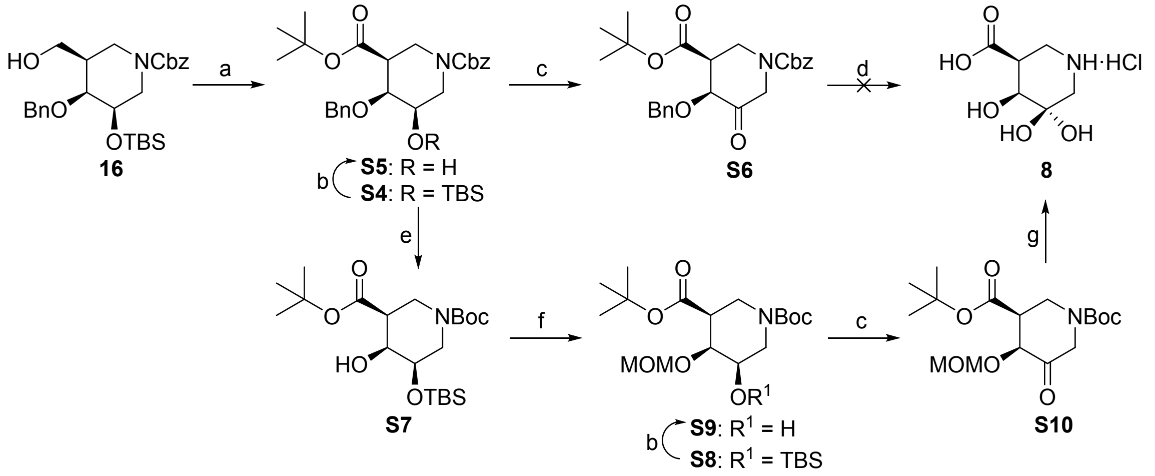

Scheme S2. Alternative synthetic route towards the preparation of compound **8**. Reagents and conditions: a) *i*) Dess-Martin periodinane, DCM, rt; *ii*) $NaClO_2$, NaH_2PO_4, 30% H_2O_2, CH_3CN, H_2O, 0 °C to rt; *iii*) *tert*-butyl *N,N*-diisopropylcarbamimidate, toluene, 60 °C, 71% over three steps; b) TBAF (75 wt% in H_2O), THF, 0 °C, **S5** 96%, **S9** 95%; c) Dess-Martin periodinane, DCM, rt, **S6** 89%, **S10** 80%; d) H_2, 10% Pd/C, H_3O^+, rt; e) *i*) H_2, 10% Pd/C, MeOH, rt, 2 h; *ii*) Boc_2O, DIPEA, DCM, rt; *iii*) H_2, 10% Pd/C, MeOH, 48 h, 69% over three steps; f) MOMCl, DIPEA, DCM, 100 °C, 1 hour in microwave tube, 88%; g) H_3O^+, HFIP, 0 °C to rt, 98%.

The synthesis of glucuronic acid-type 1-*N*-imingugars commenced with the preparation of key intermediate **20** (Scheme S3). Transformation of **S11** into **S15** was achieved using adaptations of the procedures described by Jiang *et al.* for the construction of 1-deoxy-L-fuconojirimycin.[2] Starting from

enantiomerically pure cyanohydrin **S11**, prepared employing (*S*)-hydroxynitrile lyase from the *Hevea brasiliensis* rubber tree,[3] the secondary alcohol was silylated to give **S12** in excellent yield. Conversion of **S12** via a one-pot DIBAL-H reduction-transamination-sodium borohydride reduction cascade sequence, using commercially available allylamine, followed by subsequent *N*-Boc protection, afforded compound **S14** in 81% yield. Ring-closing metathesis of **S14** using Grubbs' catalyst provided heterocyclic alkene **S15**. The last two steps in the synthesis route followed strategies as reported by Takahata *et al.*[4] Epoxidation of **S15** was performed using methyl(trifluoromethyl) dioxirane (generated *in situ* from 1,1,1-trifluroacetone and oxone), and epoxide **S16** was formed preferably due to a favored attack of the dioxirane on the less hindered *anti*-side of the large TBDPS group. Nucleophilic opening of epoxide **S16** was carried out with *in situ* generated $(CH_2{=}CH)_2CuCNLi_2$ in the presence of Lewis acid, affording product **20** in 64% yield.

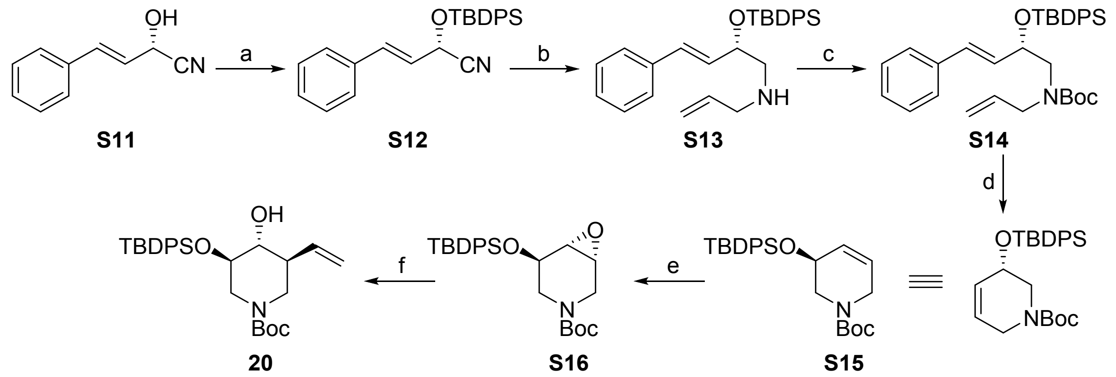

Scheme S3. Synthesis of key intermediate **20**. Reagents and conditions: a) TBDPSCl, imidazole, DMF, 0 °C to rt, 98%; b) *i*) DIBAL-H, Et_2O, -78 °C to 10 °C; *ii*) MeOH, -90 °C; *iii*) allylamine, NaOMe, rt; *iv*) $NaBH_4$, 0 °C to rt; c) Boc_2O, Et_3N, DCM, rt, 81% over two steps; d) Grubbs 1st generation, DCM, reflux, 45%; e) oxone, CH_3COCF_3, $NaHCO_3$, EDTA, MeCN, H_2O, 0 °C, 60%; f) *n*-butyllithium, tetravinyltin, $BF_3{\cdot}Et_2O$, CuCN, Et_2O, -78 °C, 64%.

Materials/Methods

Chemical synthesis

General experimental details

All reagents were of experimental grade and were used without further purification unless stated otherwise. Dichloromethane (DCM) and tetrahydrofuran (THF) were stored over 3 Å molecular sieves and *N, N'*-dimethylformamide (DMF) was stored over 4 Å molecular sieves, which were dried *in vacuo* before use. All reactions were performed under an Argon or N_2 atmosphere unless stated otherwise. Reactions were monitored by analytical thin layer chromatography (TLC) using Merck aluminum sheets pre-coated with silica gel 60 with detection by UV-absorption (254 nm) and by spraying with a solution of $(NH_4)_6Mo_7O_{24}\cdot H_2O$ (25 g/L) and $(NH_4)_4Ce(SO_4)_4\cdot H_2O$ (10 g/mL) in 10% sulfuric acid followed by charring at ~150 °C or by spraying with an aqueous solution of $KMnO_4$ (7%) and K_2CO_3 (2%) followed by charring at ~150 °C. Column chromatography was performed manually using Screening Device b.v. silica gel 60 (0.04-0.063 mm) in the indicated solvents. LC-MS analysis was performed on a LCQ Advantage Max (Thermo Finnigan) ion-trap spectrometer (ESI^+) coupled to a Surveyor HPLC system (Thermo Finnigan) equipped with a C18 column (Gemini, 4.6 mm x 50 mm, 5 μM particle size, Phenomenex). The applied buffers were H_2O, acetonitrile (MeCN) and 1% aqueous trifluoroacetic acid (TFA). ^{1}H-NMR and ^{13}C-NMR spectra were recorded on Bruker AV-400 (400/101 MHz) and Bruker AV-500 (500/126 MHz) spectrometers in the given solvent. Chemical shifts (δ) are given in ppm relative to tetramethylsilane (TMS) as internal standard (^{1}H NMR in $CDCl_3$) or the residual signal of the deuterated solvent. Coupling constants (*J*) are given in Hz. All given ^{13}C-NMR spectra are proton decoupled. The following abbreviations are used to describe peak patterns when appropriate: s (singlet), d (doublet), t (triplet), q (quartet), m (multiplet), Ar (aromatic), C_q (quarternary carbon). 2D NMR experiments (COSY, HSQC) were carried out to assign protons and carbons of the new structures and assignation follows the general numbering shown in compound **S2**. High-resolution mass spectrometry (HRMS) analysis was performed with a LTQ Orbitrap mass spectrometer (Thermo Finnigan), equipped with an electronspray ion source in positive mode (source voltage 3.5 kV, sheath gas flow 10 mL/min, capillary temperature 250 °C) with resolution R = 60000 at m/z 400 (mass range m/z = 150 – 2000) and dioctyl phthalate (m/z = 391.28428) as a "lock mass". The high-resolution mass spectrometer was calibrated prior to measurements with a calibration mixture (Thermo Finnigan).

Experimental Procedures and Characterization Data of Products

The spectroscopic data of known compounds **S1**[1], **S11-S14**[2], **S15-S16**[4] and **20**[4] are in agreement with those previously reported.

Compound S2

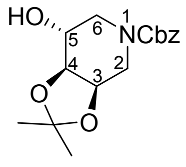

Azide **S1** (10.7 g, 49.8 mmol) was dissolved in MeOH (1000 mL), and nitrogen was bubbled through the solution before 20% $Pd(OH)_2/C$ (2.0 g) was added. While stirring vigorously, the mixture was flushed with two H_2 balloons. After stirring for 20 h under H_2 atmosphere, NMR analysis of a small amount of the reaction mixture revealed almost no conversion. Therefore 10% Pd/C (2.06 g) was added and the reaction was stirred for another 20 h under H_2 atmosphere after which NMR analysis indicated full conversion of the starting material. The reaction mixture was filtered and concentrated to give the iminosugar intermediate (8.2 g, 47 mmol) as a white solid. The crude amine was directly dissolved in a mixture of THF (350 mL) and sat. aq. $NaHCO_3$ (250 mL). After cooling to 0 °C, CbzCl (95%, 10.5 mL, 69.8 mmol) was added dropwise and the reaction was stirred at rt for 40 h until LC-MS analysis indicated full conversion. The layers were separated and the water layer was extracted with EtOAc (2 x 200 mL). The combined organic layers were washed with brine (100 mL), dried over $MgSO_4$, filtered and concentrated *in vacuo*. The product was purified by silica gel column chromatography (pentane/EtOAc 9:1→1:1) affording compound **S2** (11.4 g, 37.1 mmol, 74% over two steps) as a colorless oil. ^{1}H NMR (500 MHz, $CDCl_3$ at 333K) δ 7.43 – 7.14 (m, 5H, CH Ar), 5.12 (s, 2H, CH_2 Cbz), 4.33 – 4.27 (m, 1H, H5), 4.07 (dd, J = 6.5, 4.1 Hz, 1H, H4), 3.97 – 3.83 (m, 2H, H3, H6b), 3.63 (dd, J = 13.5, 3.2 Hz, 1H, H2b), 3.51 (dd, J = 14.2, 3.3 Hz, 1H, H6a), 3.36 (dd, J = 13.5, 5.2 Hz, 1H, H2a), 3.11 (brs, 1H, OH), 1.41 (s, 3H, CH_3), 1.31 (s, 3H, CH_3). ^{13}C NMR (126 MHz, $CDCl_3$ at 333K) δ 156.5 (C=O), 136.9 (C_q Ar), 128.5, 128.0, 127.8 (CH Ar), 109.2 (OCO), 75.6 (C4), 72.0 (C5), 67.5 (C3), 67.3 (CH_2 Cbz), 44.9 (C2), 42.8 (C6), 27.3 (CH_3), 25.0 (CH_3) ppm. HRMS (ESI) m/z: $[M+H]^+$ calc. for $C_{16}H_{22}NO_5$ 308.14925, found 308.14907.

Compound S3

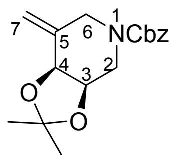

Alcohol **S2** (11.3 g, 36.8 mmol) was dissolved in dry DCM (400 mL) under argon. After cooling to 0 °C, Dess-Martin periodinane (27.3 g, 64.4 mmol) was added in several portions. After stirring for 30 minutes at 0 °C and for 2 h at room temperature, the reaction was diluted with EtOAc (600 mL) and washed successively with 2 M $Na_2S_2O_3$ solution (2 x 250 mL), sat. aq. $NaHCO_3$ (2 x 200 mL) and brine (150 mL). The organic layer was dried over $MgSO_4$, filtrated and concentrated *in vacuo* to afford the crude ketone. In the meantime, methyltriphenylphosphonium bromide (55.3 g, 155 mmol) was suspended in dry THF (250 mL) and cooled to 0 °C, ^{t}BuOK (15.4 g, 138 mmol) was added. The resulting yellow suspension was stirred for 1 h at 0 °C and for 2 h at room temperature. After which, the suspension was cooled to -20 °C and a solution of the crude ketone in dry THF (150 mL) was added dropwise over 20 minutes. The reaction was then stirred overnight at room temperature and quenched by addition of sat. aq. NH_4Cl (250 mL). The layers were separated and the water layer extracted with EtOAc (2 x 100 mL). The combined organic layers were washed with brine (100 mL), dried over $MgSO_4$, filtered and concentrated *in vacuo*. The crude material was purified by silica gel column chromatography (pentane/EtOAc 95:5→3:1)

affording compound **S3** (9.4 g, 31 mmol, 84% over two steps) as a slightly orange oil. ^{1}H NMR (500 MHz, $CDCl_3$ at 333K) δ 7.53 – 7.08 (m, 5H, CH Ar), 5.28 – 5.14 (m, 4H, H7ab and CH_2 Cbz), 4.61 (d, J = 7.4 Hz, 1H, H4), 4.32 (m, 2H, H3 and H6b), 3.97 – 3.76 (m, 2H, H6a and H2b), 3.11 (dd, J = 14.3, 2.8 Hz, 1H, H2a), 1.38 (s, 3H, CH_3), 1.33 (s, 3H, CH_3). ^{13}C NMR (126 MHz, $CDCl_3$ at 333K) δ 155.9 (C=O), 139.7 (C5), 137.0 (C_q Ar), 128.4, 127.9, 127.8 (CH Ar), 116.4 (C7), 109.7 (OCO), 76.3 (C4), 74.6 (C3), 67.1 (CH_2 Cbz), 46.6 (C6), 44.2 (C2), 26.6 (CH_3), 24.8 (CH_3) ppm. HRMS (ESI) m/z: $[M+H]^+$ calc for $C_{17}H_{22}NO_4$ 304.15433, found 304.15419.

Compound 12

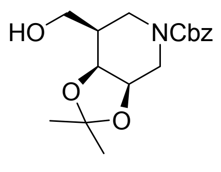

Alkene **S3** (4.70 g, 15.5 mmol) was dissolved in dry THF (150 mL) under argon. After cooling to 0 °C, 9-BBN (0.5 M in THF, 140 mL, 70 mmol) was added dropwise over 30 minutes and the mixture was stirred for 20 h at rt until TLC-analysis indicated full conversion of the starting material. Then the mixture was cooled to 0 °C again, and water (10 mL), 1 M NaOH in H_2O (10 mL) and 30% H_2O_2 (10 mL) were added successively. After stirring overnight at rt, the mixture was diluted with EtOAc (250 mL) and washed with brine (2 x 200 mL). The combined water layers were extracted with EtOAc (100 mL), and the combined organic layers were dried over $MgSO_4$, filtered and concentrated *in vacuo*. The crude was purified by silica gel column chromatography (pentane/EtOAc 1:1→3:7) to afford compound **12** (3.66 g, 11.4 mmol, 73%) as a thick oil. ^{1}H NMR (500 MHz, $CDCl_3$ at 333K) δ 7.37 – 7.22 (m, 5H, Ar), 5.13 (s, 2H, CH_2 Cbz), 4.42 (dd, J = 7.1, 2.7 Hz, 1H, H4), 4.29 (s, 1H, H3), 3.80 (d, J = 10.9 Hz, 1H, H2b), 3.68 (m, 2H, H7ab), 3.56 (dd, J = 12.3, 5.0 Hz, 1H, H6b), 3.29 (d, J = 13.9 Hz, 1H, H2a), 3.22 (app t, J = 12.3 Hz, 1H, H6a), 2.50 (brs, 1H, OH), 1.99 (m, 1H, H5), 1.39 (s, 3H, CH_3), 1.31 (s, 3H, CH_3). ^{13}C NMR (126 MHz, $CDCl_3$ at 333K) δ 156.1 (C=O), 137.0 (C_q Ar), 128.5, 127.9, 127.8 (CH Ar), 108.9 (OCO), 72.6 (C3), 72.4 (C4), 67.1 (CH_2 Cbz), 62.5 (C7), 43.2 (C2), 40.3 (C6), 38.3 (C5), 26.7 (CH_3), 24.7 (CH_3) ppm. HRMS (ESI) m/z: $[M+Na]^+$ calc. for $C_{17}H_{23}NO_5Na$ 344.1468, found 344.1477.

Compound 13

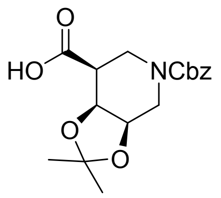

Alcohol **12** (347 mg, 1.08 mmol) was dissolved in dry DCM (14 mL) under argon and Dess-Martin periodinane (720 mg, 1.70 mmol) was added. After stirring at rt for 3 h, TLC-analysis confirmed complete conversion of the starting material. The mixture was diluted with EtOAc (60 mL) and washed successively with aqueous 2 M $Na_2S_2O_3$ (2 x 15 mL), sat. aq. $NaHCO_3$ (2 x 15 mL) and brine (20 mL), dried over $MgSO_4$, filtered and concentrated to afford the crude aldehyde as a colorless oil (350 mg). The aldehyde was directly dissolved in a mixture of MeCN (18 mL) and water (3.5 mL) and $NaH_2PO_4 \cdot 2H_2O$ (1.31 g, 8.40 mmol) was added. The mixture was cooled to 0 °C and 30% H_2O_2 (142 µL, 1.39 mmol) and a solution of $NaClO_2$ (80% purity, 157 mg, 1.14 mmol) in water (8.5 mL) were added subsequently. The slightly yellow solution was stirred at 0 °C for 15 min and for 1 h at rt until TLC-analysis confirmed

full conversion of the aldehyde. The reaction was quenched by addition of Na_2SO_3 (0.5 g) and stirred for another 15 min. After which, the mixture was diluted with EtOAc (60 mL) and the layers were separated. The aqueous layer was extracted with EtOAc (2 x 10 mL) and the combined organic layers were washed with brine (20 mL), dried over $MgSO_4$, filtered and concentrated *in vacuo*. Purification by silica gel column chromatography (pentane/EtOAc 1:1→0:1) afforded the target compound **13** (236 mg, 0.703 mmol, 65%) as a glassy solid. ^{1}H NMR (500 MHz, $CDCl_3$ at 333K) δ 8.14 (broad s, 1H, OH), 7.37 – 7.23 (m, 5H, CH Ar), 5.15 (s, 2H, CH_2 Cbz), 4.72 (dd, J = 6.9, 2.9 Hz, 1H, H4), 4.35 (s, 1H, H3), 3.94 – 3.72 (m, 2H, H6b and H2b), 3.50 (app t, J = 12.7 Hz, 1H, H6a), 3.34 (d, J = 13.6 Hz, 1H, H2a), 2.80 (ddd, J = 12.6, 4.8, 3.2 Hz, 1H, H5), 1.41 (s, 3H, CH_3), 1.33 (s, 3H, CH_3). ^{13}C NMR (126 MHz, $CDCl_3$ at 333K) δ 173.8 (C=O acid), 156.1 (C=O Cbz), 136.8 (C_q Ar), 128.6, 128.1, 128.0 (CH Ar), 109.5 (OCO), 72.3 (C3), 71.9 (C4), 67.5 (CH_2 Cbz), 43.3 (C2), 42.0 (C5), 38.6 (C6), 26.7 (CH_3), 24.8 (CH_3) ppm. HRMS (ESI) m/z: $[M+Na]^+$ calc for $C_{17}H_{21}NO_6Na$ 358.12611, found 358.12580.

Compound 10

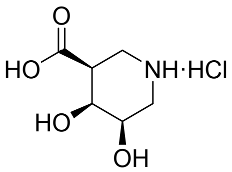

Carboxylic acid **13** (135 mg, 0.403 mmol) was dissolved in a mixture of dioxane (9 mL) and aq. 4 M HCl (1 mL), 10% Pd/C (100 mg) was added subsequently. The mixture was stirred vigorously under a balloon of hydrogen for 16 h, filtered over a Whatman filter and concentrated to give a white residue (130 mg). NMR analysis revealed removal of the isopropylidene acetal while the Cbz-group was still present. Therefore, the residue was re-dissolved in a mixture of THF (10 mL) and water (500 µL), aq. 8 M HCl (100 µL) and 10% Pd/C (139 mg) were added. After stirring vigorously overnight under hydrogen atmosphere, the mixture was filtered over a Whatman filter and concentrated to give a crude product, of which NMR analysis proved removal of the Cbz-group. The residue was then dissolved in absolute ethanol (1.2 mL) and added dropwise to a stirred solution of dry diethyl ether (20 mL) to afford a white precipitate. The precipitate was collected on a filter, washed with dry diethyl ether (2 x 5 mL) and dried *in vacuo* to give target compound **10** (85 mg, quant) as an off-white powder. ^{1}H NMR (400 MHz, D_2O at 293K) δ 4.42 (s, 1H, H4), 3.99 (dt, J = 11.2, 3.6 Hz, 1H, H3), 3.40 (dd, J = 12.9, 4.4 Hz, 1H, H6b), 3.29 – 3.18 (m, 2H, H2b and H6a), 3.09 – 2.99 (m, 2H, H2a and H5). ^{13}C NMR (101 MHz, D_2O at 293K) δ 173.3 (C=O), 66.8 (C4), 65.3 (C3), 42.7 (C5), 41.9 (C2), 38.5 (C6). HRMS (ESI) m/z: $[M+H]^+$ calc. for $C_6H_{12}NO_4$ 162.07608, found 162.07572.

Compound 14

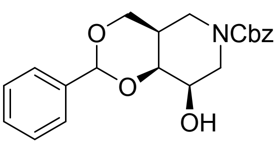

To a stirred solution of alcohol **12** (3.65 g, 11.4 mmol) in MeOH (100 mL) was added 8 M HCl solution (2.24 mL) and the mixture stirred at room temperature overnight until TLC-analysis confirmed full conversion of the starting material. The reaction was quenched by addition of Et_3N (5 mL). The

solvent was evaporated and the residue treated with EtOAc (100 mL) and filtered. The filter cake was washed with EtOAc (3 x 10 mL) and the combined filtrates were concentrated to afford the crude triol that was directly dissolved in DMF (80 mL). After addition of benzaldehyde dimethyl acetal (3.40 mL, 22.6 mmol) and (+)-camphorsulfonic acid (1.57 g, 6.76 mmol), the mixture was stirred at 60 °C for 20 h until LC-MS analysis showed full conversion. After cooling to rt, sat. aq. $NaHCO_3$ (300 mL) was added and the mixture was extracted with EtOAc (3 x 130 mL). The combined organic layers were washed with brine (2 x 50 mL), dried over $MgSO_4$, filtered and concentrated *in vacuo*. The crude was purified by silica gel column chromatography (pentane/EtOAc 4:1→1:1) affording compound **14** (3.03 g, 8.20 mmol, 72% over two steps) as a colorless oil. ^{1}H NMR (400 MHz, $CDCl_3$ at 293K) δ 7.45 (dd, J = 6.6, 3.1 Hz, 2H, CH Ar), 7.42 – 7.26 (m, 8H, CH Ar), 5.53 (s, 1H, CHPh), 5.12 (s, 2H, CH_2 Cbz), 4.29 – 3.89 (m, 5H, H4, H2b, H6b and H7ab), 3.62 (s, 1H, H3), 3.38 (m, 1H, H6a), 2.95 – 2.82 (m, 1H, H2a), 2.60 (d, J = 10.5 Hz, 1H, OH), 1.79 – 1.59 (m, 1H, H5). ^{13}C NMR (101 MHz, $CDCl_3$ at 293K) δ 155.4 (C=O), 137.9 (C_q Ar), 137.2 (C_q Ar), 129.4, 128.6, 128.4, 128.2, 128.0, 126.3 (CH Ar), 101.8 (OCHO), 75.9 (C4), 68.6 (C7), 67.7 (C3), 67.4 (CH_2 Cbz), 44.9 (C2), 41.2 (C6), 33.8 (C5) ppm. HRMS (ESI) m/z: $[M+H]^+$ calc. for $C_{21}H_{24}NO_5$ 370.1649, found 344.1644.

Compound 15

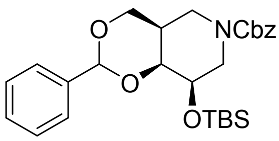

Alcohol **14** (2.35 g, 6.36 mmol) was dissolved in DCM (35 mL) and imidazole (1.96 g, 28.8 mmol) and TBSCl (2.3 g, 15 mmol) were added subsequently. The resulting suspension was stirred for 20 h at room temperature. The mixture was diluted with EtOAc (150 mL), washed with water and brine, dried over $MgSO_4$, filtered and concentrated in *vacuo*. The crude product was purified by silica gel column chromatography (pentane/EtOAc 95:5→9:1) to afford target compound **15** (2.88 g, 5.95 mmol, 94%) as a colorless oil. ^{1}H NMR (500 MHz, $CDCl_3$ at 333K) δ 7.48 (d, J = 7.4 Hz, 2H, CH Ar), 7.38 – 7.23 (m, 8H, CH Ar), 5.51 (s, 1H, CHPh), 5.18 (d, J = 12.4 Hz, 1H, CH*H* Cbz), 5.10 (d, J = 12.4 Hz, 1H, CH*H* Cbz), 4.13 (s, 1H, H4), 4.08 – 3.89 (m, 4H, H7ab, H6b and H2b), 3.68 (d, J = 9.9 Hz, 1H, H3), 3.45 (t, J = 12.5 Hz, 1H, H6a), 3.08 (t, J = 11.5 Hz, 1H, H2a), 1.64 (d, J = 10.1 Hz, 1H, H5), 0.88 (s, 9H, $(CH_3)_3C$), 0.06 (s, 6H, $2CH_3$). ^{13}C NMR (126 MHz, $CDCl_3$ at 333K) δ 155.7 (C=O), 138.8, 137.1 ($2C_q$ Ar), 128.9, 128.7, 128.3, 128.2, 128.1, 126.2 (CH Ar), 101.5 (CHPh), 77.2 (C4), 69.9 (C3), 69.0 (C7), 67.4 (CH_2 Cbz), 45.1 (C2), 41.8 (C6), 34.7 (C5), 25.9 ((CH$_3$)$_3$C), 18.3 ((CH_3)$_3$$C$), -4.4 ($CH_3$), -4.3 ($CH_3$) ppm. HRMS (ESI) m/z: $[M+Na]^+$ calc. for $C_{27}H_{37}NO_5SiNa$ 506.2333, found 506.2338.

Compound 16

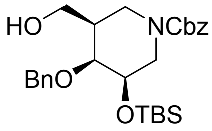

Compound **15** (2.59 g, 5.36 mmol) was dissolved in dry DCM (60 mL) and cooled on an ice-bath. $BH_3 \cdot THF$ (1.0 M in THF, 22 mL, 22 mmol) was added dropwise in five minutes and followed by addition of TMSOTf (0.16 mL, 0.88 mmol). The

resulting solution was stirred for 10 minutes on the ice-bath and for 3 h at room temperature. TLC-analysis confirmed full conversion and the reaction was quenched with TEA (2.5 mL) at 0 °C followed by careful addition of MeOH (25 mL). After stirring for another 1 h, the reaction was concentrated, co-evaporated twice with MeOH to give the crude product. Purification by silica gel column chromatography (pentane/EtOAc 4:1→1:1) afforded target compound **16** (2.53 g, 5.21 mmol, 97%) as a colorless oil. ^{1}H NMR (500 MHz, $CDCl_3$ at 333K) δ 7.35 – 7.22 (m, 10H, CH Ar), 5.18 (d, J = 12.4 Hz, 1H, CH*H* Cbz), 5.05 (d, J = 12.4 Hz, 1H, CH*H* Cbz), 4.94 (d, J = 11.5 Hz, 1H, CH*H* Bn), 4.59 (d, J = 11.5 Hz, 1H, CH*H* Bn), 3.85 – 3.75 (m, 2H, H4 and H2b), 3.74 – 3.65 (m, 2H, H3 and H6b), 3.62 (m, 2H, H7ab), 3.38 – 3.30 (m, 1H, H2a), 3.13 (dd, J = 12.9, 10.7 Hz, 1H, H6a), 2.05 (s, 1H, OH), 1.84 (s, 1H, H5), 0.91 (s, 9H, $(CH_3)_3C$), 0.10 (s, 6H, $2CH_3$). ^{13}C NMR (126 MHz, $CDCl_3$ at 333K) δ 155.7 (C=O), 139.1, 137.0 ($2C_q$ Ar), 128.5, 128.5, 128.0, 128.0, 127.9, 127.7 (CH Ar), 77.7 (C4), 74.1 (CH_2 Bn), 71.7 (C3), 67.3 (CH_2 Cbz), 61.9 (C7), 46.1 (C2), 42.4 (C5), 41.7 (C6), 25.9 ((CH$_3$)$_3$C), 18.1 ($(CH_3)_3C$), -4.7, -4.7 ($2CH_3$) ppm. HRMS (ESI) m/z: $[M+H]^+$ calc. for $C_{27}H_{40}NO_5Si$ 486.26703, found 486.26696.

Compound 17

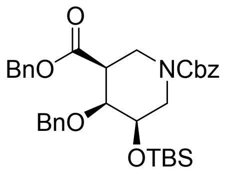

To a stirred solution of alcohol **16** (930 mg, 1.92 mmol) in acetone (40 mL) was added 2.4 M Jones reagent dropwise until an orange/brown color persisted. The reaction was stirred for an additional 10 min and then quenched by the addition of 2-propanol (3.0 mL). Subsequently sat. aq. $NaHCO_3$ (50 mL) and water (50 mL) were added and the acetone was carefully (foaming) evaporated from the mixture. Next acetic acid was added drop by drop until pH = 5. EtOAc (50 mL) was added and the mixture stirred vigorously for a few minutes after which the mixture was filtered over a pad of celite. The layers were separated and the water layer extracted with EtOAc (50 mL). The combined organic layers were dried over $MgSO_4$, filtered and concentrated to give the crude carboxylic acid (0.95 g) as a colorless oil. The crude acid was dissolved in DCM (20 mL) and benzyl alcohol (540 μL, 5.1 mmol) and DMAP (33 mg, 0.27 mmol) were added. After cooling on an ice-bath, DIC (320 μL, 2.05 mmol) was added dropwise and the mixture stirred for one more hour on the ice-bath and for 6 h at rt. At that time TLC indicated circa 50% conversion, so the reaction was left overnight. Extra portions of benzyl alcohol (540 μL) and DIC (320 μL) were added and the reaction stirred for an extra 6 h upon which TLC did not show any further progress of the reaction. The mixture was concentrated and purified by column chromatography (pentane/EtOAc 95:5→4:1) to afford benzyl ester **17** as a colorless oil (520 mg, 0.882 mmol) in 46% yield. ^{1}H NMR (500 MHz, $CDCl_3$ at 333K) δ 7.34 – 7.16 (m, 15H, CH Ar), 5.17 (d, J = 12.4 Hz, 1H, CH*H* Cbz), 5.11 (d, J = 12.3 Hz, 1H, CH*H* Bn_{ester}), 5.07 (d, J = 12.4 Hz, 1H, CH*H* Cbz), 4.99 (d, J = 12.3 Hz, 1H, CH*H* Bn_{ester}), 4.95 (d, J = 11.3 Hz, 1H, CH*H* Bn), 4.47 (d, J = 11.3 Hz, 1H, CH*H* Bn), 4.26 (s, 1H, H6b), 4.23 (s, 1H, H4), 3.98 (s, 1H, H2b), 3.68 (d, J = 7.6 Hz, 1H, H3), 3.29 (app t, J = 12.7 Hz, 1H, H6a), 3.13 (app t, J = 11.6 Hz, 1H, H2a), 2.61 (d, J = 9.7 Hz, 1H, H5), 0.91 (s, 9H,

$(CH_3)_3C$), 0.10 (s, 6H, $2CH_3$) ppm. ^{13}C NMR (126 MHz, $CDCl_3$ at 333K) δ 170.3 (OC=O), 155.4 (NC=O), 139.3, 136.9, 135.9 ($3C_q$ Ar), 128.6, 128.6, 128.4, 128.2, 128.1, 128.0, 127.4, 127.4 (CH Ar), 77.9 (C4), 75.2 (CH_2 Bn), 71.5 (C3), 67.4 (CH_2 Cbz), 66.6 (CH_2 Bn_{ester}), 46.3 (C5), 45.0 (C2), 39.9 (C6), 25.9 ((CH$_3$)$_3$C), 18.1 ((CH_3)$_3$$C$), -4.6, -4.7 ($2CH_3$) ppm. HRMS (ESI) m/z: $[M+H]^+$ calc. for $C_{34}H_{44}NO_6Si$ 590.29324, found 590.29347.

Compound 18

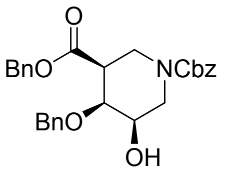

To an ice-cold solution of TBS-ether **17** (325 mg, 0.551 mmol) in THF (8 mL) was added TBAF (75 wt% in H_2O, 420 mg, 1.20 mmol) and the mixture was left stirring on the ice bath. After 5 h TLC-analysis showed complete conversion of the starting material. The mixture was diluted with EtOAc (40 mL), washed with brine (2 x 15 mL), dried over $MgSO_4$, filtered and concentrated *in vacuo*. The crude product was purified by column chromatography (pentane/EtOAc 95:5→7:3) to afford alcohol **18** (247 mg, 0.519 mmol, 94%) as a colorless oil. ^1H NMR (400 MHz, $CDCl_3$ at 293K) δ 7.36 – 7.12 (m, 15H, CH Ar), 5.07 (m, 4H, CH_2 Cbz and CH_2 Bn_{ester}), 4.60 (m, 1H, CH*H* Bn), 4.48 (d, J = 11.6 Hz, 1H, CH*H* Bn), 4.26 – 3.83 (m, 3H, H4, H6b and H2b), 3.61 (brs, 1H, H3), 3.35 (app. t, J = 12.3 Hz, 1H, H6a), 3.12 – 2.78 (m, 2H, H2a and OH), 2.61 (brs, 1H, H5). ^{13}C NMR (101 MHz, $CDCl_3$ at 293K) δ 170.7 (OC=O), 155.2 (NC=O), 138.3, 136.4, 135.2 ($3C_q$ Ar), 128.6, 128.5, 128.5, 128.5, 128.4, 128.1, 127.9, 127.7, 127.4 (CH Ar), 76.9 (C4), 74.3 (CH_2 Bn), 68.5 (C3), 67.4 (CH_2 Cbz), 66.8 (CH_2 Bn_{ester}), 45.6 & 45.3 (C5), 45.2 & 44.9 (C2), 39.7 (C6). HRMS (ESI) m/z: $[M+Na]^+$ calc. for $C_{28}H_{29}NO_6Na$ 498.18871, found 498.18891.

Compound 19

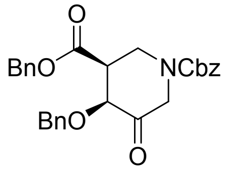

To a stirred solution of compound **18** (225 mg, 0.474 mmol) in DCM (9 mL) was added Dess-Martin periodinane (60%, 682 mg, 0.965 mmol) and the mixture was stirred at rt for 3 h. The reaction mixture was then diluted with EtOAc (50 mL), washed with aq. 2 M $Na_2S_2O_3$ (2 x 15 mL), sat. aq. $NaHCO_3$ (2 x 15 mL) and brine (15 mL), dried over $MgSO_4$, filtered and concentration *in vacuo*. The crude ketone was purified by silica gel column chromatography (pentane/EtOAc 4:1→7:3) to afford ketone **19** (160 mg, 0.338 mmol, 71%) as a colorless oil. ^1H NMR (400 MHz, $CDCl_3$ at 293K) δ 7.37 – 7.14 (m, 15H, CH Ar), 5.19 – 4.94 (m, 4H, CH_2 Cbz and CH_2 Bn_{ester}), 4.69 (d, J = 12.0 Hz, 1H, CH*H* Bn), 4.52 – 4.41 (m, 1H, CH*H* Bn), 4.35 – 3.77 (m, 5H, H2ab, H6ab and H4), 3.16 (brs, 1H, H5). ^{13}C NMR (101 MHz, $CDCl_3$ at 293K) δ 200.3 (C=O), 169.5 (OC=O), 154.9 (NC=O), 136.8, 136.0, 135.2 ($3C_q$ Ar), 128.5, 128.5, 128.4, 128.3, 128.3, 128.1, 127.8, 127.4 (CH Ar), 78.31 (C4), 72.4 (CH_2 Bn), 67.8 (CH_2 Cbz), 67.1 (CH_2 Bn_{ester}), 52.7 (C2), 46.4 (C5), 41.8 (C6) ppm. HRMS (ESI) m/z: $[M+Na]^+$ calc. for $C_{28}H_{29}NO_6Na$ 496.17306, found 496.17304.

Compound S4

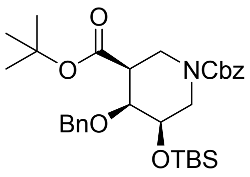

Alcohol **16** (1.25 g, 2.58 mmol) was dissolved in dry DCM (25 mL). Dess-Martin periodinane (60% purity, 2.65 g, 3.75 mmol) was added and the reaction was stirred at room temperature for 2 h. The reaction mixture was diluted with EtOAc (100 mL) and washed successively with sat. aq. $Na_2S_2O_3$ (2 x 50 mL), sat. aq. $NaHCO_3$ (2 x 50 mL) and brine (50 mL). The organic layer was dried over $MgSO_4$, filtered and concentrated to afford the crude aldehyde (1.47 g). To a stirred solution of the crude aldehyde in MeCN (45 mL) and water (8.5 mL), $NaH_2PO_4 \cdot 2H_2O$ (3.12 g, 20.0 mmol) was added and the mixture was stirred at rt until it turned homogeneous. After which, the reaction was cooled to 0 °C and 30% H_2O_2 (340 µL, 4.35 mmol) and a solution of $NaClO_2$ (80% purity, 375 mg, 3.31 mmol) in water (8.5 mL) were added. The mixture was stirred at 0 °C for 15 minutes and at rt for 1 h until TLC-analysis confirmed full conversion. The reaction was quenched by addition of Na_2SO_3 (0.95 g) and stirred for 10 more minutes, the mixture was diluted with EtOAc (60 mL) and the layers were separated. The water layer was extracted with EtOAc (2 x 30 mL) and the combined organic layers were washed with brine (30 mL), dried over $MgSO_4$, filtered and concentrated to afford the crude carboxylic acid (1.47 g). The crude acid was co-evaporated twice with dry toluene and then dissolved in dry toluene (10 mL). *tert*-Butyl *N, N'*-diisopropylcarbaimidate (1.94 g, 9.70 mmol) was added and the reaction stirred at 60 °C for 5 h. LC-MS analysis confirmed complete conversion of the acid and the mixture was concentrated and filtered over a plug of silica gel eluting with pentane/Et_2O (9:1) to afford the crude ester. Purification by silica gel column chromatography (pentane/Et_2O 98:2→9:1) afforded target compound **S4** (1.01g, 1.82 mmol, 71% over three steps) as a colorless oil. ^1H NMR (500 MHz, $CDCl_3$ at 333K) δ 7.33 – 7.24 (m, 9H, CH Ar), 7.22 – 7.16 (m, 1H, CH Ar), 5.18 (d, J = 12.4 Hz, 1H, CH*H* Cbz), 5.10 – 5.01 (m, 2H, CH*H* Cbz and CH*H* Bn), 4.59 (d, J = 11.1 Hz, 1H, CH*H* Bn), 4.26 – 4.16 (m, 2H, H4 and H6b), 3.98 (s, 1H, H3), 3.67 (d, J = 7.3 Hz, 1H, H2b), 3.20 (t, J = 12.7 Hz, 1H, H6a), 3.13 (t, J = 11.6 Hz, 1H, H2a), 2.52 – 2.44 (m, 1H, H5), 1.40 (s, 9H, $(CH_3)_3CO$), 0.91 (s, 9H, $(CH_3)_3CSi$), 0.11 (s, 3H, CH_3Si), 0.10 (s, 3H, CH_3Si). ^{13}C NMR (126 MHz, $CDCl_3$ at 333K) δ 169.5 (OC=O), 155.3 (NC=O), 139.3, 136.9 (2C_q Ar), 128.5, 128.1, 128.0, 127.9, 127.2 (CH Ar), 81.2 ($(CH_3)_3$*C*O), 78.1 (C4), 75.2 (CH_2 Bn), 71.6 (C3), 67.2 (CH_2 Cbz), 46.9 (C5), 44.9 (C2), 40.0 (C6), 28.1 ((*C*H_3)$_3$CO), 25.8 ((*C*H_3)$_3$CSi), 18.0 ($(CH_3)_3$*C*Si), -4.7, -4.8 (2CH_3Si) ppm. HRMS (ESI) m/z: $[M+Na]^+$ calc. for $C_{31}H_{45}NO_6SiNa$ 578.29084, found 578.29100.

Compound S5

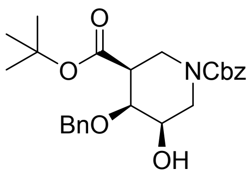

To an ice-cold solution of TBS-ether **S4** (0.35 g, 0.63 mmol) in THF (8 mL) was added TBAF (75 wt% in H_2O, 430 mg, 1.24 mmol) and the mixture was left stirring on the ice bath. After 5 h TLC-analysis showed complete conversion of the TBS-ether. The mixture was diluted with EtOAc (40 mL), washed with brine (2 x 10 mL), dried over $MgSO_4$, filtered and concentrated *in vacuo*. The crude was purified by silica gel column chromatography (pentane/EtOAc 9:1→4:1) to afford the title alcohol **S5** (267 mg, 0.605

mmol, 96%) as a colorless oil. ^{1}H NMR (500 MHz, $CDCl_3$ at 333K) δ 7.34 – 7.19 (m, 10H, CH Ar), 5.11 (s, 2H, CH_2 Cbz), 4.73 (dd, J = 11.4 Hz, 2H, CH_2 Bn), 4.20 (s, 1H, H4), 4.06 (dd, J = 13.5, 4.4 Hz, 1H, H6b), 3.92 (d, J = 8.9 Hz, 1H, H2b), 3.61 (s, 1H, H3), 3.32 (dd, J = 13.5, 11.0 Hz, 1H, H6a), 3.11 – 3.03 (m, 1H, H2a), 2.78 (d, J = 7.6 Hz, 1H, OH), 2.54 – 2.46 (m, 1H, H5), 1.43 (s, 9H, $(CH_3)_3C$). ^{13}C NMR (126 MHz, $CDCl_3$ at 333K) δ 170.1 (OC=O), 155.4 (NC=O), 138.6, 136.8 (2C_q Ar), 128.5, 128.4, 128.0, 127.8, 127.7, 127.4 (CH Ar), 81.7 ($(CH_3)_3\mathit{C}$), 77.3 (C4), 74.4 (CH_2 Bn), 68.8 (C3), 67.3 (CH_2 Cbz), 46.5 (C5), 45.5 (C2), 40.2 (C6), 28.1 ($(\mathit{C}H_3)_3C$) ppm. HRMS (ESI) m/z: $[M+Na]^+$ calc. for $C_{25}H_{31}NO_6Na$ 464.20436, found 464.20419.

Compound S6

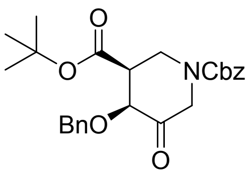

Compound **S5** (135 mg, 0.306 mmol) was dissolved in dry DCM (6 mL) and Dess-Martin periodinane (252 mg, 0.594 mmol) was added. The reaction was stirred at rt for 2 h until TLC-analysis indicated complete conversion of the alcohol. The mixture was diluted with EtOAc (25 mL) and washed subsequently with aq. 2 M $Na_2S_2O_3$ (2 x 10 mL), sat. aq. $NaHCO_3$ (2 x 10 mL) and brine (10 mL), dried over $MgSO_4$, filtered and concentrated *in vacuo*. The crude was purified by silica gel column chromatography (pentane/EtOAc 4:1→7:3) to afford ketone **S6** (119 mg, 0.271 mmol, 89%) as a colorless oil. ^{1}H NMR (400 MHz, $CDCl_3$ at 333K) δ 7.39 – 7.21 (m, 10H, CH Ar), 5.10 (s, 2H, CH_2 Cbz), 4.76 (d, J = 12.1 Hz, 1H, CH*H* Bn), 4.57 (d, J = 12.1 Hz, 1H, CH*H* Bn), 4.30 (d, J = 17.0 Hz, 1H, H2b), 4.08 (br. s, 1H, H6b), 4.00 (d, J = 4.5 Hz, 1H, H4), 3.96 (br. s, 1H, H2a), 3.79 (d, J = 11.4 Hz, 1H, H6a), 3.05 (s, 1H, H5), 1.38 (s, 9H, $(CH_3)_3C$). ^{13}C NMR (101 MHz, $CDCl_3$ at 293K) δ 200.6 (C=O), 168.8 (OC=O), 154.8 (NC=O), 137.0, 136.0 (2C_q Ar), 128.6, 128.5, 128.3, 128.1, 128.0, 127.8 (CH Ar), 82.4 ($(CH_3)_3\mathit{C}$), 78.8 (C4), 72.4 (CH_2 Bn), 67.8 (CH_2 Cbz), 52.9 (C2), 47.4 (C5), 42.3 (C6), 27.8 ($(\mathit{C}H_3)_3C$) ppm. HRMS (ESI) m/z: $[M+Na]^+$ calc. for $C_{25}H_{31}NO_6Na$ 462.18871, found 462.18832.

Compound S7

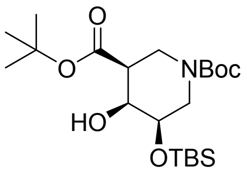

Compound **S4** (700 mg, 1.26 mmol) was dissolved in methanol (25 mL) under argon and 10% Pd/C (140 mg) was added. While stirring vigorously, the mixture was flushed with a H_2 balloon. After stirring for 2 h under H_2 atmosphere TLC and LC-MS analyses confirmed complete removal of the Cbz-group. The mixture was filtered over a Whatman filter and concentrated. The crude residue was directly taken up in DCM (10 mL) and DIPEA (250 µL, 1.44 mmol) and Boc_2O (700 mg, 3.21 mmol) were added. The mixture was stirred at rt overnight until LC-MS analysis confirmed complete *N*-Boc protection. The mixture was concentrated and eluted over a small plug of silica gel (pentane / EtOAc = 7 / 3) and the eluate was concentrated again. The material was redissolved in methanol (20 mL) under argon and 10% Pd/C (140 mg) was added. After stirring for 16 h under H_2 atmosphere, LC-MS indicated only circa 20% conversion of the starting material, so more 10% Pd/C (280 mg) and 20% $Pd(OH)_2/C$ (300 mg)

were added and the mixture was stirred over the weekend until LC-MS analysis indicated full conversion. The mixture was filtered over a Whatman filter and concentrated *in vacuo*. The crude material was purified by silica gel column chromatography (pentane/EtOAc 9:1→4:1) to afford compound **S7** (372 mg, 0.862 mmol, 69% over three steps) as a colorless oil. ^{1}H NMR (500 MHz, $CDCl_3$ at 333K) δ 4.20 (s, 1H, H4), 4.02 (d, J = 11.3 Hz, 1H, H6b), 3.85 (d, J = 7.4 Hz, 1H, H2b), 3.60 (ddd, J = 10.6, 5.2, 2.8 Hz, 1H, H3), 3.11 (t, J = 12.7 Hz, 1H, H6a), 2.88 (t, J = 10.9 Hz, 1H, H2a), 2.44 (m, 2H, H5 and OH), 1.47 (s, 9H, $(CH_3)_3CO$), 1.46 (s, 9H, $(CH_3)_3CO$), 0.91 (s, 9H, $(CH_3)_3CSi$), 0.13 (s, 6H, $2CH_3Si$). ^{13}C NMR (126 MHz, $CDCl_3$ at 333K) δ 169.9 (OC=O), 154.7 (NC=O), 81.2, 79.9 ($(CH_3)_3\mathit{C}O$), 69.5 (C4), 69.4 (C3), 46.1 (C5), 44.1 (C2), 39.1 (C6), 28.4, 28.1 ($(\mathit{C}H_3)_3CO$), 25.8 ($(\mathit{C}H_3)_3CSi$), 18.1 ($(CH_3)_3\mathit{C}Si$), -4.6 (CH_3Si), -4.8 (CH_3Si) ppm. HRMS (ESI) m/z: $[M+Na]^+$ calc. for $C_{21}H_{41}NO_6SiNa$ 454.25954, found 454.25934.

Compound S8

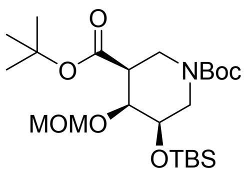

In a microwave tube compound **S7** (0.18 g, 0.42 mmol) was dissolved in a mixture of dry DCM (4 mL), DIPEA (1.5 mL) and MOMCl (0.5 mL). The tube was sealed and heated for 1 h at 100 ^{o}C in a microwave (Biotage initiator+). After which the mixture was diluted with EtOAc (25 mL), washed with water (10 mL), sat. aq. $NaHCO_3$ (10 mL) and brine (10 mL), dried over $MgSO_4$, filtered and concentrated. The crude was purified by silica gel column chromatography (pentane/EtOAc 98:2→95:5) to give target compound **S8** (176 mg, 0.370 mmol, 88%) as a colorless oil. ^{1}H NMR (500 MHz, $CDCl_3$ at 333K) δ 4.81 (d, J = 5.8 Hz, 1H, CH*H*O MOM), 4.77 (d, J = 5.8 Hz, 1H, CH*H*O MOM), 4.19 (s, 1H, H4), 4.10 (d, J = 9.3 Hz, 1H, H6b), 3.86 (d, J = 9.1 Hz, 1H, H2b), 3.56 (ddd, J = 10.8, 4.9, 2.2 Hz, 1H, H3), 3.35 (s, 3H, OCH_3), 3.09 (t, J = 12.8 Hz, 1H, H6a), 2.99 (t, J = 11.3 Hz, 1H, H2a), 2.43 (ddd, J = 12.0, 4.5, 2.0 Hz, 1H, H5), 1.47 (s, 9H, $(CH_3)_3CO$), 1.46 (s, 9H, $(CH_3)_3CO$), 0.92 (s, 9H, $(CH_3)_3CSi$), 0.12 (s, 6H, $2CH_3Si$). ^{13}C NMR (126 MHz, $CDCl_3$ at 333K) δ 169.6 (OC=O), 154.8 (NC=O), 98.2 (CH_2 MOM), 81.3 ($(CH_3)_3\mathit{C}O$), 79.9 ($(CH_3)_3\mathit{C}O$), 76.0 (C4), 71.3 (C3), 56.2 (CH_3 MOM), 46.9 (C5), 44.8 (C2), 39.5 (C6), 28.5 ($(\mathit{C}H_3)_3CO$), 28.2 ($(\mathit{C}H_3)_3CO$), 25.9 ($(\mathit{C}H_3)_3CSi$), 18.2 ($(CH_3)_3\mathit{C}Si$), -4.6 (CH_3Si), -4.8 (CH_3Si) ppm. HRMS (ESI) m/z: $[M+H]^+$ calc. for $C_{23}H_{46}NO_7Si$ 476.30381, found 476.30378.

Compound S9

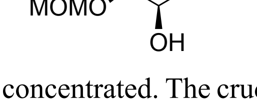

Compound **S8** (175 mg, 0.368 mmol) was dissolved in THF (5 mL) and cooled on an ice-bath. TBAF (75 wt% in H_2O, 230 mg, 0.66 mmol) was added and the mixture was stirred on the ice bath for 5 h. The mixture was then diluted with EtOAc (30 mL), washed with brine (2 x 10 mL), dried over Na_2SO_4, filtered and concentrated. The crude was purified by silica gel column chromatography (pentane/EtOAc 95:5→ 7:3) to afford compound **S9** (127 mg, 0.351 mmol, 95%) as a colorless oil. ^{1}H NMR (500 MHz, $CDCl_3$ at 333K) δ 4.72 (d, J = 6.5 Hz, 1H, CH*H* MOM), 4.68 (d, J = 6.5 Hz, 1H, CH*H* MOM), 4.19 (t, J = 2.5

Hz, 1H, H4), 4.10 (d, *J* = 10.6 Hz, 1H, H6b), 4.00 – 3.86 (m, 2H, OH and H2b), 3.52 – 3.45 (m, 1H, H3), 3.44 (s, 3H, CH_3 MOM), 3.08 (dd, *J* = 13.5, 11.6 Hz, 1H, H6a), 2.83 (app t, *J* = 11.7 Hz, 1H, H2a), 2.54 (ddd, *J* = 11.4, 4.8, 2.5 Hz, 1H, H5), 1.46 (s, 18H, $2(CH_3)_3CO$). ^{13}C NMR (126 MHz, $CDCl_3$ at 333K) δ 169.6 (OC=O), 154.9 (NC=O), 98.9 (CH_2 MOM), 81.4 ($(CH_3)_3\mathit{C}O$), 80.8 (C4), 80.1 ($(CH_3)_3\mathit{C}O$), 67.8 (CH_3 MOM), 56.2 (C3), 46.7 (C5), 45.5 (C2), 39.7 (C6), 28.5, 28.2 ($(\mathit{C}H_3)_3CO$) ppm. HRMS (ESI) m/z: $[M+Na]^+$ calc. for $C_{17}H_{31}NO_7Na$ 384.19927, found 384.19904.

Compound S10

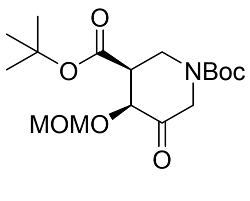

To a stirred solution of alcohol **S9** (126 mg, 0.349 mmol) in dry DCM (5 mL) was added Dess-Martin periodinane (248 mg, 0.585 mmol) and the mixture was stirred at rt for 3 h until complete conversion was confirmed by TLC and LC-MS analyses. The mixture was diluted with EtOAc (30 mL) and washed subsequently with aq. 2 M $Na_2S_2O_3$ (2 x 10 mL), sat. aq. $NaHCO_3$ (2 x 10 mL) and brine (10 mL), dried over $MgSO_4$, filtered and concentrated *in vacuo*. The crude was purified by silica gel column chromatography (pentane/acetone 98:2→85:15) to afford ketone **S10** (100 mg, 0.278 mmol, 80%) as a colorless oil. ^1H NMR (500 MHz, $CDCl_3$ at 333K) δ 4.76 (d, *J* = 6.8 Hz, 1H, CH*H* MOM), 4.73 (d, *J* = 6.8 Hz, 1H, CH*H* MOM), 4.31 (dd, *J* = 17.4, 1.2 Hz, 1H, H2b), 4.22 – 4.13 (m, 2H, H4 and H6b), 3.87 (d, *J* = 17.4 Hz, 1H, H2a), 3.68 (dd, *J* = 14.1, 4.2 Hz, 1H, H6a), 3.38 (s, 3H, CH_3 MOM), 3.13 (dd, *J* = 9.6, 4.8 Hz, 1H, H5), 1.45 (s, 9H, $(CH_3)_3CO$), 1.43 (s, 9H, $(CH_3)_3CO$). ^{13}C NMR (126 MHz, $CDCl_3$ at 333K) δ 200.3 (C=O), 169.2 (OC=O), 154.1 (NC=O), 96.6 (CH_2 MOM), 82.4, 81.0 ($(CH_3)_3\mathit{C}O$), 77.5 (C4), 56.1 (CH_3 MOM), 53.6 (C2), 47.7 (C5), 43.1 (C6), 28.4, 28.0 ($(\mathit{C}H_3)_3CO$) ppm. HRMS (ESI) m/z: $[M+Na]^+$ calc. for $C_{17}H_{31}NO_7Na$ 382.18362, found 382.18337.

Compound 8

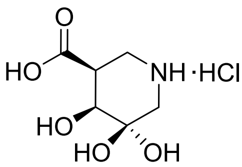

Method A (deprotection of ketone **19**): Ketone **19** (68 mg, 0.14 mmol) was dissolved in a mixture of THF (10 mL) and aqueous 3 M HCl (300 µL). Subsequently, 10% Pd/C (72 mg) was added and the mixture was stirred under hydrogen atmosphere for 18 h. The mixture was filtered over a Whatman® filter and concentrated *in vacuo* to afford the target compound (31 mg) as a white foam in quantitative yield.

Method B (deprotection of ketone **S10**): Ketone **S10** (43 mg, 0.120 mmol) was dissolved in a mixture of hexafluoro-2-propanol (1.5 mL) and water (0.25 mL) and cooled on an ice-bath. Subsequently, aqueous 6 M HCl (0.25 mL) was added and the mixture was allowed to warm-up and stirred overnight. The mixture was concentrated using a water aspirator, and the residue was dissolved in water (5 mL) and washed with *tert*-butyl methyl ether (5 x 3 mL). Water was evaporated again and the residue lyophilized from water to afford the target compound (25 mg) in 98% yield. ^1H NMR (500 MHz, D_2O at 293K) [hydrate form] δ 4.20 – 4.15 (m, 1H, H4), 3.44 (dd, *J* = 12.1, 3.8 Hz, 1H, H6b), 3.32 (ddd, *J* = 12.7, 3.8, 2.5 Hz, 1H, H5), 3.26 (d, *J* = 12.4 Hz, 1H, H6a), 3.21 (d, *J* = 12.7 Hz, 1H, H2b), 3.16 (d, *J*

= 12.7 Hz, 1H, H2a). ^{13}C NMR (126 MHz, D_2O) δ 173.3 (C=O), 91.1 (C3), 69.4 (C4), 46.1 (C2), 41.9 (C5), 38.4 (C6). HRMS (ESI) m/z: $[M_{hydrate}+H]^+$ calc. for $C_6H_{12}NO_5$ 178.07100, found 178.07068.

Compound 21

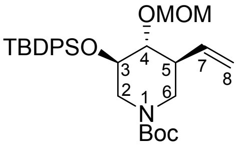

Compound **20** (218 mg, 0.454 mmol) was co-evaporated with toluene (3 x) and dissolved in dry DCM (3.0 mL) in a microwave tube. After cooling to 0 °C, DIPEA (632 µL, 3.63 mmol) and MOMCl (241 µL, 3.18 mmol) were added successively. The tube was then sealed and the mixture was heated to reflux at 40 °C overnight. TLC-analysis indicated full conversion of the starting material and the reaction was quenched with sat. aq. $NaHCO_3$ at 0 °C and diluted with DCM. The layers were separated and the aqueous layer was extracted with DCM (3 x). The combined organic layers were washed with H_2O (2 x), brine, dried over Na_2SO_4, filtered and concentrated *in vacuo*. The product was purified by silica gel column chromatography (pentane/EtOAc 50:1→20:1) affording compound **21** (208 mg, 0.396 mmol, 87%) as a clean oil. ^1H NMR (400 MHz, $CDCl_3$ at 293K) δ 7.71 (ddt, J = 13.7, 6.7, 1.6 Hz, 4H, CH Ar), 7.50 – 7.31 (m, 6H, CH Ar), 5.88 (s, 1H, H7), 5.25 – 5.05 (m, 2H, H8), 4.88 (s, 1H, CH*H* MOM), 4.58 – 4.40 (m, 1H, CH*H* MOM), 3.75 (brs, 2H, H3 and H6a), 3.63 (d, J = 13.2 Hz, 1H, H2a), 3.44 (dd, J = 8.3, 6.9 Hz, 1H, H4), 3.28 (s, 3H, CH_3 MOM), 2.85 (brs, 2H, H2b and H6b), 2.26 (qd, J = 8.6, 4.4 Hz, 1H, H5), 1.28 (s, 9H, $(CH_3)_3CSi$), 1.06 (s, 9H, $(CH_3)_3CO$). ^{13}C NMR (101 MHz, $CDCl_3$ at 293K) δ 154.6 (C=O), 137.5 (C7), 135.6 (4CH Ar), 134.1 (C_q Ar), 133.3 (C_q Ar), 129.9 (2CH Ar), 127.7 (4CH Ar), 117.1 (C8), 98.0 (CH_2 MOM), 82.5 (C4), 79.8 (C_q Boc), 73.0 (C3), 56.0 (CH_3 MOM), 48.4 (C2), 46.1 (C6), 45.2 (C5), 28.4 ($(\mathit{C}H_3)_3CSi$), 27.1 ($(\mathit{C}H_3)_3CO$), 19.3 ($(CH_3)_3\mathit{C}Si$) ppm. HRMS (ESI) m/z: $[M+Na]^+$ calc. for $C_{30}H_{43}NO_5SiNa$ 548.2803, found 548.2800.

Compound 22

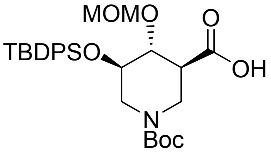

To a stirred solution of compound **21** (26 mg, 50 µmol) in CCl_4 (0.3 mL) and MeCN (0.3mL) was added a solution of $NaIO_4$ (53 mg, 0.25 mmol) and $RuCl_3{\cdot}3H_2O$ (0.1 M in H_2O, 50 µL, 5.0 µmol) in water (0.45 mL) at 0 °C. The reaction mixture was then stirred vigorously at rt for 3 h. After which, sat. aq. $Na_2S_2O_3$ was added to quench the reaction and the resulting mixture was stirred for another 15 min at rt. The layers were separated and the aqueous layer was extracted with EtOAc (3 x). The combined organic layers were washed with H_2O, brine, dried over Na_2SO_4, filtered and concentrated *in vacuo*. The product was purified by silica gel column chromatography (pentane/EtOAc 15:1→5:1, with 0.1% HOAc) affording compound **22** (17.5 mg, 32.0 µmol, 64%) as a clean oil. ^1H NMR (500 MHz, $CDCl_3$ at 293K) δ 7.74 – 7.66 (m, 4H, CH Ar), 7.48 – 7.34 (m, 6H, CH Ar), 4.67 (s, 1H, CH*H* MOM), 4.49 (s, 1H, CH*H* MOM), 3.99 (t, J = 6.3 Hz, 1H, H4), 3.84 – 3.64 (brs, 1H, H6a), 3.73 (q, J = 5.7 Hz, 1H, H3), 3.60 – 3.29 (brs, 2H, H6b and H2a), 3.22 (s, 3H, CH_3 MOM), 2.59 (brs, 1H, H2b), 1.33 (s, 9H, $(CH_3)_3CSi$), 1.05 (s, 9H, $(CH_3)_3CO$). ^{13}C NMR (126 MHz, $CDCl_3$ at 293K) δ 136.0 (4CH Ar), 130.0

(2CH Ar), 127.8 (4CH Ar), 97.4 (CH_2 MOM, assigned by HSQC), 78.8 (C4, assigned by HSQC), 70.9 (C3, assigned by HSQC), 56.0 (CH_3 MOM), 47.4 (C2, assigned by HSQC), 46.0 (C5, assigned by HSQC), 41.9 (C6), 28.4 ((CH$_3$)$_3$CSi), 27.1 ((CH$_3$)$_3$CO), 19.3 ((CH$_3$)$_3$$C$Si) ppm. HRMS (ESI) m/z: $[M+Na]^+$ calc. for $C_{29}H_{41}NO_7SiNa$ 566.2545, found 566.2544.

Compound 11

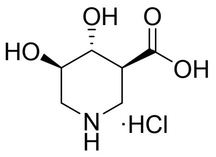

A solution of compound **22** (16 mg, 29 μmol) in dioxane (0.25 mL) in a microwave tube was added aqueous 3 M HCl (1.5 mL). The tube was then sealed and the mixture was stirred at 100 °C for 4 h until LC-MS indicated full deprotection. After cooling to rt, Et_2O (5 mL) was added and the mixture was stirred vigorously at rt for 5 min. The phases were then separated and the organic layer was taken out carefully. The remaining H_2O layer was washed two more times with Et_2O (2 x 5 mL) and concentrated. The residue was purified by silica gel column chromatography (isopropanol/H_2O/28-30% NH_4OH 12:1:1→10:2:1) to give chromatographically pure **11**. Milli-Q water (1 mL) and aqueous 1 M HCl (2 mL) were added to the residue, and the solution was evaporated to form a hydrochloride salt of **11**, which was further purified by a column of Sephadex G-25 with Milli-Q as eluent, affording title compound **11** (5.5 mg, 28 μmol, 95%) as a white solid after lyophilization. ^{1}H NMR (500 MHz, D_2O at 293K) δ 4.04 – 3.96 (m, 1H, H4), 3.90 – 3.82 (m, 1H, H3), 3.49 (dp, J = 12.9, 3.6, 2.7 Hz, 2H, H6a and H2a), 3.30 (tt, J = 10.8, 2.6 Hz, 1H, H6b), 3.08 – 2.99 (m, 1H, H2b), 2.81 (td, J = 7.9, 3.6 Hz, 1H, H5). ^{13}C NMR (126 MHz, D_2O at 293K) δ 174.3 (C=O), 70.2 (C4), 66.9 (C3), 45.7 (C2), 45.0 (C5), 42.5 (C6). HRMS (ESI) m/z: $[M+H]^+$ calc. for $C_6H_{12}NO_4$ 162.0761, found 162.0763.

Compound 23

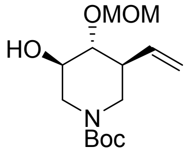

Compound **21** (187 mg, 0.35 mmol) was dissolved in dry THF (3.5 mL). TBAF (1 M in THF, 2.1 mL, 2.1 mmol) was added and the mixture was stirred at rt for 1.5 h. The reaction was quenched with sat. aq. NH_4Cl and concentrated *in vacuo* to remove THF. The resulting residue was diluted with DCM, washed with H_2O (2 x), brine, dried over Na_2SO_4, filtered and concentrated *in vacuo*. The product was purified by silica gel column chromatography (pentane/EtOAc 9:1→3:1) affording compound **23** (96 mg, 0.33 mmol, 94%) as a clean oil. ^{1}H NMR (500 MHz, $CDCl_3$ at 293K) δ 5.74 – 5.63 (m, 1H, H7), 5.19 (dd, J = 12.1, 1.3 Hz, 1H, H8a), 5.19 – 5.14 (m, 1H, H8b), 4.77 (dd, J = 6.9, 0.9 Hz, 1H, CHH MOM), 4.65 (dd, J = 7.0, 1.1 Hz, 1H, CHH MOM), 4.53 – 4.17 (m, 2H, H6a and H2a), 3.50 – 3.40 (H3), 3.46 (s, 3H, CH_3 MOM), 3.08 (dd, J = 10.2, 8.3 Hz, 1H, H4), 2.64 – 2.47 (m, 2H, H6b and H2b), 2.35 – 2.23 (m, 1H, H5), 1.46 (d, J = 1.3 Hz, 9H, $(CH_3)_3C$). ^{13}C NMR (126 MHz, $CDCl_3$ at 293K) δ 154.6 (C=O), 135.9 (C7), 117.9 (C8), 98.5 (CH_2 MOM), 89.2 (C4), 80.2 ((CH$_3$)$_3$$C$), 70.0 (C3), 56.0 ($CH_3$ MOM), 48.5 (C2, assigned by HSQC), 46.3 (C6, assigned by HSQC), 44.9 (C5, assigned by HSQC), 28.5 ((CH$_3$)$_3$C). HRMS (ESI) m/z: $[M+Na]^+$ calc. for $C_{14}H_{25}NO_5Na$ 310.1625, found 310.1623.

Compound 24

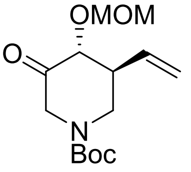

To a solution of compound **23** (96 mg, 0.33 mmol) in dry DCM (5 mL) at 0 °C was added Dess-Martin periodinane (283 mg, 0.66 mmol). The mixture was stirred at rt for 1 h until TLC-analysis indicated total consumption of the starting material. A mixture of sat. aq. $Na_2S_2O_3$ (3 mL) and sat. aq. $NaHCO_3$ (3 mL) was added to quench the reaction and the mixture was stirred at rt for another 15 min until the white emulsion became clear solution. The layers were separated and the aqueous layer was extracted with DCM (2 x). The combined organic layers were washed with H_2O, brine, dried over Na_2SO_4, filtered and concentrated *in vacuo*. The product was purified by silica gel column chromatography (pentane/EtOAc 9:1→3:1) affording compound **24** (88 mg, 0.31 mmol, 93%) as a clean oil. ^1H NMR (500 MHz, $CDCl_3$ at 293K) δ 5.77 (ddd, J = 17.6, 10.4, 7.6 Hz, 1H, H7), 5.30 – 5.20 (m, 2H, H8), 4.79 (dd, J = 7.1, 1.0 Hz, 1H, CH*H* MOM), 4.67 (dd, J = 7.0, 1.0 Hz, 1H, CH*H* MOM), 4.28 (dd, J = 16.7, 1.5 Hz, 1H, H2a), 4.10 (d, J = 10.2 Hz, 1H, H4), 4.07 – 3.99 (brs, 1H, H6a), 3.84 (brs, 1H, H2b), 3.39 (d, J = 1.0 Hz, 3H, CH_3 MOM), 3.37 – 3.17 (m, 1H, H6b), 2.71 (qd, J = 9.7, 5.0 Hz, 1H, H5), 1.46 (d, J = 1.0 Hz, 9H, $(CH_3)_3C$). ^{13}C NMR (126 MHz, $CDCl_3$ at 293K) δ 202.5 (C3), 154.3 (C=O Boc), 135.3 (C7), 118.5 (C8), 96.5 (CH_2 MOM), 81.1 ($(CH_3)_3$*C*), 80.0 (C4), 56.3 (CH_3 MOM), 54.2 (C2, assigned by HSQC), 46.6 (C5), 46.0 (C6, assigned by HSQC), 28.4 ((*C*H_3)$_3$C) ppm. HRMS (ESI) m/z: $[M+Na]^+$ calc. for $C_{14}H_{23}NO_5Na$ 308.1468, found 308.1465.

Compound 25

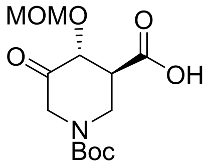

To a solution of compound **24** (35 mg, 0.12 mmol) in a mixture of CCl_4 (1.4 mL) and MeCN (1.4 mL) was added a solution of $NaIO_4$ (130 mg, 0.60 mmol, 5.0 eq.) and $RuCl_3 \cdot 3H_2O$ (2.5 mg, 0.012 mmol, 0.1 eq.) in water (2.1 mL) at 0 °C. The reaction mixture was then stirred vigorously at rt for 2 h until TLC and LC-MS analysis indicated complete conversion. The phases were separated and the aqueous phase was extracted with EtOAc (3 x 15 mL). To the combined organic extracts was added isopropanol (0.2 mL) and the mixture was stirred at rt for an additional 1 hour. The mixture was then washed with H_2O (15 mL), brine (15 mL), dried over Na_2SO_4, filtered and concentrated *in vacuo*. The product was purified by silica gel column chromatography (DCM/MeOH 100:0→100:4) affording compound **25** (31 mg, 0.10 mmol, 83%) as a clean oil. ^1H NMR (500 MHz, $CDCl_3$ at 333K) δ 4.79 (d, J = 6.7 Hz, 1H, CH*H* MOM), 4.71 (d, J = 6.7 Hz, 1H, CH*H* MOM), 4.49 (d, J = 8.5 Hz, 1H, H4), 4.09 (d, J = 16.8 Hz, 1H, H2a), 3.99 (d, J = 16.7 Hz, 2H, H2b and H6a), 3.79 (dd, J = 13.7, 7.4 Hz, 1H, H6b), 3.39 (s, 3H, CH_3 MOM), 3.05 – 2.97 (m, 1H, H5), 1.46 (s, 9H, $(CH_3)_3C$). ^{13}C NMR (126 MHz, $CDCl_3$ at 333K) δ 174.0 (COOH), 154.4 (C=O Boc), 96.9 (CH_2 MOM), 81.8 ($(CH_3)_3$*C*), 77.2 (C4), 56.4 (CH_3 MOM), 53.4 (C2, assigned by HSQC), 48.1 (C5), 43.4 (C6), 28.4 ((*C*H_3)$_3$C) ppm. HRMS (ESI) m/z: $[M+Na]^+$ calc. for $C_{13}H_{21}NO_7Na$ 326.1210, found 326.1210; $[M+H_2O+Na]^+$ calc. for $C_{13}H_{23}NO_8Na$ 344.1316, found 344.1316.

Compound 9

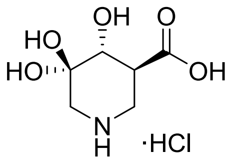

Compound **25** (24 mg, 79 µmol) was dissolved in 4 M dioxane/H_2O (1.5 mL) and stirred at rt for 5 h. After which, water (1.0 mL) and Et_2O (~8.0 mL) were added. The mixture was stirred vigorously for a while and stood for stratification. The Et_2O layer was taken out carefully and the remaining water layer was washed two more times with Et_2O (2 x 8.0 mL) and concentrated *in vacuo*. The resulting dry residue was re-dissolved in MeOH (1.0 mL) and Et_2O (~8.0 mL) was added slowly under vigorous stirring. Upon addition of Et_2O, a lot of light-yellow solid appeared. After stirring vigorously for a while, the mixture was stood for solid precipitation and the Et_2O supernatant was taken out carefully. The solid was washed repeatedly with Et_2O (3 x 6.0 mL), re-dissolved in Milli-Q water (~1.5 mL) and filtered over a Whatman filter paper. After lyophilization, the target product (10.8 mg, 55 µmol, 70%) was obtained as a light-yellow solid (HCl salt). ^1H NMR after lyophilization (500 MHz, D_2O at 293K) [hydrate form] δ 4.07 (d, J = 7.5 Hz, 1H, H4), 3.48 (ddd, J = 13.2, 4.5, 1.1 Hz, 1H, H6a equatorial), 3.38 (dd, J = 13.2, 8.0 Hz, 1H, H6b axial), 3.35 (dd, J = 12.9, 1.0 Hz, 1H, H2a), 3.14 (d, J = 12.9 Hz, 1H, H2b), 3.04 (td, J = 8.0, 7.5, 4.5 Hz, 1H, H5). ^{13}C NMR after lyophilization (126 MHz, D_2O at 293K) [hydrate form] δ 173.6 (COOH), 90.7 (C3 hydrate), 71.1 (C4), 49.0 (C2), 44.4 (C5), 41.9 (C6) ppm. HRMS (ESI) m/z: $[M_{ketone}+H]^+$ calc. for $C_6H_{10}NO_4$ 160.06043, found 160.06059; $[M_{hydrate}+H]^+$ calc. for $C_6H_{12}NO_5$ 178.07100, found 178.07119.

NMR of siastatin B

The potential formation of siastatin B breakdown products was evaluated with ^1H NMR. Siastatin B (2 mg) was dissolved in 550 µL phosphate buffer (50 mM, 300 mM NaCl in D_2O, pH 5.0) in all NMR experiments. NMR spectra were taken before and after prolonged incubations. One sample was left at 20 °C for 18 hours, another sample was incubated at 22 °C with 1.2 µM of recombinant human heparanase. Recombinant human heparanase was freeze dried before use to remove non-deuterated H_2O. ^1H NMR spectra were recorded with the use of a Bruker AV 500 MHz spectrometer. Presat settings were used during the measurements to suppress the residual H_2O/HDO peak and obtain clear spectra.

Mass spectroscopy of siastatin B

LC-MS–grade water and acetonitrile were purchased from Biosolve (Valkenswaard, The Netherlands). Siastatin B samples were dissolved in water to a final concentration of 1 µM. 10 µL of the sample was injected for the HPLC–MS analysis.

*HPLC-MS :*Measurements were performed by reverse-phase liquid chromatography using a Waters UPLC-Xevo-TQS micro and Acquity UPLC BEH Amide column (100 × 2.1 mm,1.7 µm, Waters, USA), maintained at 23°C using the following eluents: phase A water and phase B acetonitrile. A mobile-phase gradient was used during a 40 min run: 0.00 min 90% B; 20 min 50% B; 38 min 90% B; 40 min 90% B. The flow rate was 0.25mL/min. The eluent was diverted to waste between 0.00 and 1.00 min to keep the source free of contaminants; data were collected between 1.00 and 40 min. The autosampler temperature was set at 10°C and the injection volume was 10 µL. Mass spectrometry detection in positive mode using an electrospray ionization (ESI) source was carried out with a Xevo TQS micro instrument. The conditions were as follows: the temperatures of source and desolvation

were 110 and 400°C, respectively, with the flow rate of desolvation gas at 450 L/h and cone gas 30 L/h. A selected ion monitoring (SIR) was included to detect the following masses: 159-161, 177-179, 217-219. The capillary voltage was set to 3.0 kV and the cone voltage was set to 10 V. The data were collected and analyzed by MassLynx ™ NT 4.1 software with QuanLynx ™ program (Waters, Milford, MA, USA). Product ion MS spectra of [M + H] + of the detected compounds.

To monitor the purification of siastatin B we also performed ultra-high performance liquid chromatography before and after purification. These measurements were performed by reverse-phase liquid chromatography using an Agilent 6475 Triple Quadruple LC/MS System equipped with a Nucleodur 100-5 NH2-RP 5um 4.6 x 150 mm column (Macherey-Nagel, Germany), maintained at 40 °C using the following eluents: phase A water, phase B acetonitrile and phase C is 1 % Acetic acid (UPLC/MS grade). A mobile-phase gradient was used for 25 min from 90 % B to 50 % B and 10 % C. An equilibration step of 7.5 min 90 % B and 10 % C was included before the gradient while a cleaning step of another 7.5 min of 50 % B and 10 % C followed after the gradient. The flow rate was maintained at1 mL/min. The eluent was only diverted to MS between 7.5 and 20 min to keep the source free of contaminants. The injection volume was 10 µL. Mass spectrometry detection was performed in negative mode using an electrospray ionization (AJS ESI) source. The conditions were as follows: the gas and sheath gas temperature were respectively 325 and 400 degrees; the capillary voltage was set to 4.0 kV and the fragmentor to 70V; the gas flow was 10 L/min, nebulizing gas 20 psi and sheath gas 11 L/min. A scan mode from m/z= 115 to 230 with a scan speed of 500ms was used. After the run a selection of different m/z-values were used to detect the different masses: 176.0 (contaminant), 217.0 (Siastatin-b) and 158.0 (contaminant). Product ion MS spectra of [M-H] - of the detected compounds were obtained.

HR-MS: High-resolution mass spectrometry (HRMS) analysis was performed with a LTQ Orbitrap mass spectrometer (Thermo Finnigan), equipped with an electronspray ion source in positive mode and negative mode (source voltage 3.5 kV, sheath gas flow 10 mL/min, capillary temperature 250 °C) with resolution R = 60000 at m/z 400 (mass range m/z = 130 – 230) The high-resolution mass spectrometer was calibrated prior to measurements with a calibration mixture (Thermo Finnigan).

<u>Purification of siastatin B</u>

Crude siastain B was purified by reversed-phase HPLC chromatography using a linear gradient 70-40 % B (13 min) with eluent A (0.2% AcOH in MilliQ) and eluent B (CH_3CN) on an Agilent Technologies 1200 series instrument equipped with a semi-preparative column (Nucleodur 100-5 NH2-RP, 250x10 mm column, Macherey-Nagel, Germany). Lyophilization afforded the pure compound.

<u>Recombinant protein production and purification</u>

HPSE – Human HPSE was expressed and purified according to previously reported procedures[5].

AcGH79 – AcGH79 was expressed and purified according to previously reported procedures[6].

BpHep – The coding sequence of BpHep was cloned into the pET28a vector (Novagen), behind an N-terminal 6xHis tag and thrombin cleavage site and used to transform *E. coli* strain BL21 Gold (DE3) (Agilent). Transformants were grown in TB media supplemented with 50 µg/mL kanamycin at 37 °C until cultures reached an OD600 of 0.8-1.0, whereupon expression was induced by the addition of 0.5 mM isopropyl β-D-1-thiogalactopyranoside (IPTG; Sigma). Induced cultures were grown at 16 °C overnight, then harvested by 4,000 g centrifugation at 4 °C for 15 min.

Harvested cells were resuspended in ~50 mL Histrap buffer (20 mM Tris pH 8.0, 500 mM NaCl, 20 mM imidazole, 1 mM DTT), supplemented with DNAse I (Sigma; bovine pancrease), and cOmplete™ EDTA protease inhibitors (Roche). Cells were lysed using a cell disruptor (Constant systems) at 40 kPSI operating pressure, and lysate clarified by centrifugation at 40,000 g at 4 °C for 30 min. Clarified supernatant was loaded onto a 5 mL HisTrap FF crude column (Cytiva) pre-equilibrated with HisTrap

buffer A, washed with 10 column volumes (CV) of HisTrap buffer A, before eluting with HisTrap buffer B (20 mM Tris pH 8.0, 500 mM NaCl, 1000 mM imidazole, 1 mM DTT) over a 20 CV linear gradient. BpHep containing fractions were pooled, and buffer exchanged into 20 mM HEPES pH 7.4, 100 mM NaCl by at least 3 rounds of sequential concentration/dilution using a 30 kDa molecular weight cut-off (MWCO) Vivaspin centrifugal concentrator (Cytiva). Buffer exchanged BpHep was digested overnight at ambient temperature with thrombin (Sigma; bovine plasma) at 1:100 mass ratio thrombin:BpHep.

Digested BpHep was rerun over a 5 mL HisTrap FF crude column pre-equilibrated with HisTrap buffer A, which was further washed with 3 CV of Histrap buffer A. Combined flowthrough and wash fractions were concentrated to ~2 mL volume using a 30 kDa MWCO Vivaspin centrifugal concentrator, then loaded onto a Superdex S75 16/600 pg size exclusion chromatography (SEC) column (Cytiva) pre-equilibrated in SEC buffer (20 mM HEPES pH 7.4, 200 mM NaCl, 1 mM DTT). BpHep containing fractions were pooled and concentrated using a 30 kDa MWCO Vivaspin centrifugal concentrator to a final concentration of ~20 mg/ml. Purified protein was flash frozen in liquid nitrogen (LN2) and stored at –80 °C for use in further experiments.

EcGUSB - The coding sequence of EcGUSB was cloned into the pET28a vector, behind an N-terminal 6xHis tag, and used to transform *E. coli* strain BL21 Gold (DE3). Transformants were grown in TB media supplemented with 50 μg/mL kanamycin at 37 °C until cultures reached an OD600 of 0.8-1.0, whereupon gene expression was induced by the addition of 0.5 mM IPTG. Induced cultures were grown at 16 °C overnight, then harvested by 4,000 g centrifugation at 4 °C for 15 min.

Harvested cells were resuspended in ~50 mL Histrap buffer (20 mM Tris pH 8.0, 500 mM NaCl, 20 mM imidazole, 1 mM DTT), supplemented with DNAse I, and cOmplete™ EDTA protease inhibitors. Cells were lysed using a cell disruptor at 40 kPSI operating pressure, then lysate clarified by centrifugation at 40,000 g at 4 °C for 30 min. Clarified supernatant was loaded onto a 5 mL HisTrap FF crude column pre-equilibrated with HisTrap buffer A, washed with 10 CV of HisTrap buffer A, before eluting with HisTrap buffer B (20 mM Tris pH 8.0, 500 mM NaCl, 1000 mM imidazole, 1 mM DTT) over a 20 CV linear gradient. BpHep containing fractions were pooled, and concentrated using a 30 kDa MWCO Vivaspin centrifugal concentrator to a volume of ~ 2 mL. Concentrated protein was loaded onto a Superdex S200 16/600 pg SEC column pre-equilibrated in SEC buffer (20 mM HEPES pH 7.4, 200 mM NaCl, 1 mM DTT). EcGUSB containing fractions were pooled and concentrated using a 30 kDa MWCO Vivaspin centrifugal concentrator to a final concentration of ~24.5 mg/ml. Purified protein was flash frozen in LN2 and stored at -80 °C for use in further experiments.

Crystallization :

AcGH79 – Well diffracting crystals of AcGH79 were obtained by the sitting-drop vapor-diffusion method at 20 °C using a well solution containing 0.5 M ammonium sulfate, 1 M lithium sulfate, 0.1 M trisodium citrate, and a protein to well solution ratio of 500 nl: 500 nl. Crystals typically appeared after 1 week.

Inhibitor ligand complexes were obtained by transferring AcGH79 crystals to drops containing 2 M lithium sulfate and 1 mM inhibitor. Crystals were incubated with ligand for ~0.5-1h, then directly harvested and flash-cooled in LN2 for data collection.

BpHEP – Well diffracting crystals of BpHep were obtained by the sitting-drop vapor-diffusion method at 20 °C using a well solution containing 0.1 M sodium citrate pH 5.0, 14% (w/v) PEG 6000, and a protein to well solution ratio of 300 nl: 500nl. Crystals typically appeared after 3 days.

Inhibitor ligand complexes were obtained by transferring BpHep crystals to drops of well solution supplemented with 25 % (v/v) ethylene glycol and 1–5 mM inhibitor. Crystals were incubated with ligand for ~2–4 h, then directly harvested and flash-cooled in LN2 for data collection.

EcGusB – Initial crystals of EcGUSB were obtained by the sitting-drop vapor-diffusion method at 20 °C using a well solution containing 0.1 M Bis-Tris propane pH 7.5, 20% (w/v) PEG 3350, 0.2 M $NaNO_3$. These initial crystals were used to prepare a microseed stock using Seed Beads (Hampton), then used to seed well diffracting crystals of EcGUSB in the same well conditions, at a protein to seed to well solution ratio of 500 nl: 200 nl: 1000 nL.

Inhibitor ligand complexes were obtained by transferring EcGUSB crystals to drops of well solution supplemented with 25 % (v/v) ethylene glycol and 1–5 mM inhibitor. Crystals were incubated with ligand for ~2–4 h, then directly harvested and flash-cooled in LN2 for data collection.

HPSE – Well diffracting crystals of HPSE were obtained by the sitting-drop vapor-diffusion method at 20 °C using a well solution containing 0.1 M MES pH 5.5, 0.1 M $MgCl_2$, 17 % (w/v) PEG 3350, and a protein to well solution ratio of 200 nl: 500 nl. Crystals typically appeared after 1 week.

Inhibitor ligand complexes were obtained by transferring HPSE crystals to drops of well solution supplemented with 25 % (v/v) ethylene glycol and 1 mM inhibitor. Crystals were incubated with ligand for ~0.5-1h, then directly harvested and flash-cooled in LN2 for data collection.

X-ray data collection and structure solution :

Xray diffraction data were collected at 100 K at beamlines i03, i04 and i04-1 of the Diamond Light Source UK. Reflections were autoprocessed using the XIA2 pipeline[7]. Complexes were solved by directly refining against their unliganded structures where solved using molecular replacement with PHASER4 (search model PDB accessions 5E98 (HPSE), 3VNY (AcGH79), 3K46 (EcGUSB)). Solved structures were iteratively improved by rounds of manual model building and maximum-likelihood refinement using COOT[8] and REFMAC5[9] respectively. Ligand coordinates were built using jLigand[10]. Diagrams were generated using PyMOL (Delano Scientific).

cABPP Labelling experiments:

ABP labelling reactions were carried out in McIlvaine buffer (pH 5.0), 300 mM NaCl. Labeling reactions were initiated by the addition of ABP **VB-A69** to a final concentration of 100 nM, before incubation for 30 min at 37°C with shaking. Following labeling, samples were denatured by boiling with Laemmli buffer for 5 min and resolved by SDS-PAGE. Gels were scanned using Typhoon-5 laser-scanner platform (Cytiva), using the λ_{EX} 635 nm laser and 670BP30 emission filter.

For competitive ABPP (cABPP) experiments, platelet lysates were preincubated with inhibitor (or buffer only control) in McIlvaine buffer (pH 5.0), 300 mM NaCl for 1 h at 37°C with shaking. The activity based probe **VB-A69 (described by Borlandelli et. al.[11])** was then added to a final concentration of 100 nM, and the reaction was subsequently incubated for 30 min at 37°C with shaking at 800 rpm. Following labeling, samples were denatured by boiling with Laemmli buffer for 5 min and resolved by SDS-PAGE. Gels were scanned using Typhoon-5 laser-scanner platform (Cytiva), using the λ_{EX} 635 nm laser and 670BP30 emission filter. For quantitative measurements, fluorescently labeled band intensities were calculated using Imagequant (Cytiva), and normalized to the corresponding band in the inhibitor free control lane. IC_{50} concentrations were determined by fitting data to the Hill equation:

$$A = A_{max}\left(\frac{[I]^n}{k^n + [I]^n}\right)$$

All kinetic parameters were fit with the software program Origin 2019.

Enzyme Kinetics

Initial rates were determined for AcGH79 and EcGusB hydrolysis of 4-methylumbelliferyl glucuronide in the presence of varying concentrations either commercial or HPLC purified siastatin B to determine inhibitory IC_{50} concentrations. Reactions contained 20 mM NaCl, 1 % BSA, 60 mM McIllvane Buffer pH 5.0 and 500 μM 4-methylumbelliferyl glucuronide and 0 – 400 μM siastatin B. Assays were initiated by the addition of enzyme to a final concentration of 2 nM. Fluorescence was monitored using a Clariostar plate reader using an excitation wavelength of 365 nm and emission wavelength of 450 nm. Assays were performed in quadruplicate and the variation of initial rates with inhibitor concentration were fit to the Hill equation: $A = A_{max}(\frac{[I]^n}{k^n + [I]^n})$ with the software program Origin 2019.

To determine inhibition constant (*Ki*) values, Michaelis-Menten parameters were determined for AcGH79 and EcGusB hydrolysis of 4-methylumbelliferyl glucuronide in reactions containing one of the inhibitors **8**, **9**, **10** or **11**. Assays were initiated by the addition of enzyme to the assay mix containing 0-1 mM 4-methylumbelliferyl glucuronide, 20 mM NaCl, 0.1 % BSA, 60 mM McIllvane Buffer pH 5.0. Fluorescence was monitored as above. Michaelis constants were determined for at least 3 different inhibitor concentrations for each different inhibitor. Inhibition constants were calculated according to a competitive inhibition model for both inhibitors using the equation : $K_{M_{app}} = K_M(1 + {[I]}/{K_i})$. All kinetic parameters were fit with the software program Origin 2019.

References:

(1) Ichikawa, Y. I., Y.,. An extremely potent inhibitor for β-galactosidase. *Tetrahedron Letters* **1995**, *36*, 4585-4586. DOI: 10.1016/0040-4039(95)00870-I.
(2) Jiang, J.; Kallemeijn, W. W.; Wright, D. W.; van den Nieuwendijk, A.; Rohde, V. C.; Folch, E. C.; van den Elst, H.; Florea, B. I.; Scheij, S.; Donker-Koopman, W. E.; et al. In vitro and in vivo comparative and competitive activity-based protein profiling of GH29 alpha-L-fucosidases. *Chemical Science* **2015**, *6* (5), 2782-false. DOI: 10.1039/c4sc03739a.
(3) Griengl, H. K., N.; Pöchlauer, P.; Schmidt, M.; Shi, N.; A. Zabelinskaja-Mackova, A.,. Enzyme Catalysed Formation of (S)-Cyanohydrins Derived from Aldehydes and Ketones in a Biphasic Solvent System. *Tetrahedron* **1998**, *54*, 14477-14486. DOI: 10.1016/S0040-4020(98)00901-6. Jiang, J.; Kallemeijn, W. W.; Wright, D. W.; van den Nieuwendijk, A. M. C. H.; Rohde, V. C.; Folch, E. C.; van den Elst, H.; Florea, B. I.; Scheij, S.; Donker-Koopman, W. E.; et al. In vitro and in vivo comparative and competitive activity-based protein profiling of GH29 α-l-fucosidases. *Chemical Science* **2015**, *6* (5), 2782-2789. DOI: 10.1039/c4sc03739a.
(4) Ouchi, H. M., Y.; Takahata, H.,. A New Route to Diverse 1-Azasugars from *N*-Boc-5-hydroxy-3-piperidene as a Common Building Block. *The Journal of Organic Chemistry* **2005**, *70*, 5207-5214. DOI: 10.1021/jo050519j.
(5) Wu, L.; Viola, C. M.; Brzozowski, A. M.; Davies, G. J. Structural characterization of human heparanase reveals insights into substrate recognition. *Nat Struct Mol Biol* **2015**, *22* (12), 1016-1022. DOI: 10.1038/nsmb.3136.
(6) Wu, L.; Jiang, J. B.; Jin, Y.; Kallemeijn, W. W.; Kuo, C. L.; Artola, M.; Dai, W.; van Elk, C.; van Eijk, M.; van der Marel, G. A.; et al. Activity-based probes for functional interrogation of retaining beta-glucuronidases. *Nat Chem Biol* **2017**, *13* (8), 867-+. DOI: 10.1038/nchembio.2395.
(7) Winter, G. xia2: an expert system for macromolecular crystallography data reduction. *Journal of Applied Crystallography* **2009**, *43* (1), 186-190. DOI: 10.1107/s0021889809045701.
(8) Emsley, P.; Lohkamp, B.; Scott, W. G.; Cowtan, K. Features and development of Coot. *Acta Crystallographica Section D Biological Crystallography* **2010**, *66* (4), 486-501. DOI: 10.1107/s0907444910007493.
(9) Murshudov, G. N.; Skubák, P.; Lebedev, A. A.; Pannu, N. S.; Steiner, R. A.; Nicholls, R. A.; Winn, M. D.; Long, F.; Vagin, A. A. REFMAC5 for the refinement of macromolecular crystal structures. *Acta Crystallographica Section D Biological Crystallography* **2011**, *67* (4), 355-367. DOI: 10.1107/s0907444911001314.

(10) Lebedev, A. A.; Young, P.; Isupov, M. N.; Moroz, O. V.; Vagin, A. A.; Murshudov, G. N. JLigand: a graphical tool for the CCP4 template-restraint library. *Acta Crystallographica Section D Biological Crystallography* **2012**, *68* (4), 431-440. DOI: 10.1107/s090744491200251x.
(11) Borlandelli, V.; Armstrong, Z.; Nin-Hill, A.; Codée, J. D. C.; Raich, L.; Artola, M.; Rovira, C.; Davies, G. J.; Overkleeft, H. S. 4-O-Substituted Glucuronic Cyclophellitols are Selective Mechanism-Based Heparanase Inhibitors. *ChemMedChem* **2023**, *18* (4). DOI: 10.1002/cmdc.202200580.

NMR spectra

^{1}H and ^{13}C of **S2** in $CDCl_3$ at 333 K

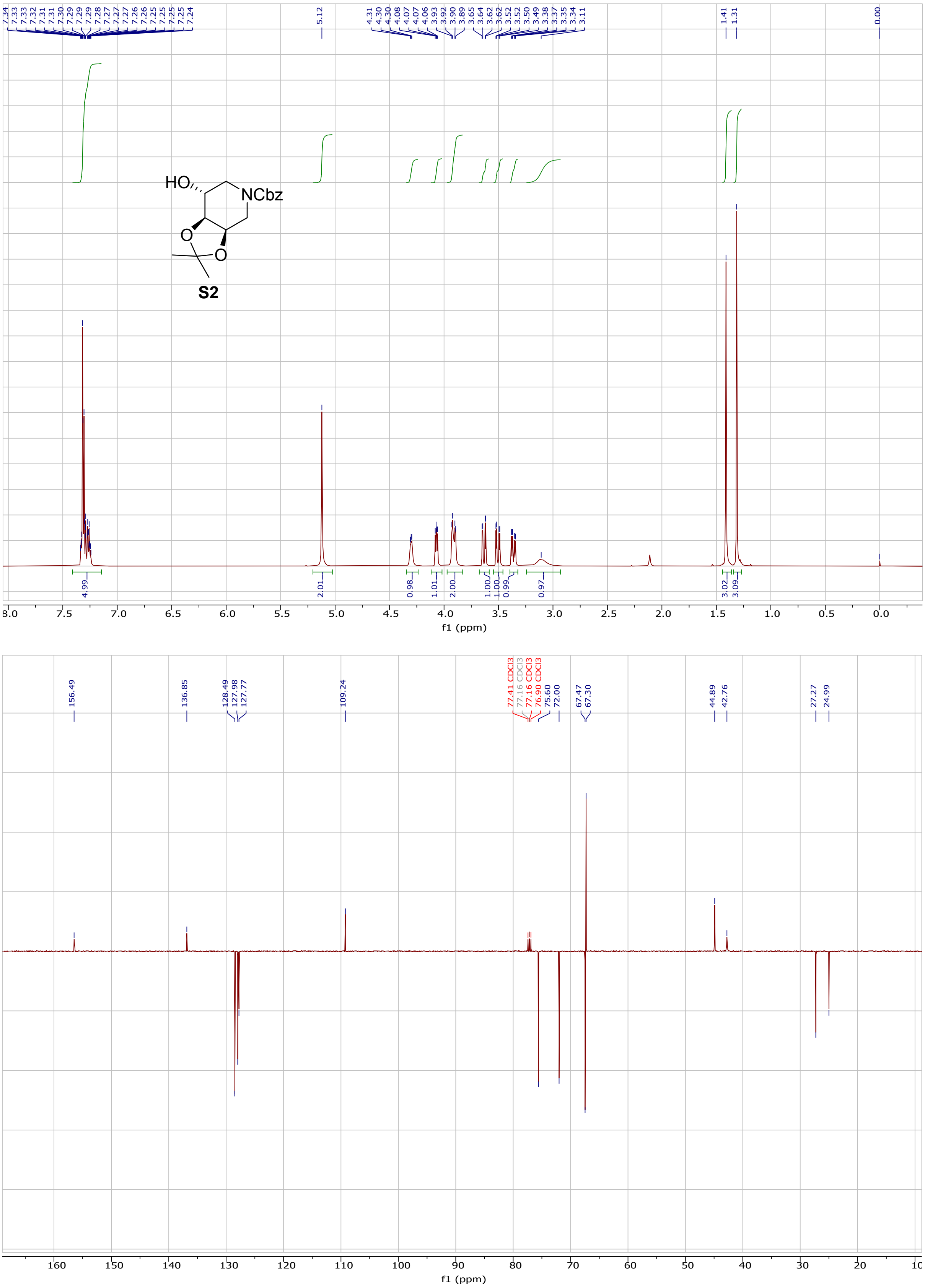

^{1}H and ^{13}C of **S3** in $CDCl_3$ at 333 K

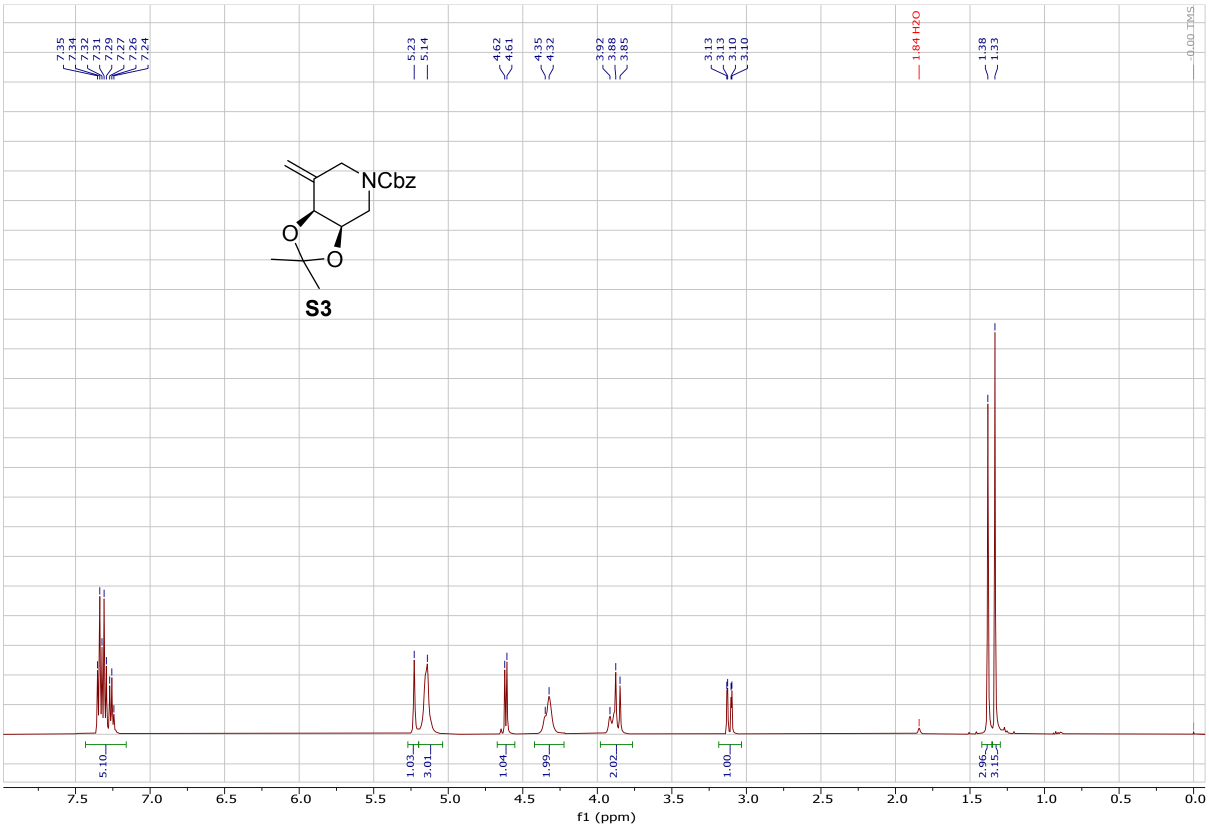

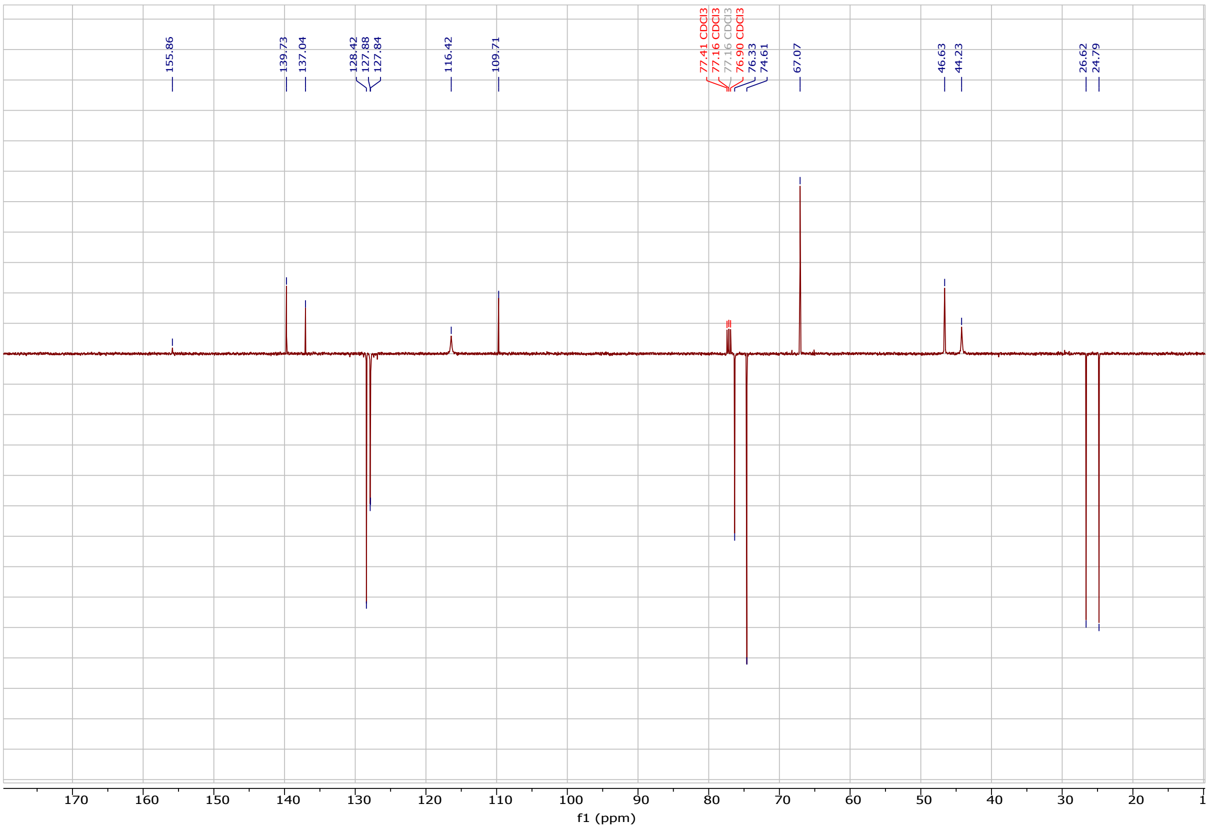

^{1}H and ^{13}C of **12** in $CDCl_3$ at 333 K

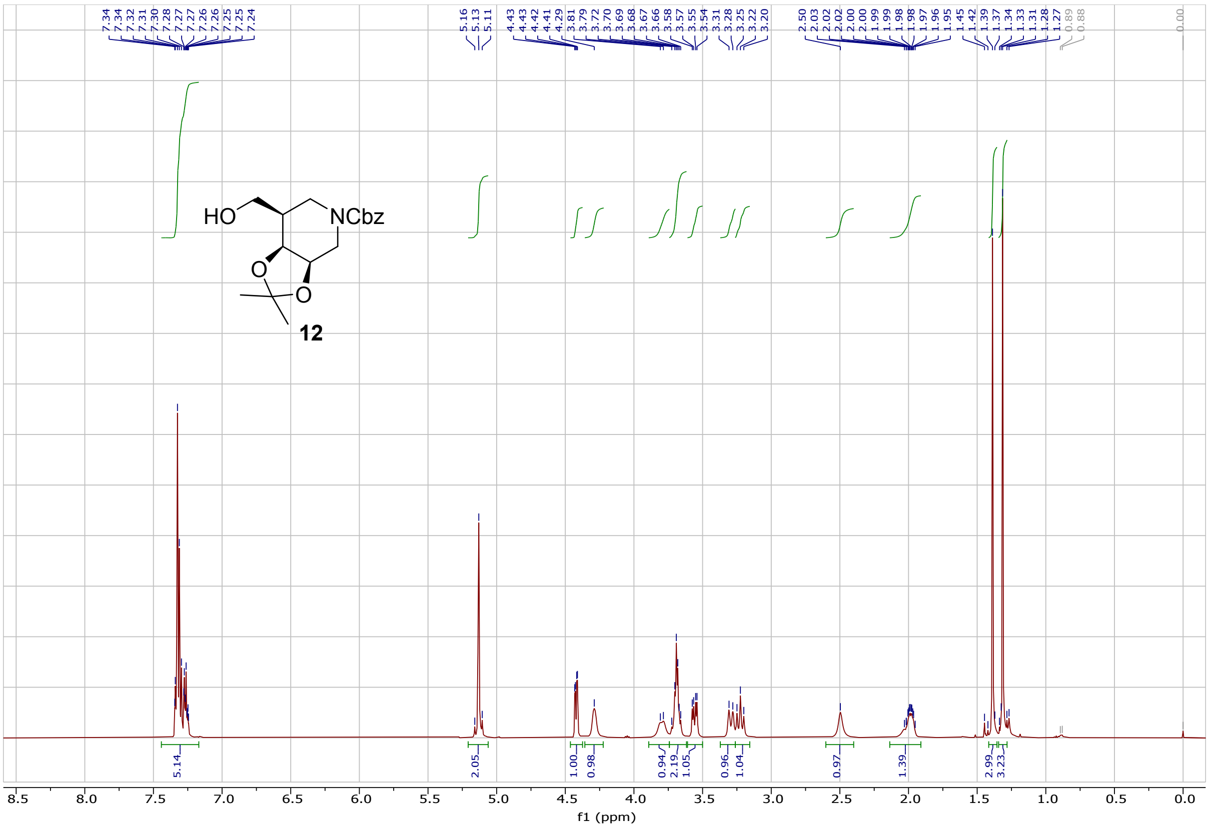

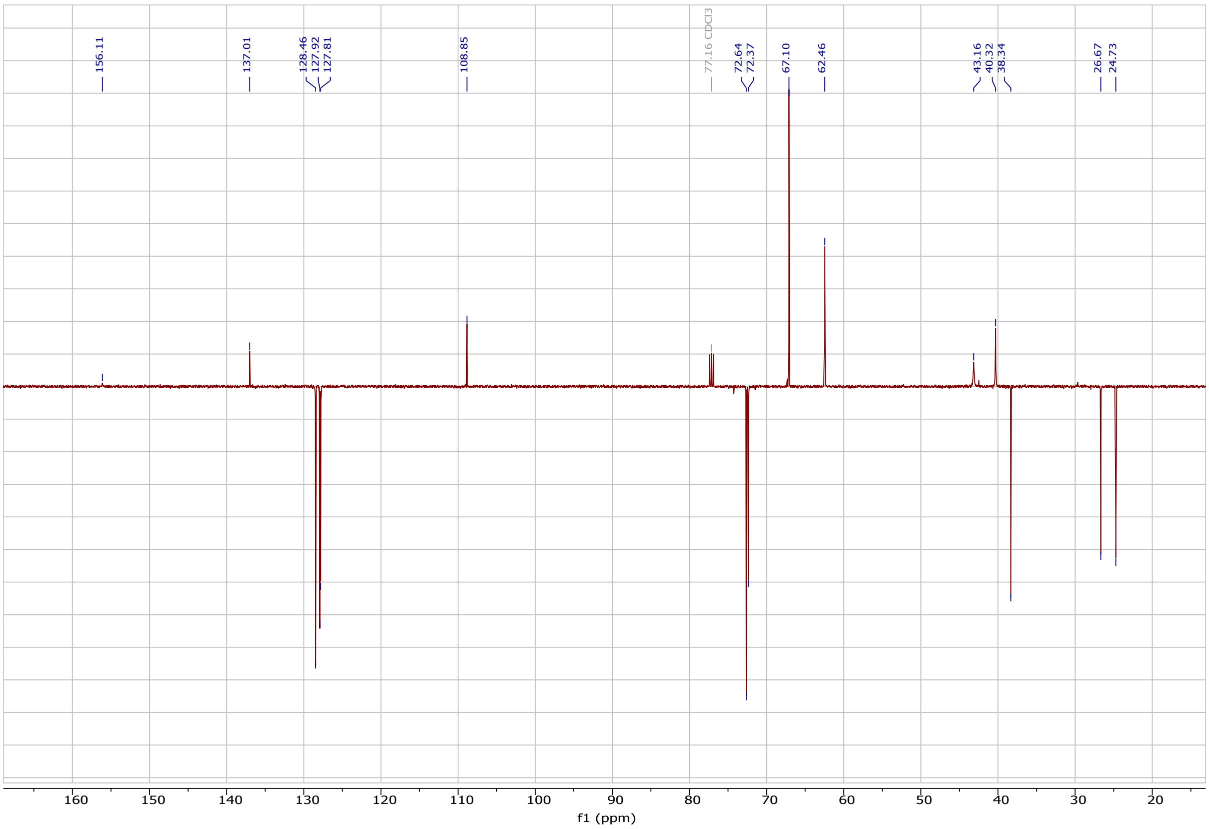

^{1}H and ^{13}C of **13** in $CDCl_3$ at 333 K

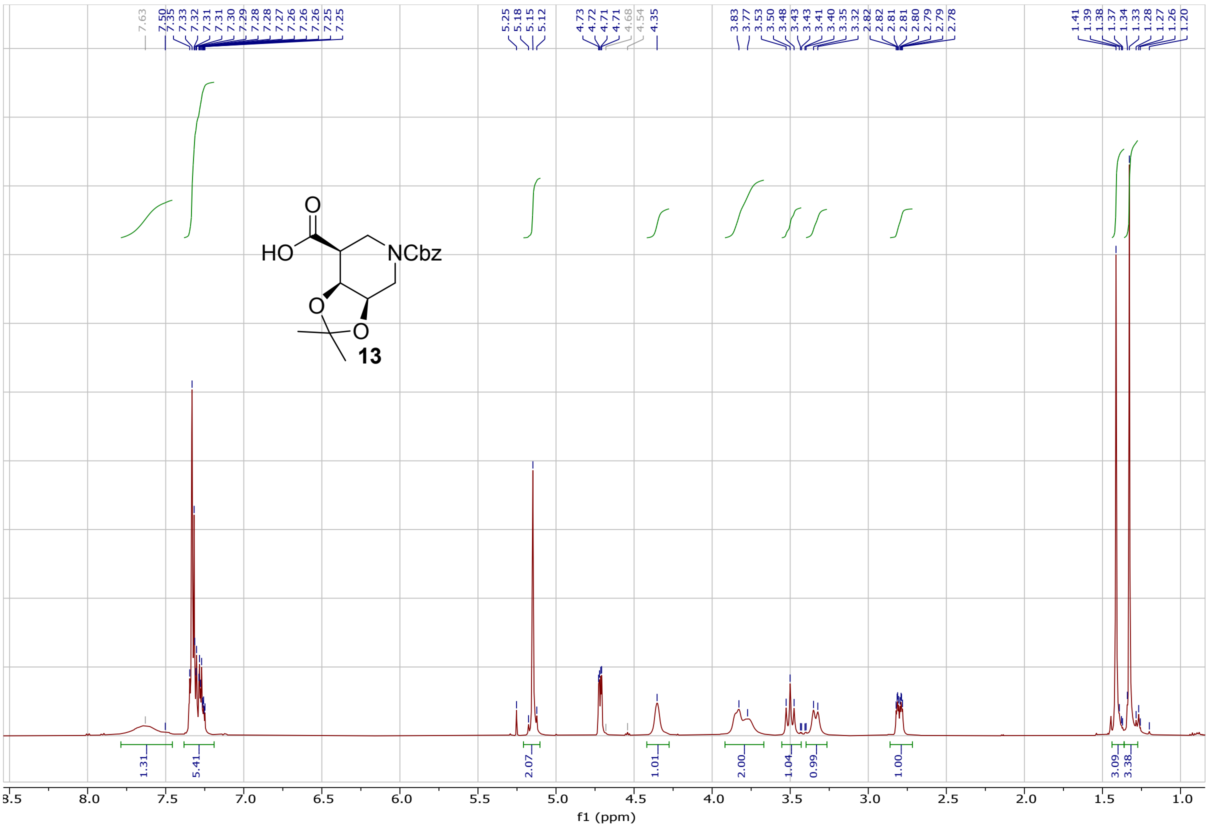

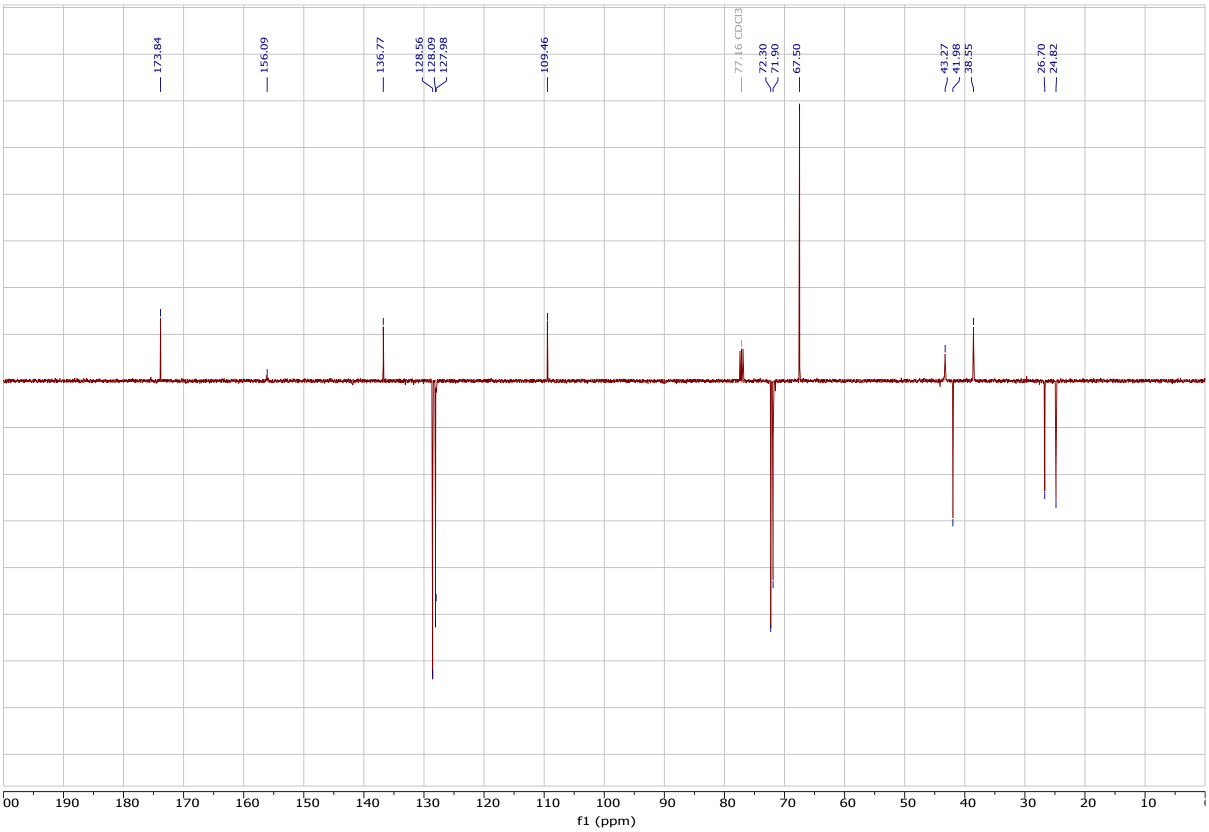

^{1}H and ^{13}C of **10** in D_2O at 293 K

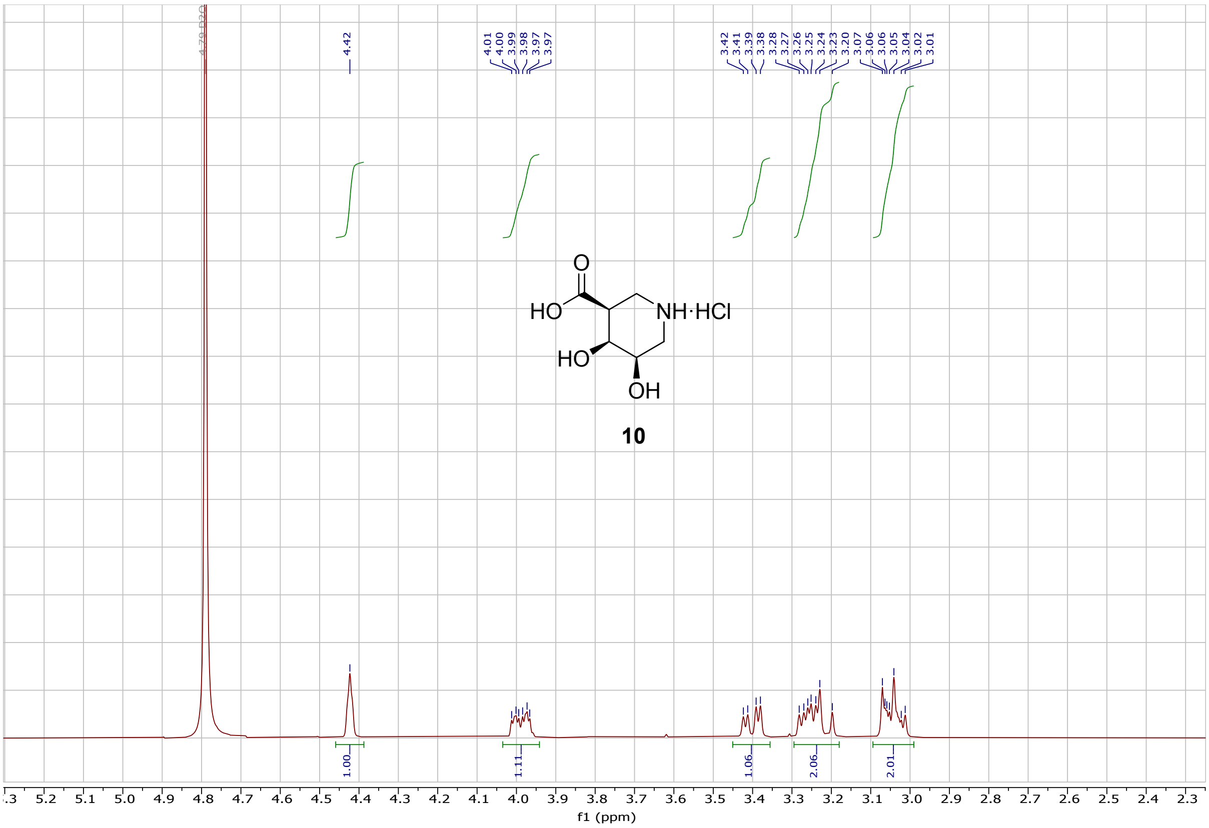

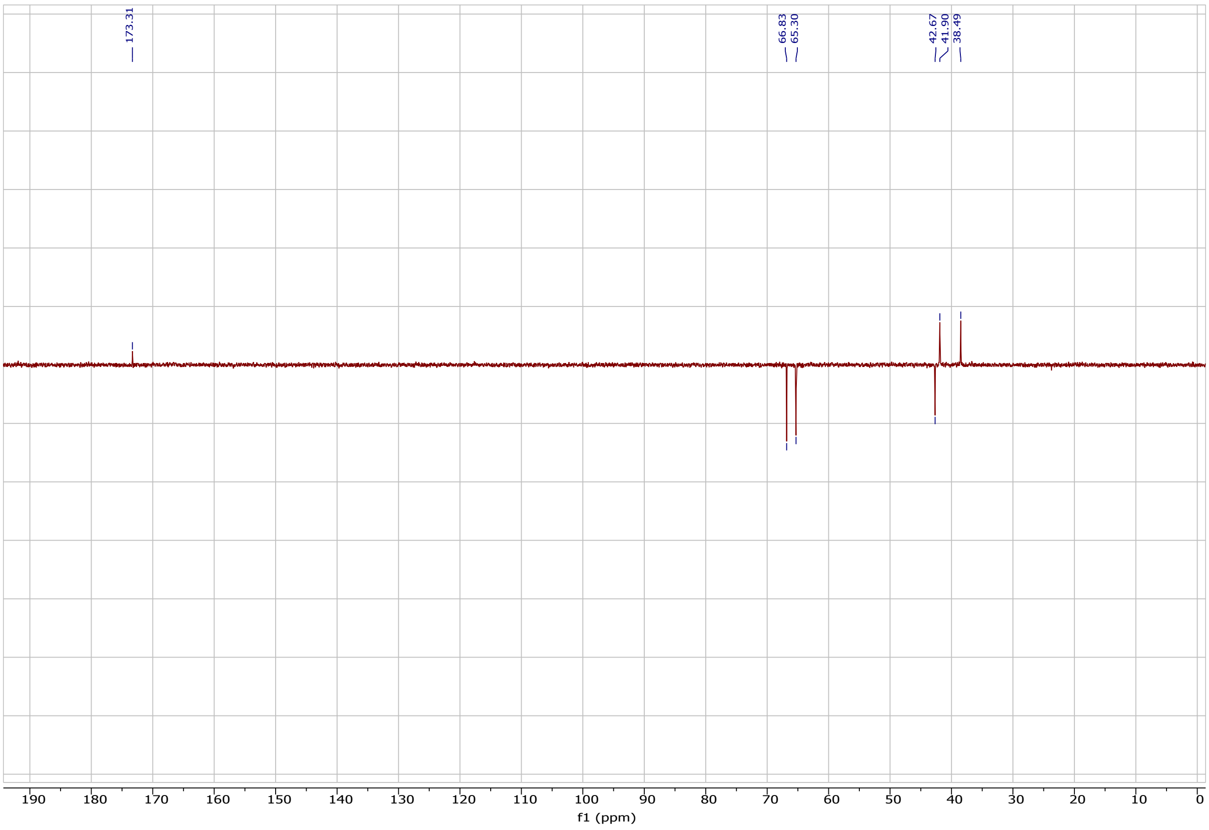

^{1}H and ^{13}C of **14** in $CDCl_3$ at 333 K

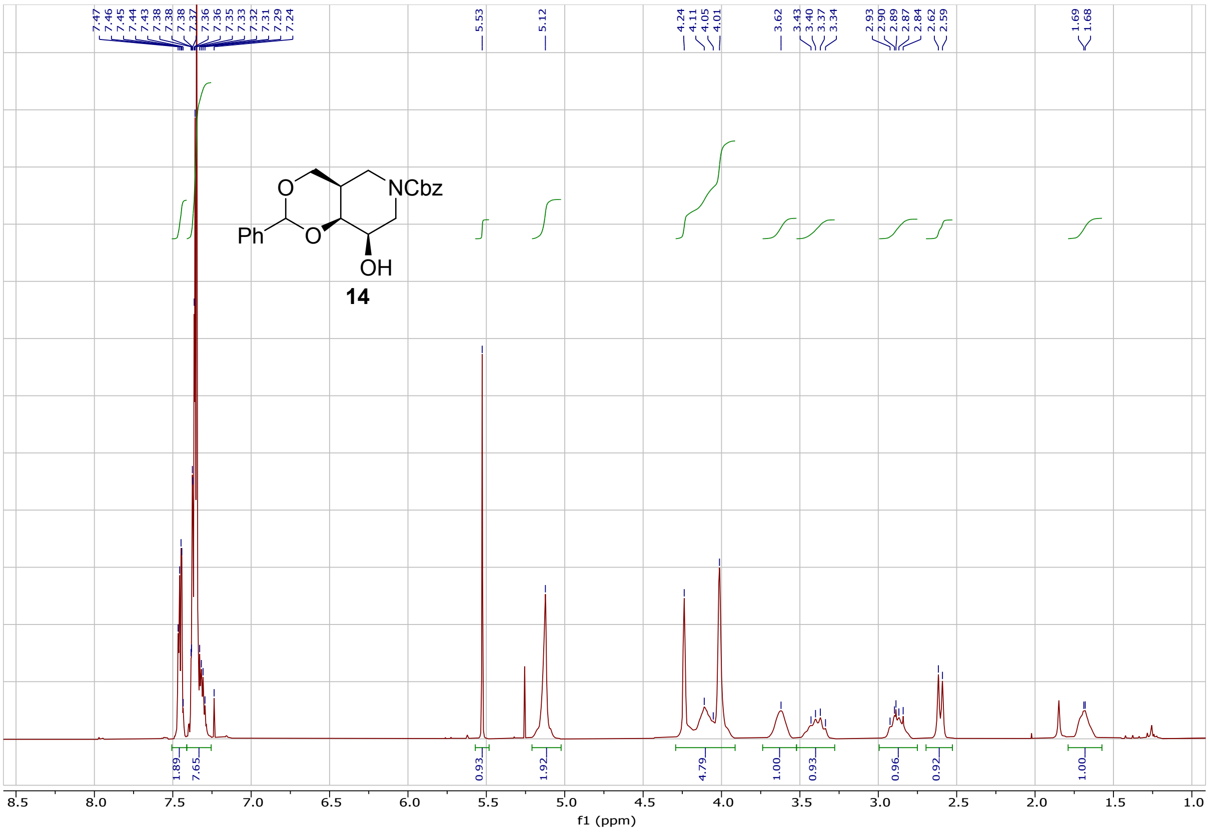

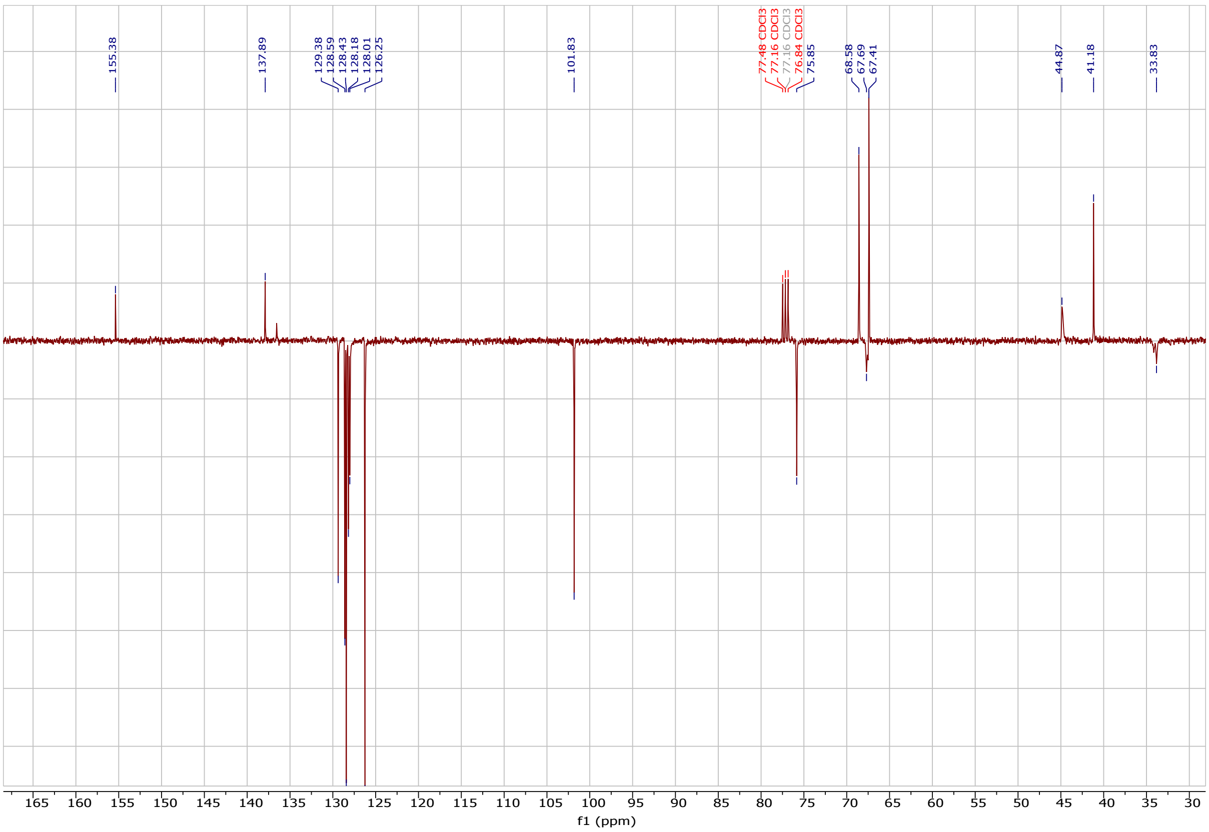

^{1}H and ^{13}C of **15** in $CDCl_3$ at 333 K

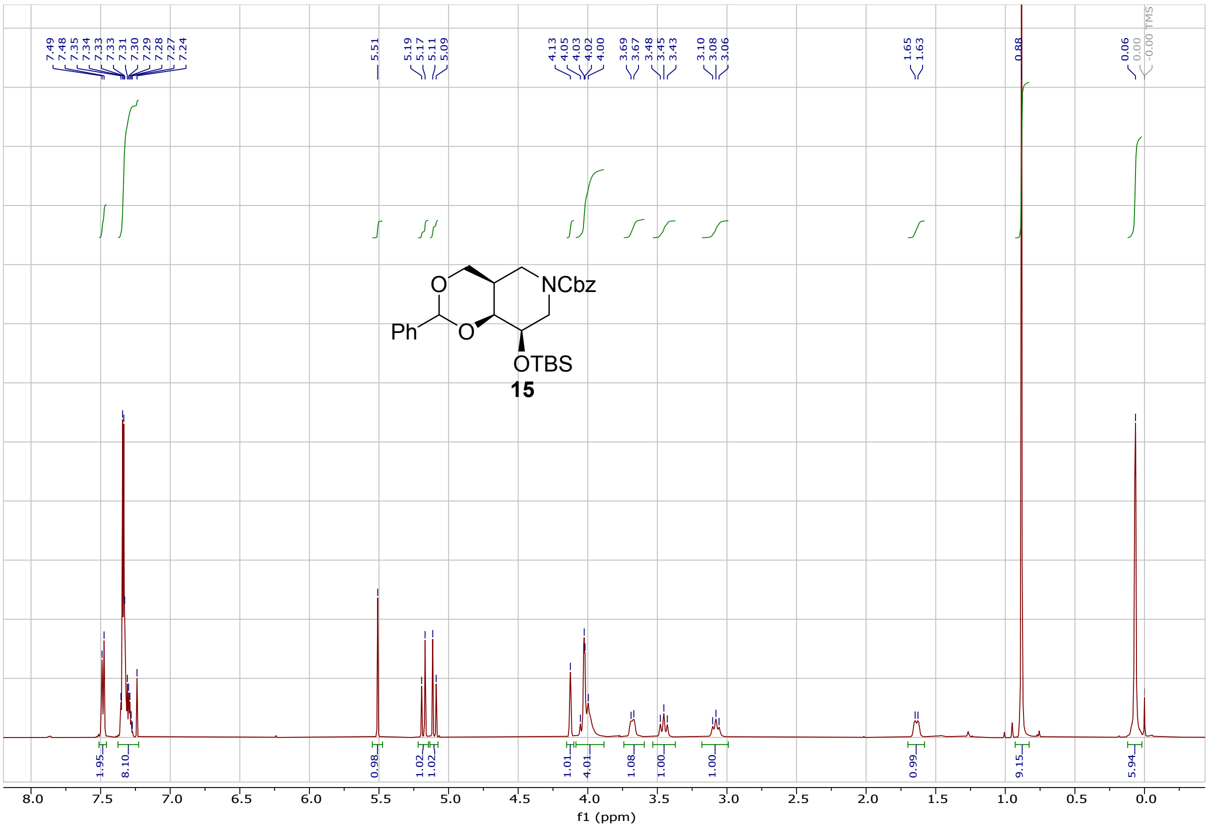

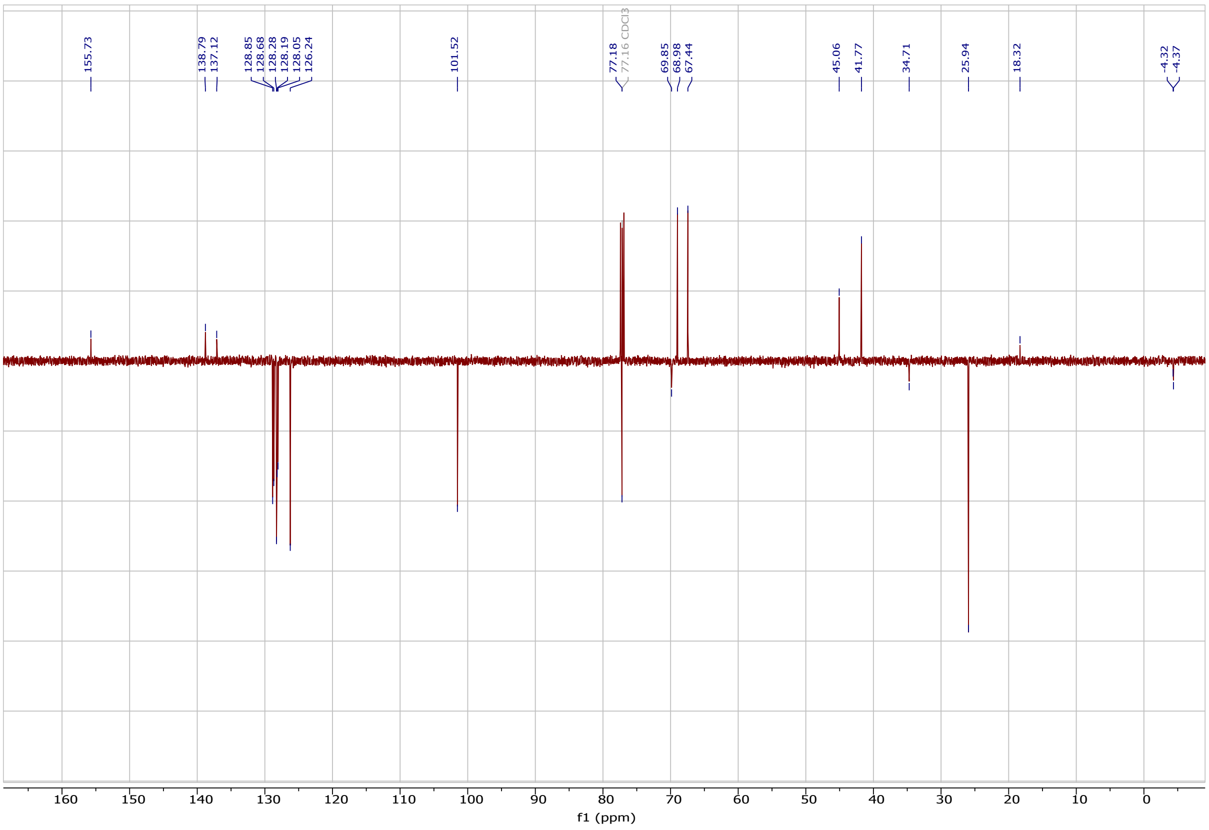

^{1}H and ^{13}C of **16** in $CDCl_3$ at 333 K

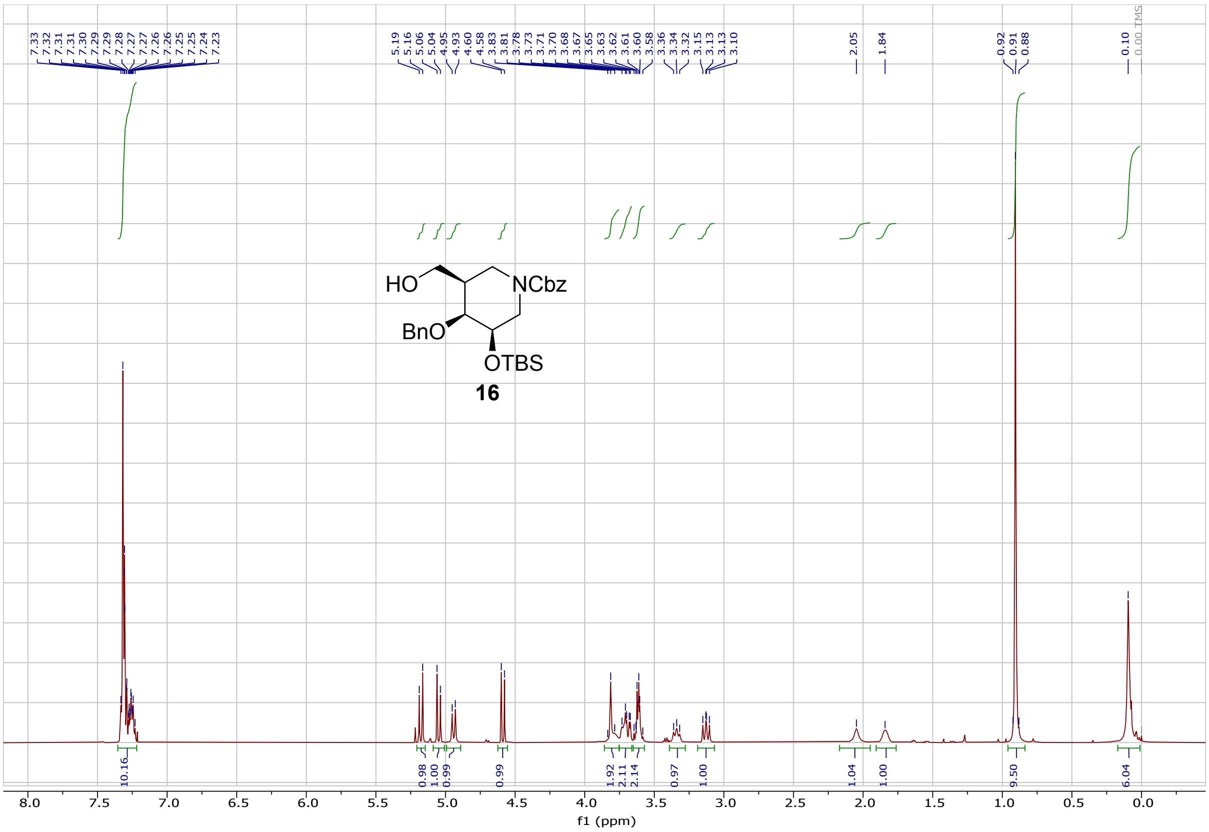

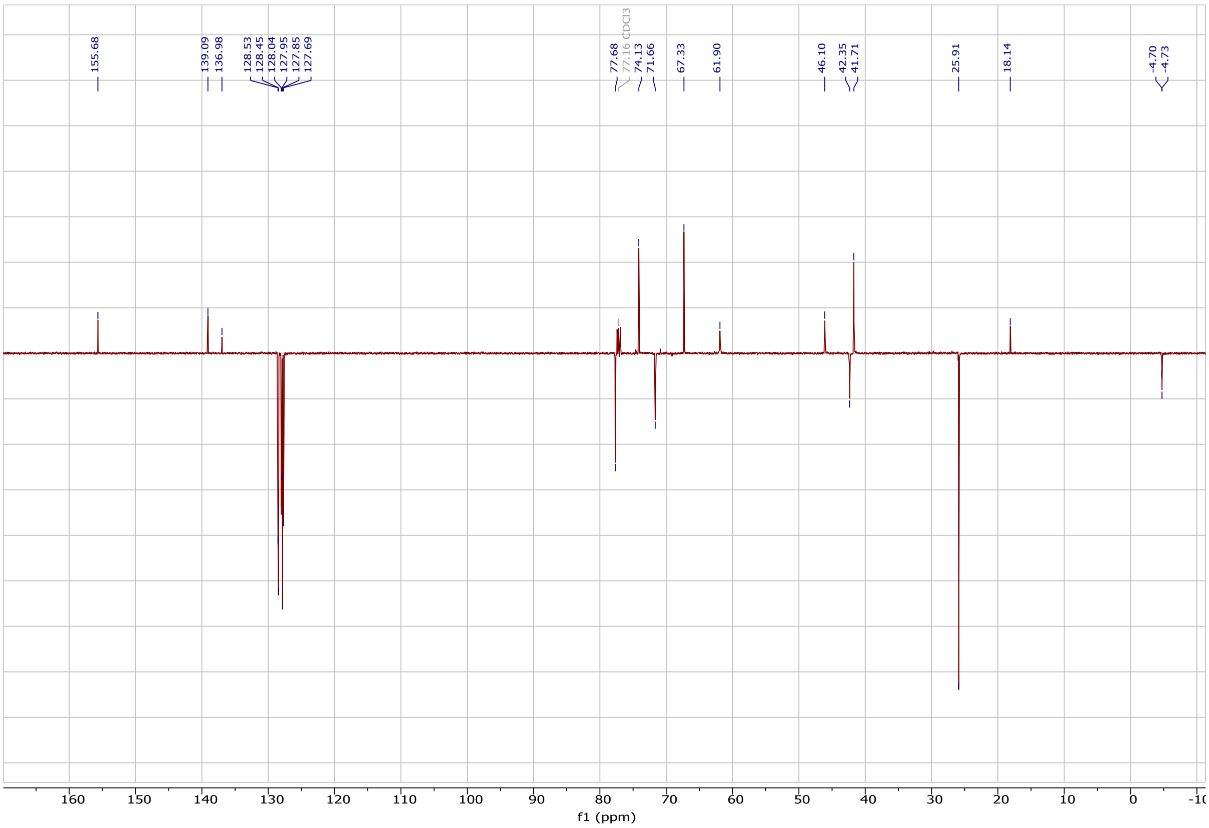

^{1}H and ^{13}C of **17** in $CDCl_3$ at 333 K

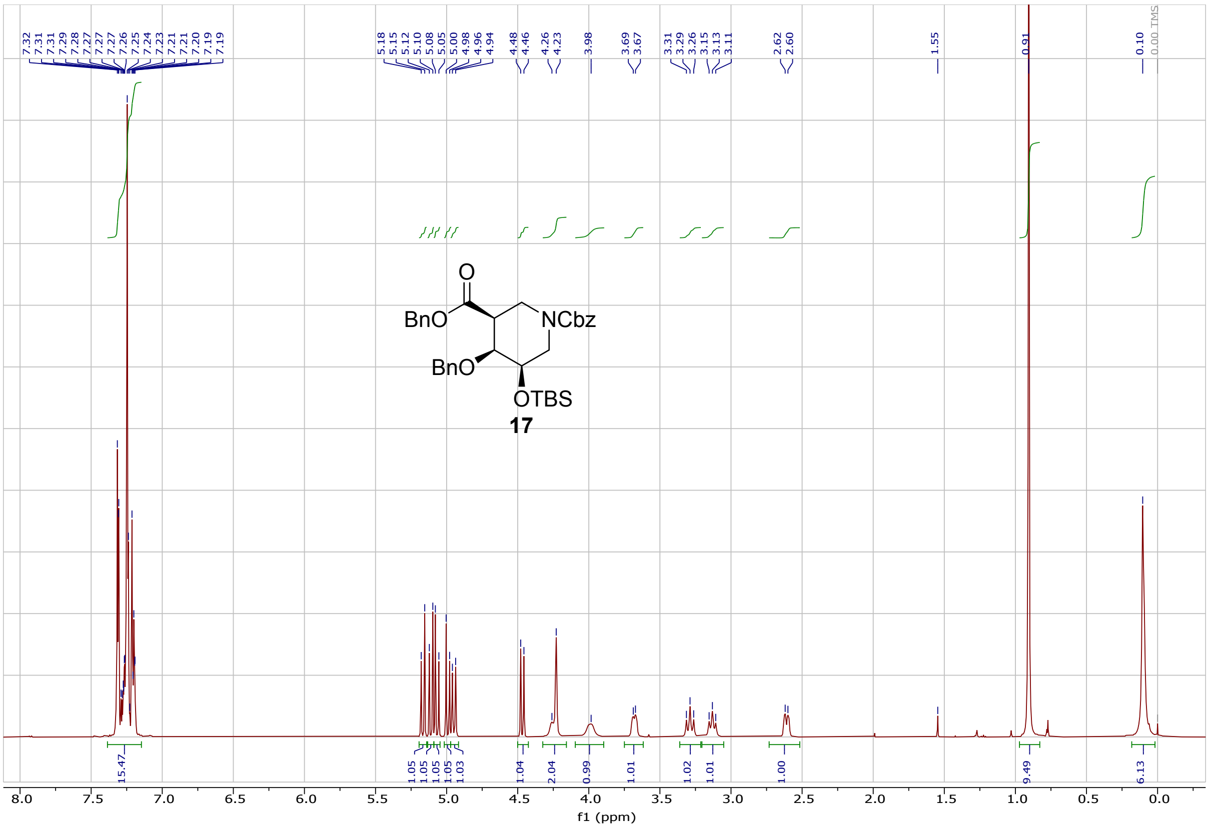

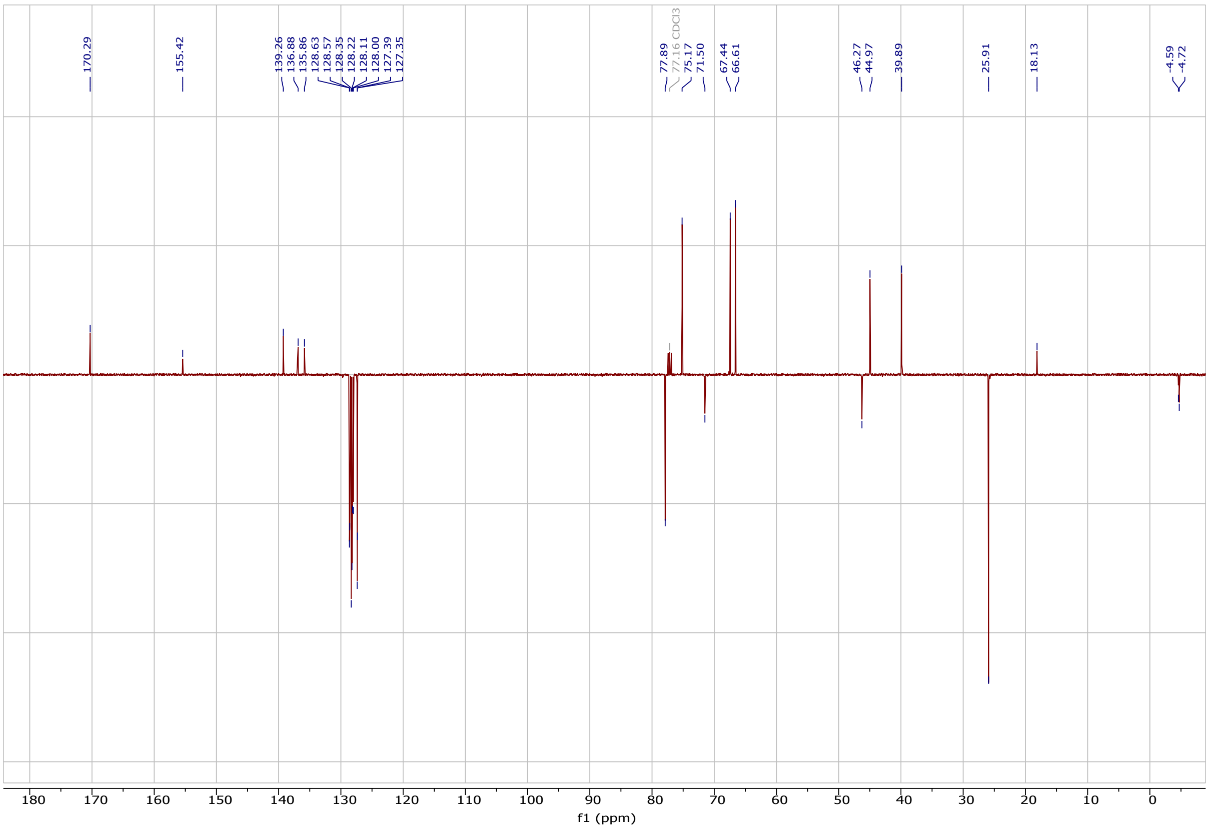

^{1}H and ^{13}C of **18** in $CDCl_3$ at 293 K

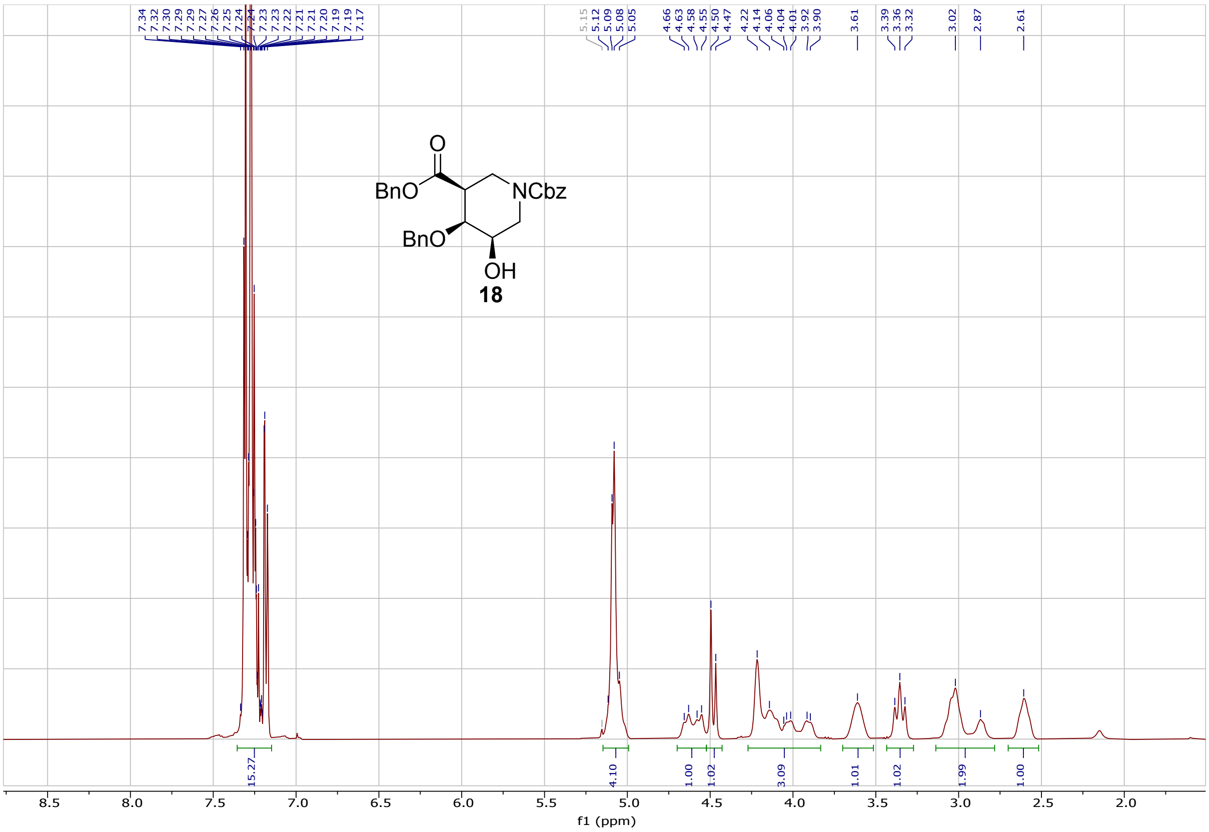

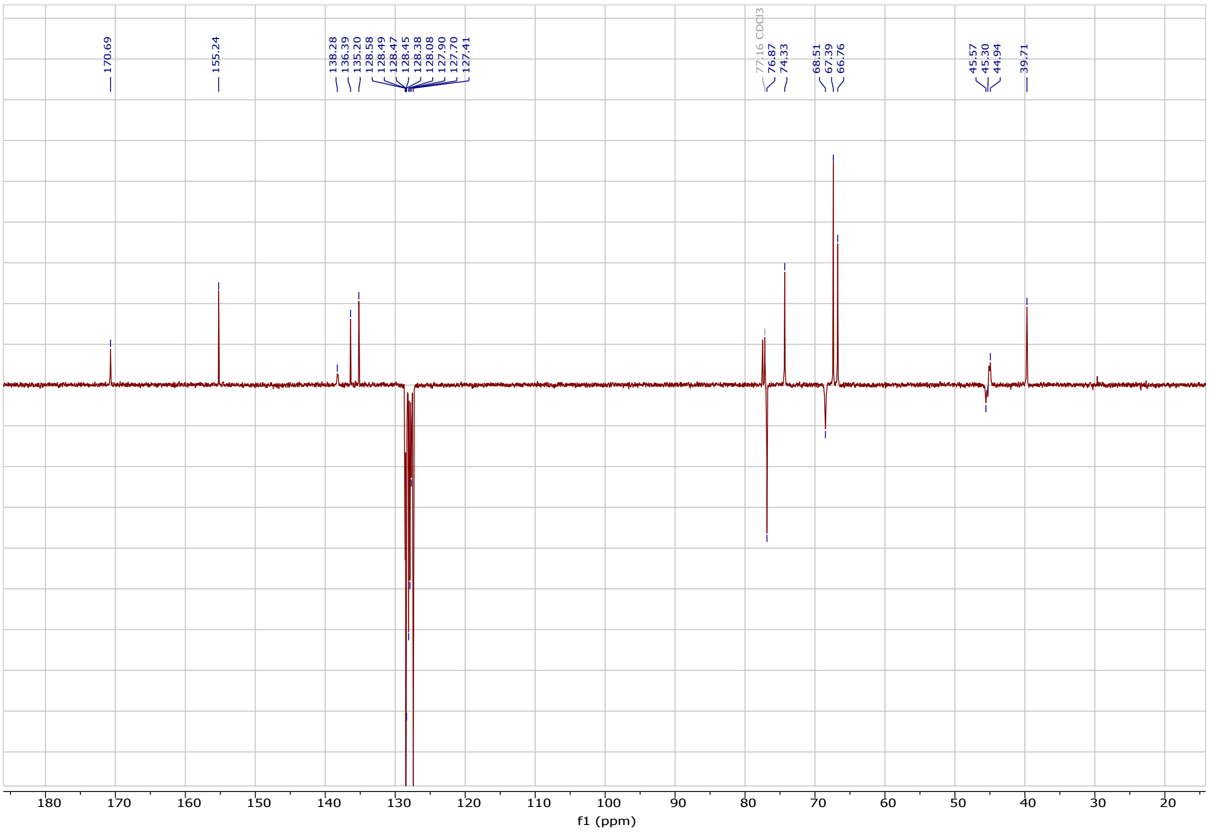

^1H and ^{13}C of **19** in $CDCl_3$ at 293 K

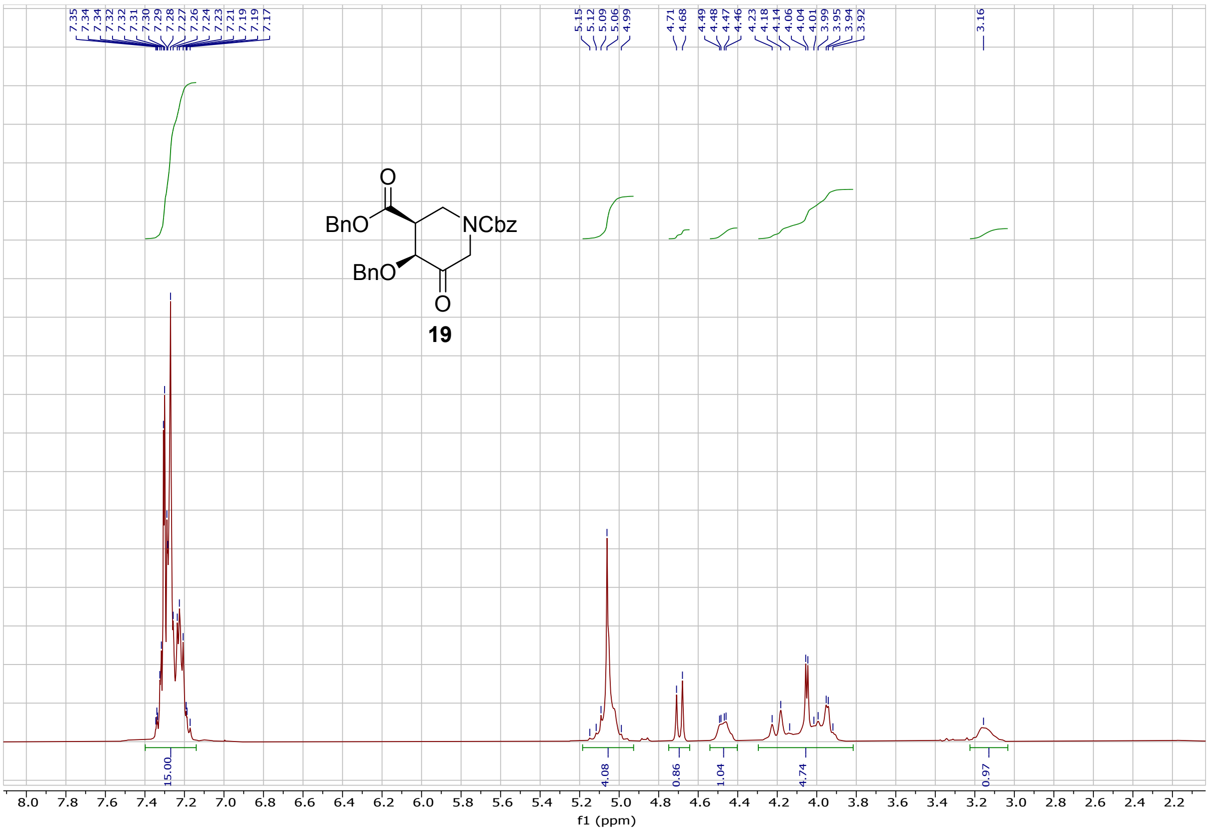

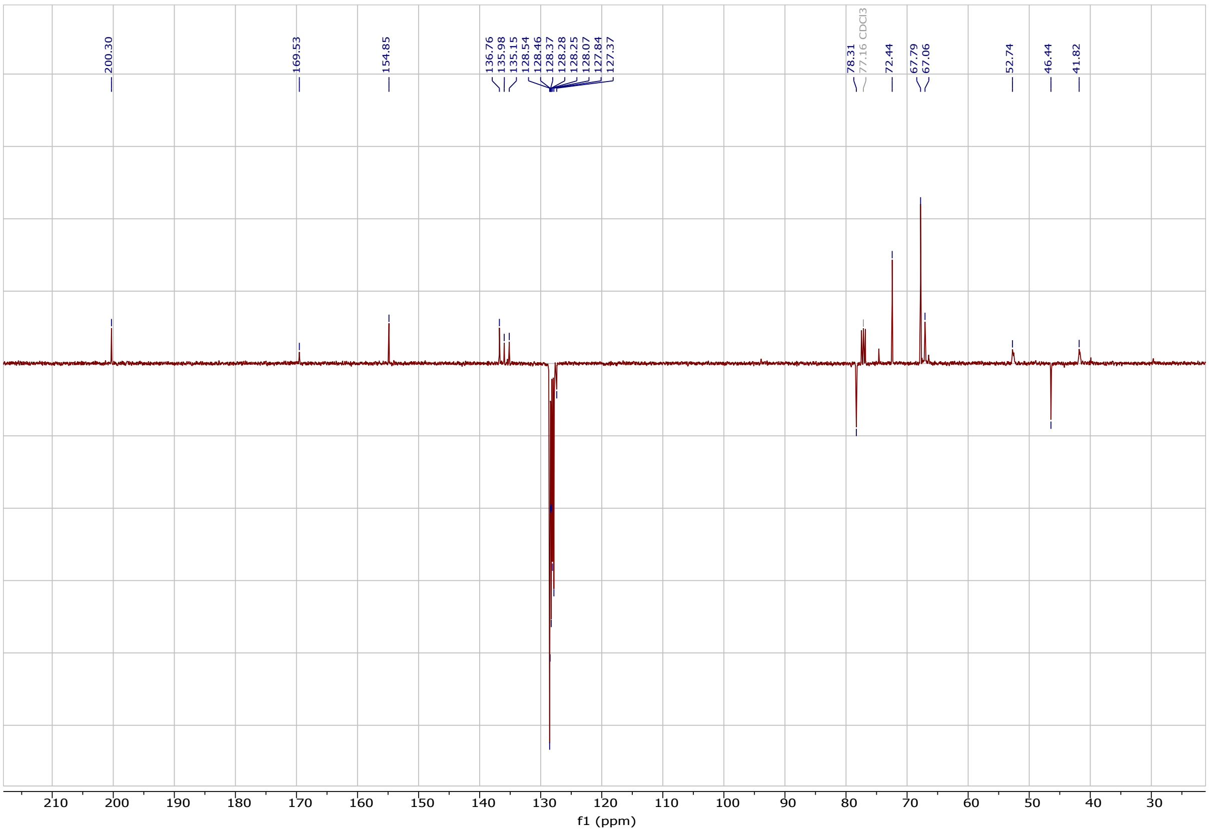

^1H and ^{13}C of **S4** in $CDCl_3$ at 333 K

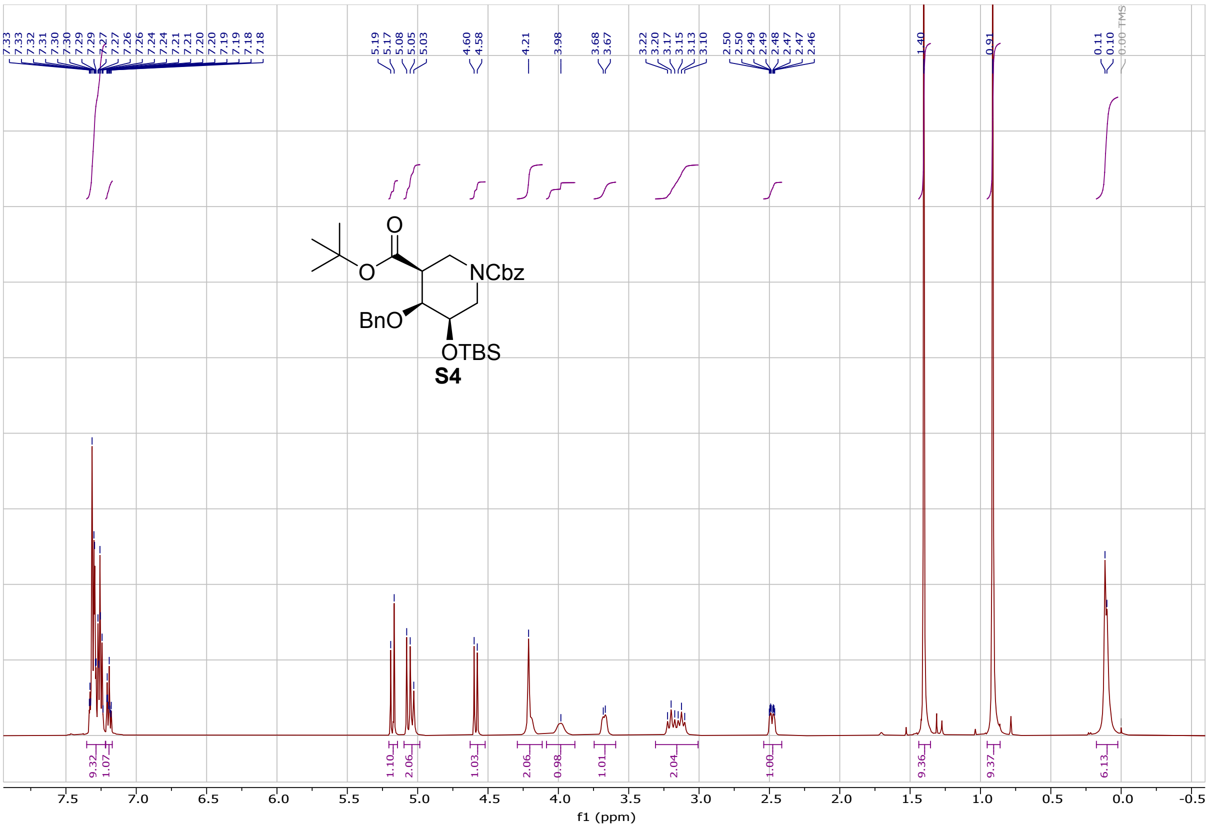

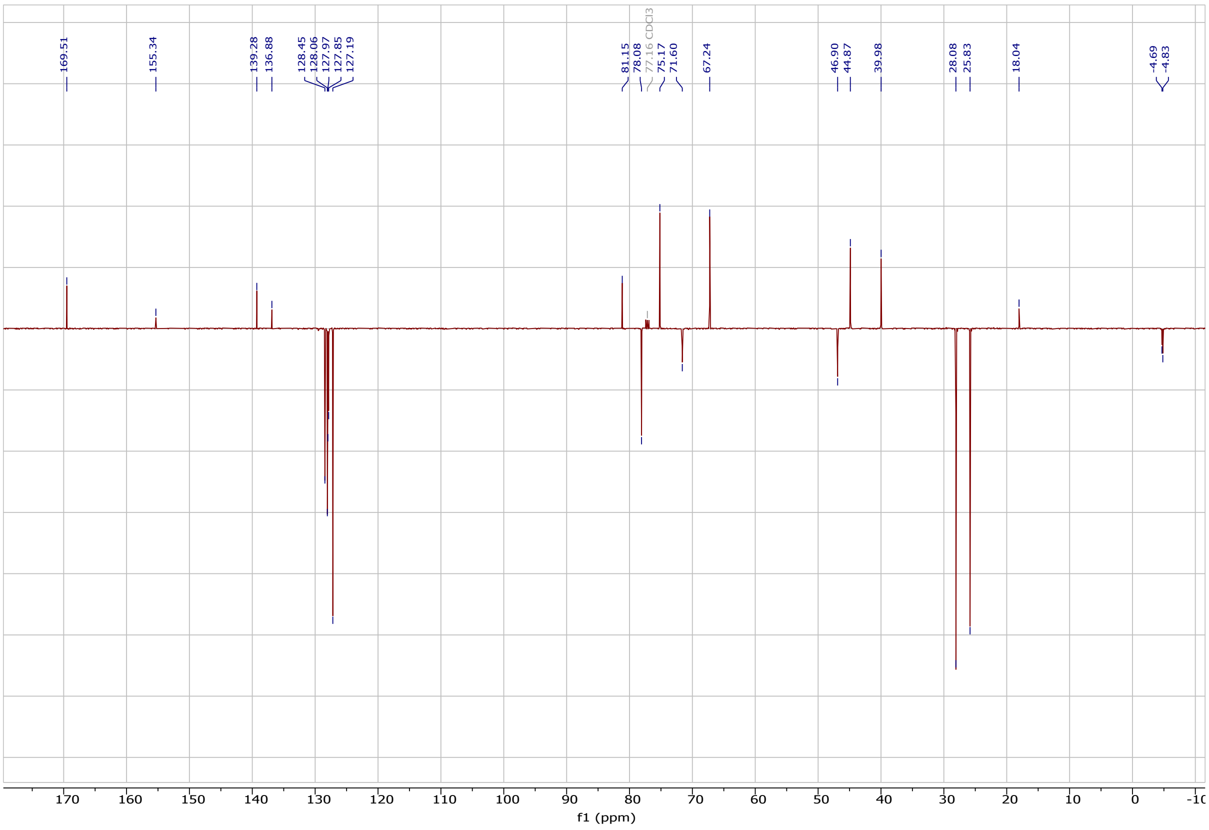

^{1}H and ^{13}C of **S5** in $CDCl_3$ at 333 K

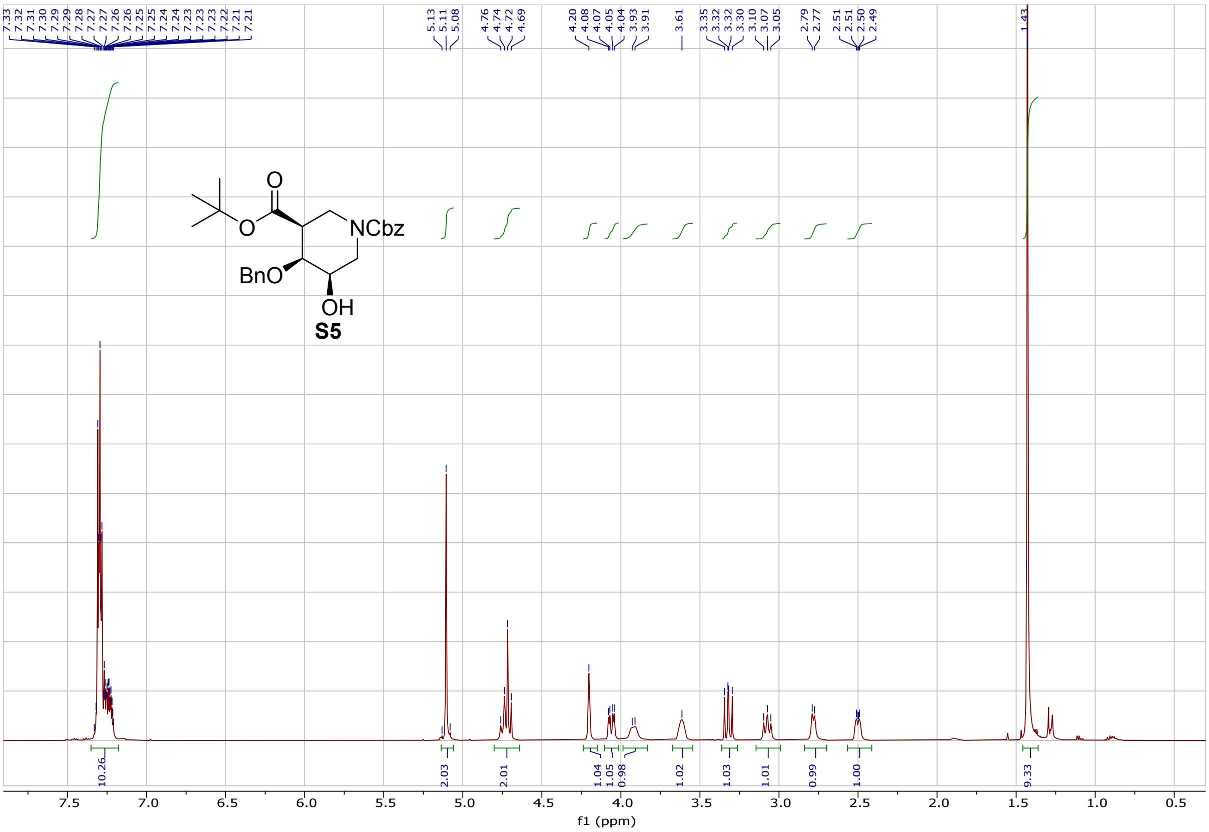

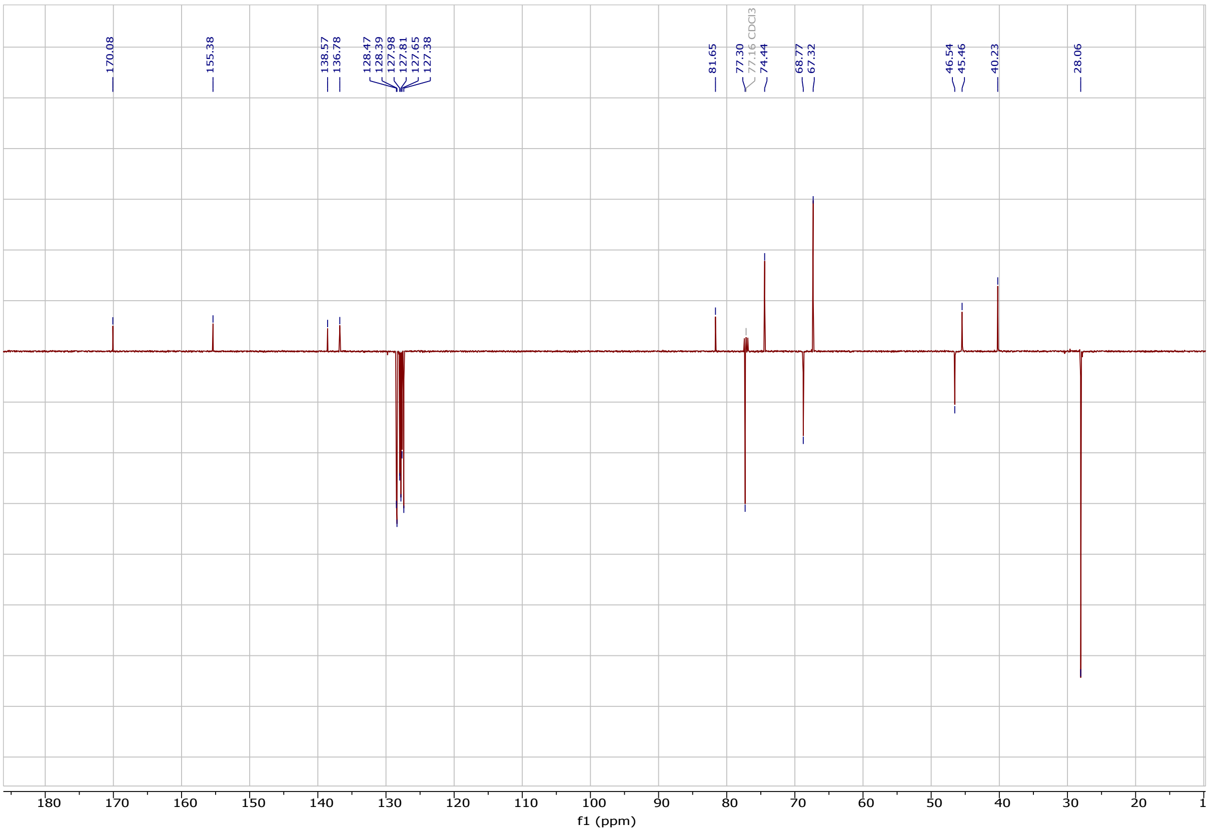

[1]H and ^{13}C of **S6** in $CDCl_3$ at 293 K

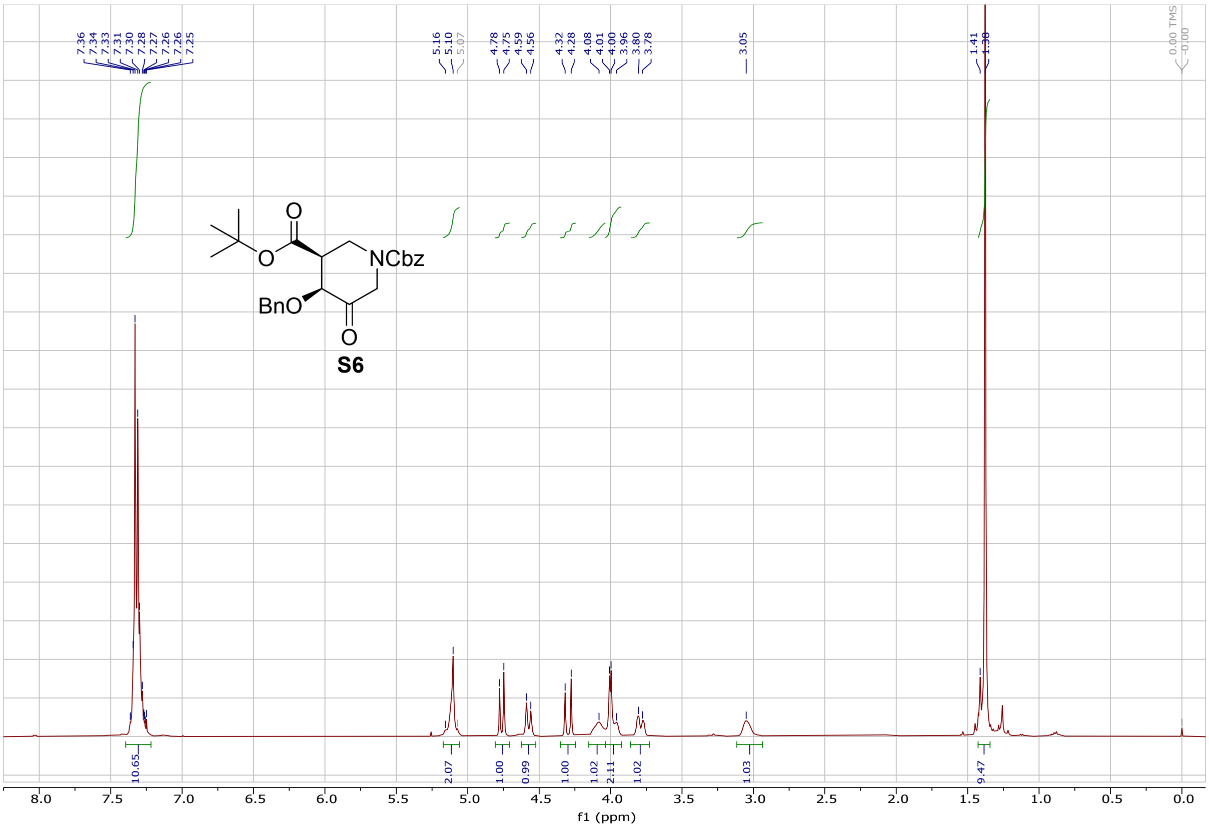

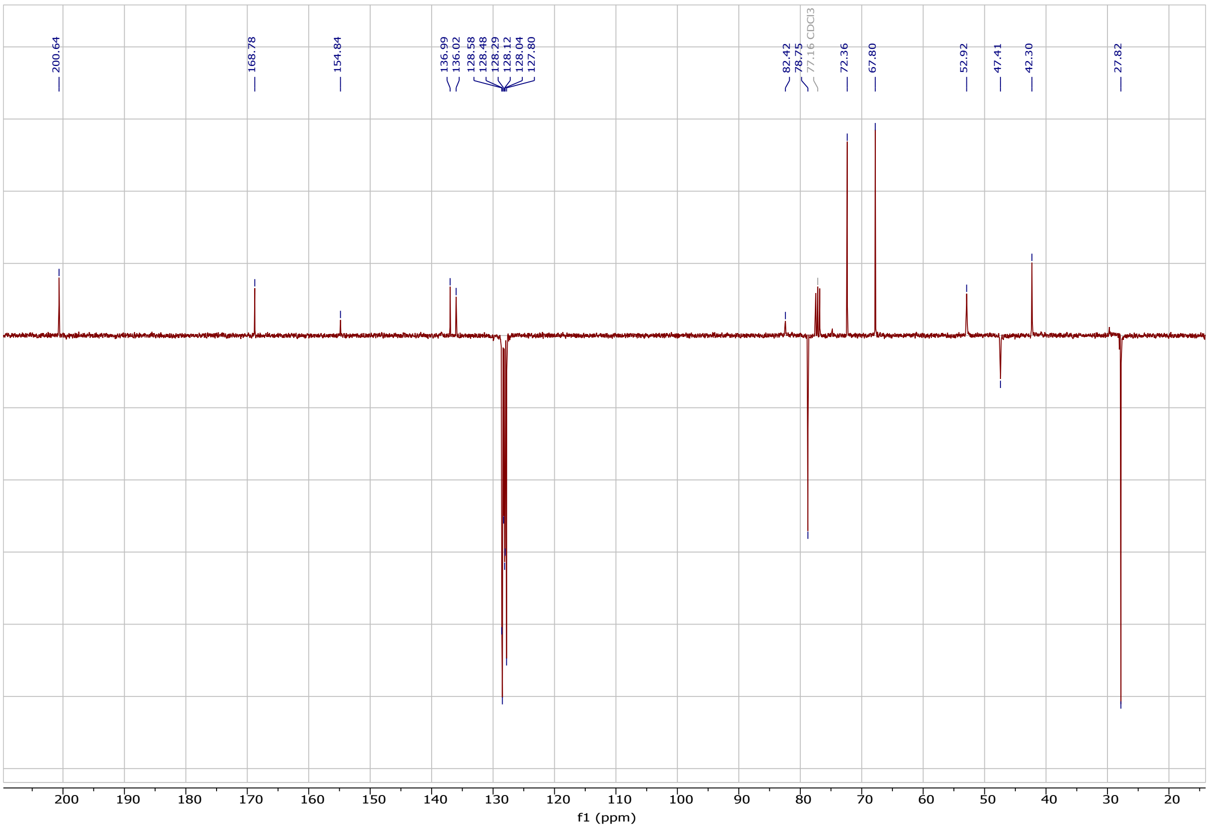

^{1}H and ^{13}C of **S7** in $CDCl_3$ at 333 K

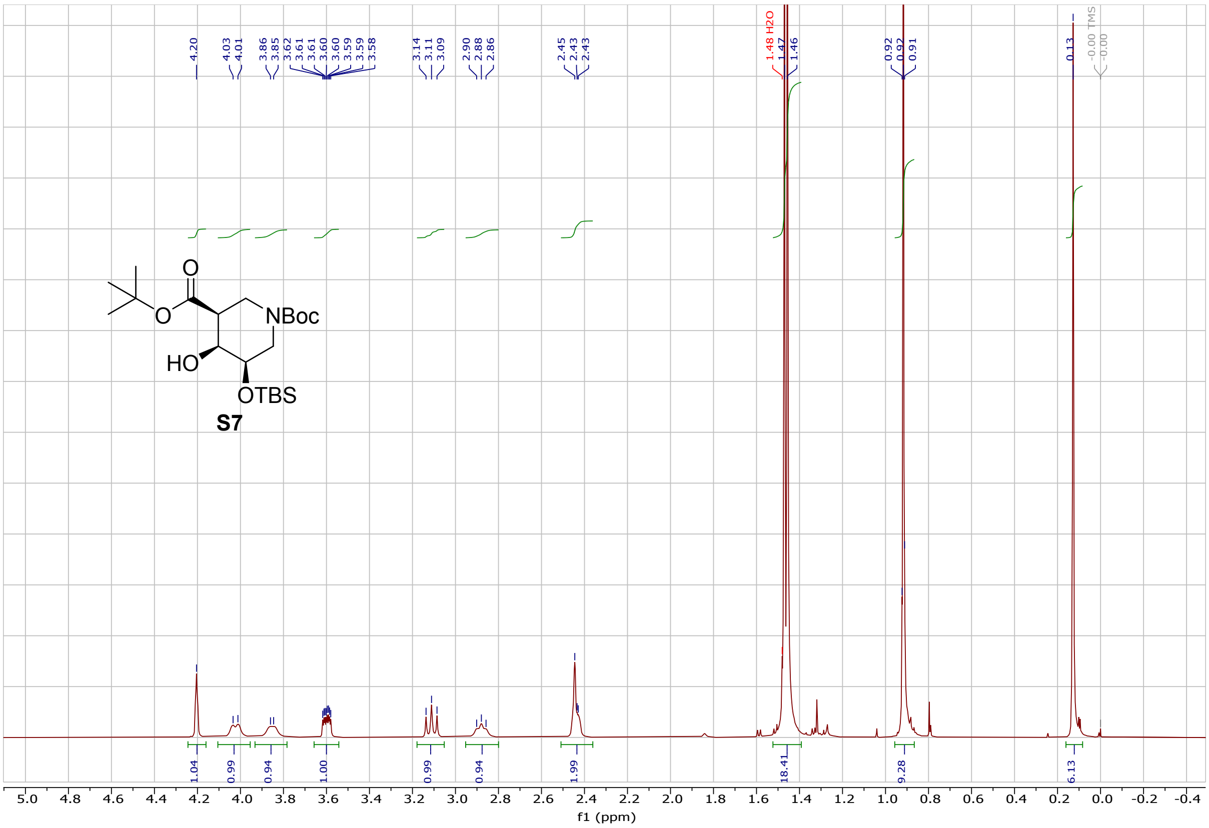

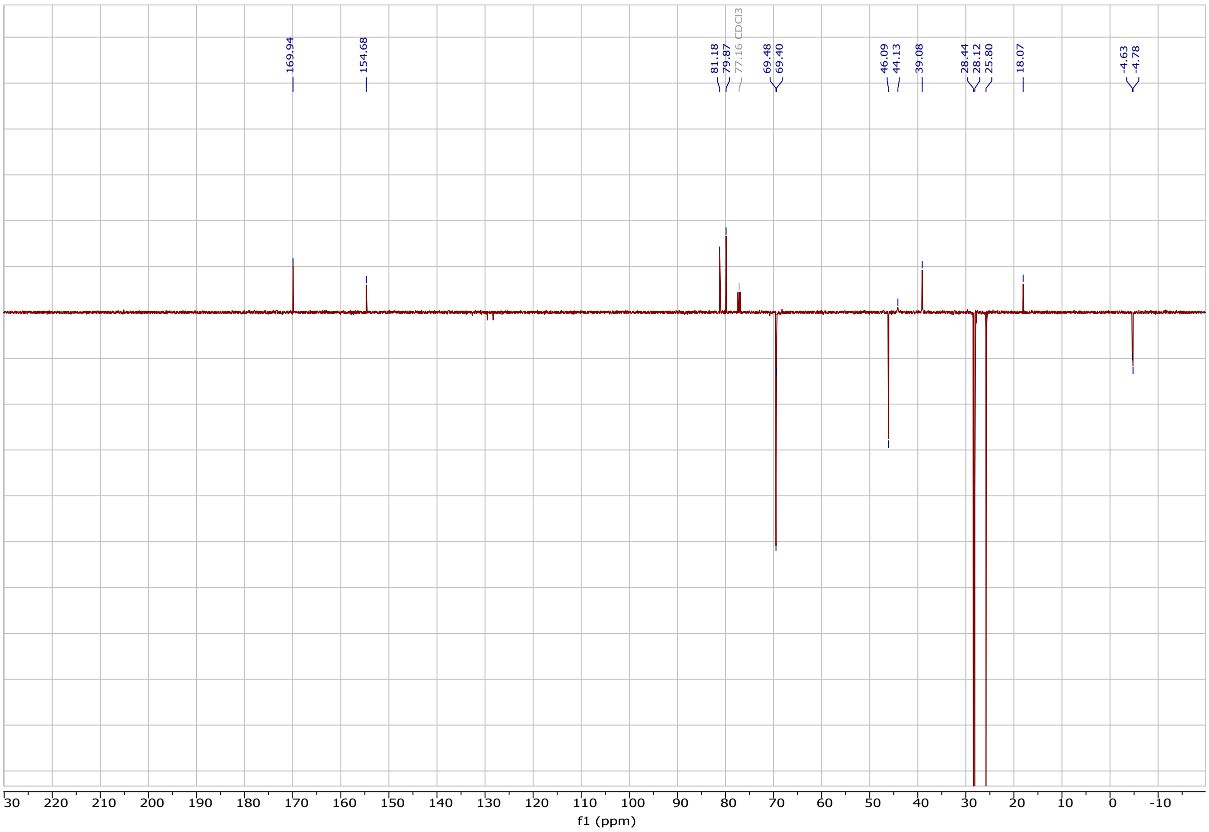

^{1}H and ^{13}C of **S8** in $CDCl_3$ at 333 K

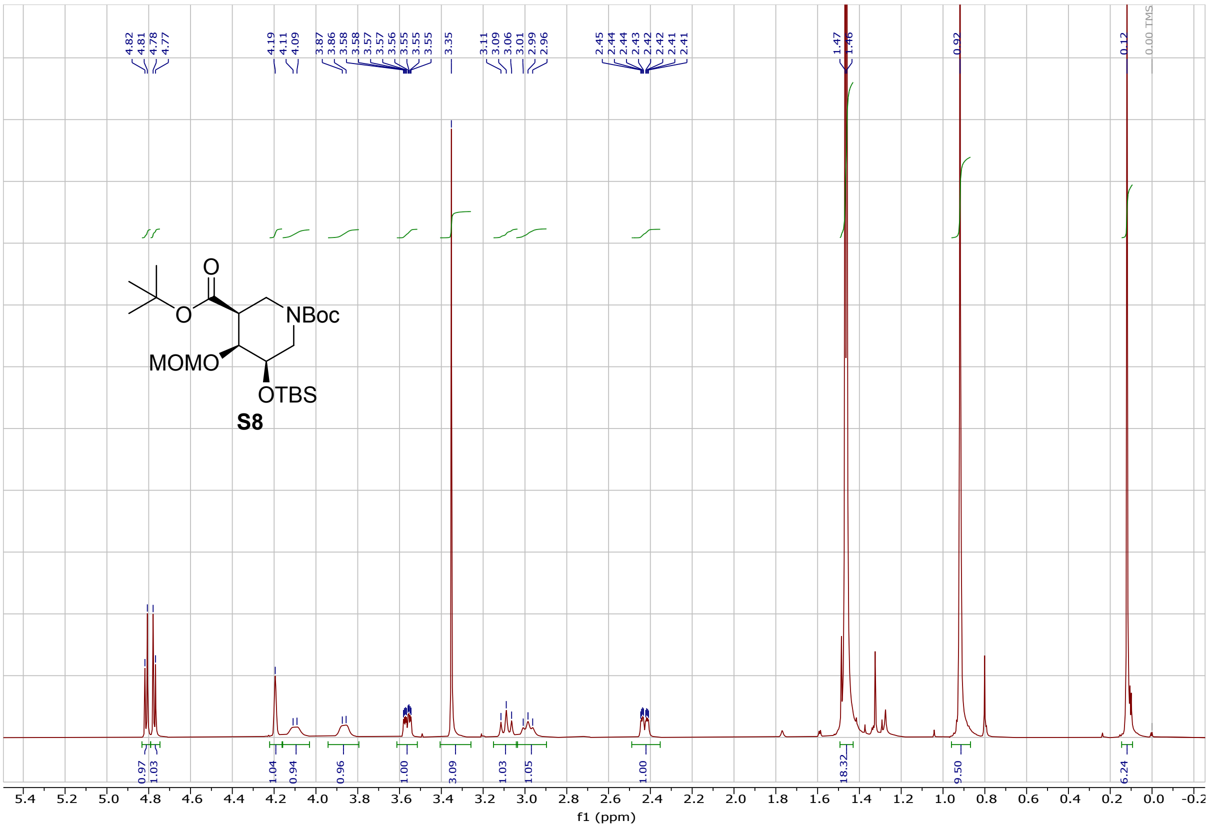

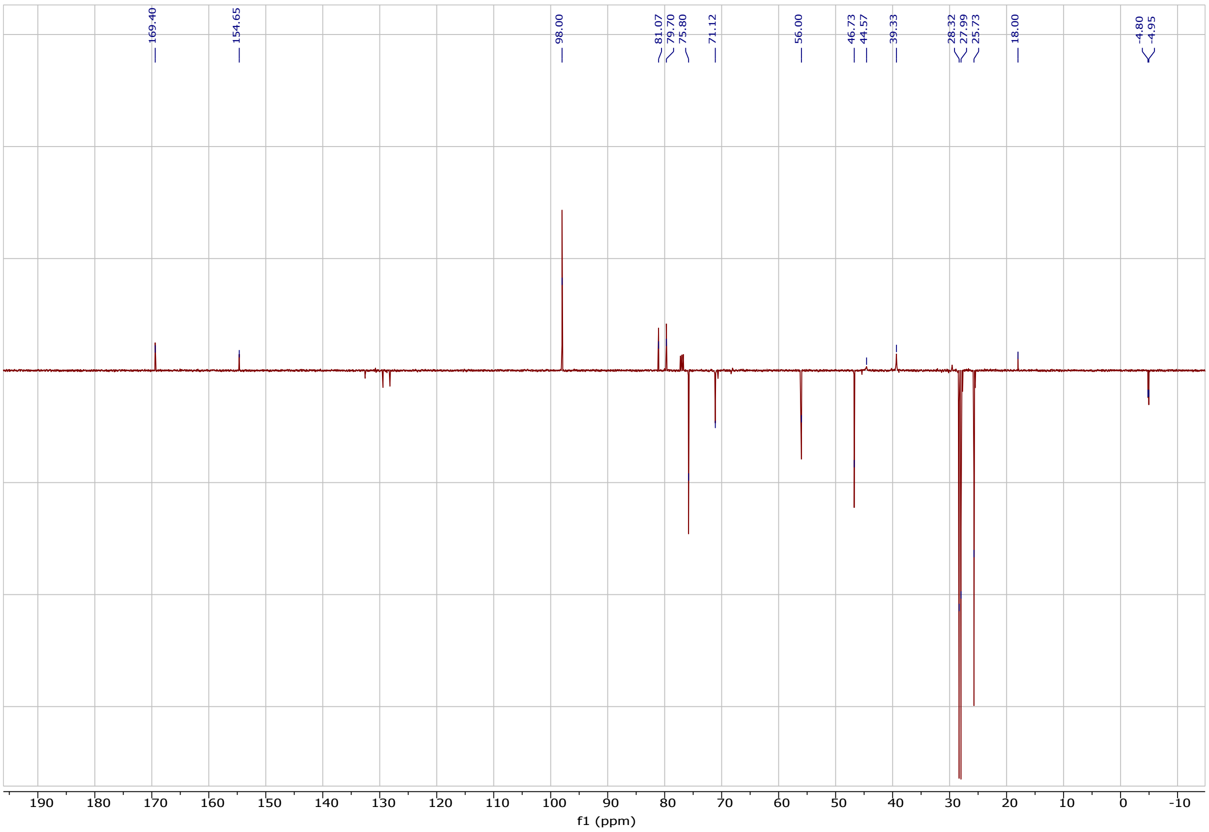

^{1}H and ^{13}C of **S9** in $CDCl_3$ at 333 K

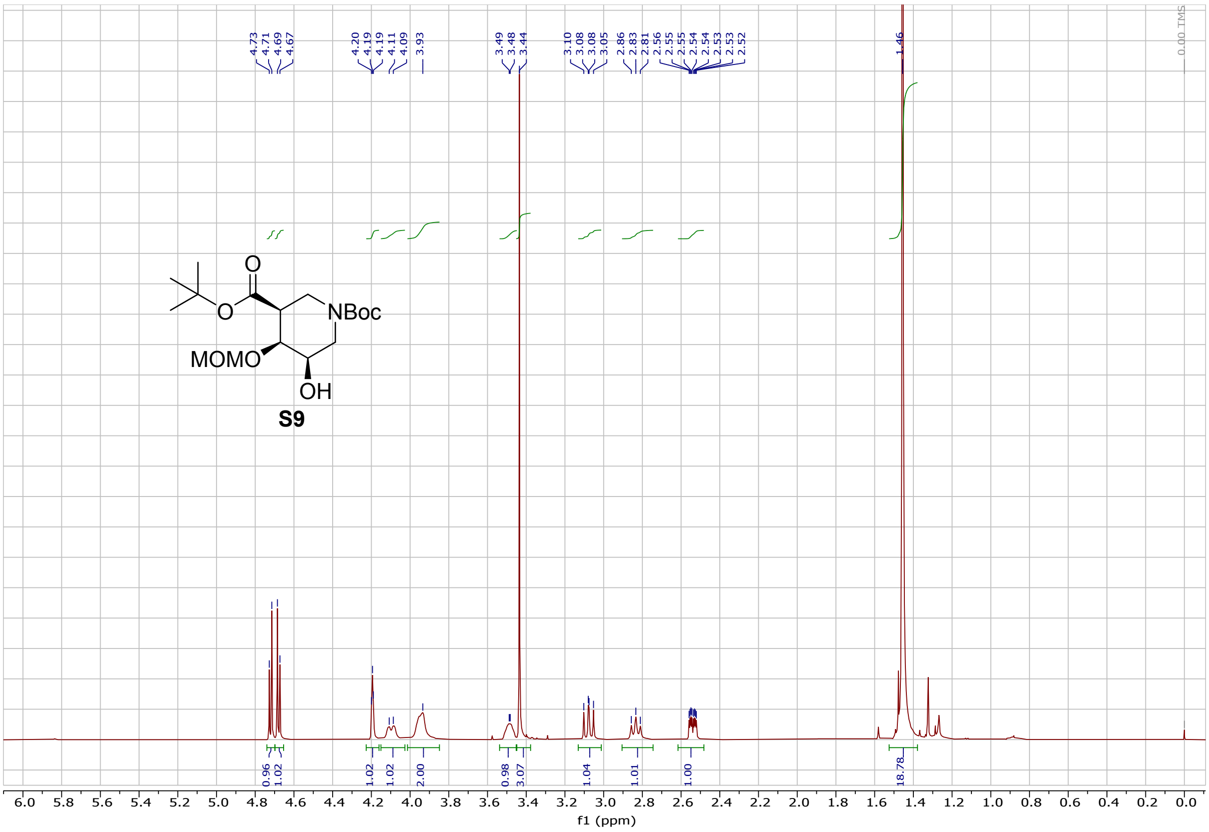

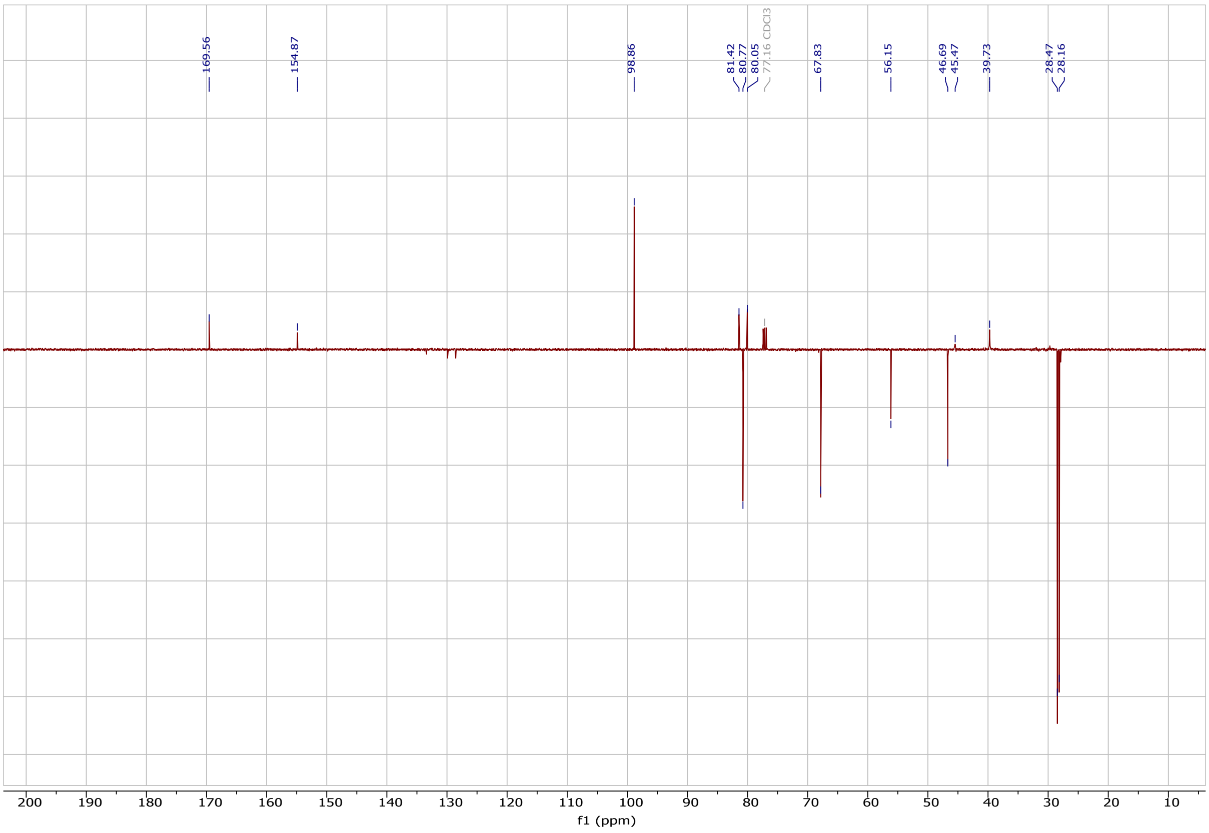

^{1}H and ^{13}C of **S10** in $CDCl_3$ at 333 K

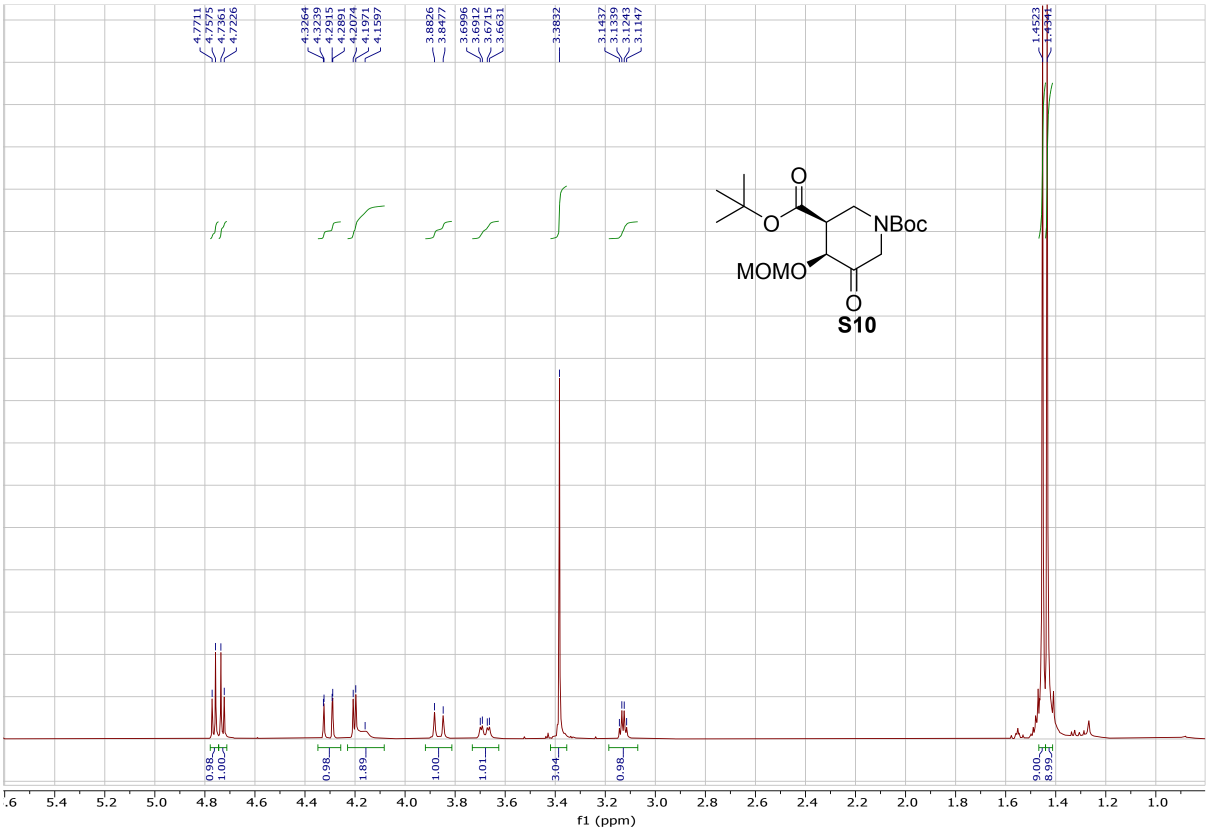

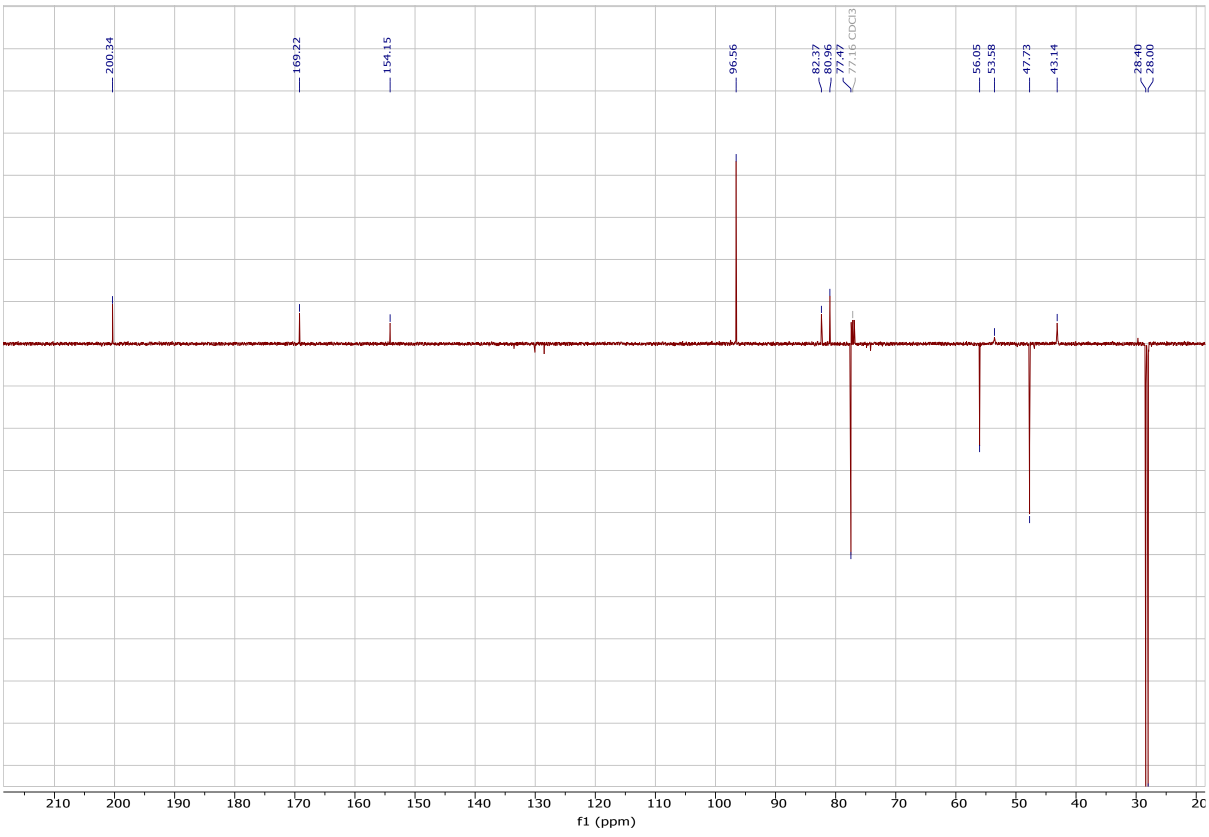

^{1}H and ^{13}C of **8** in D_2O at 293 K

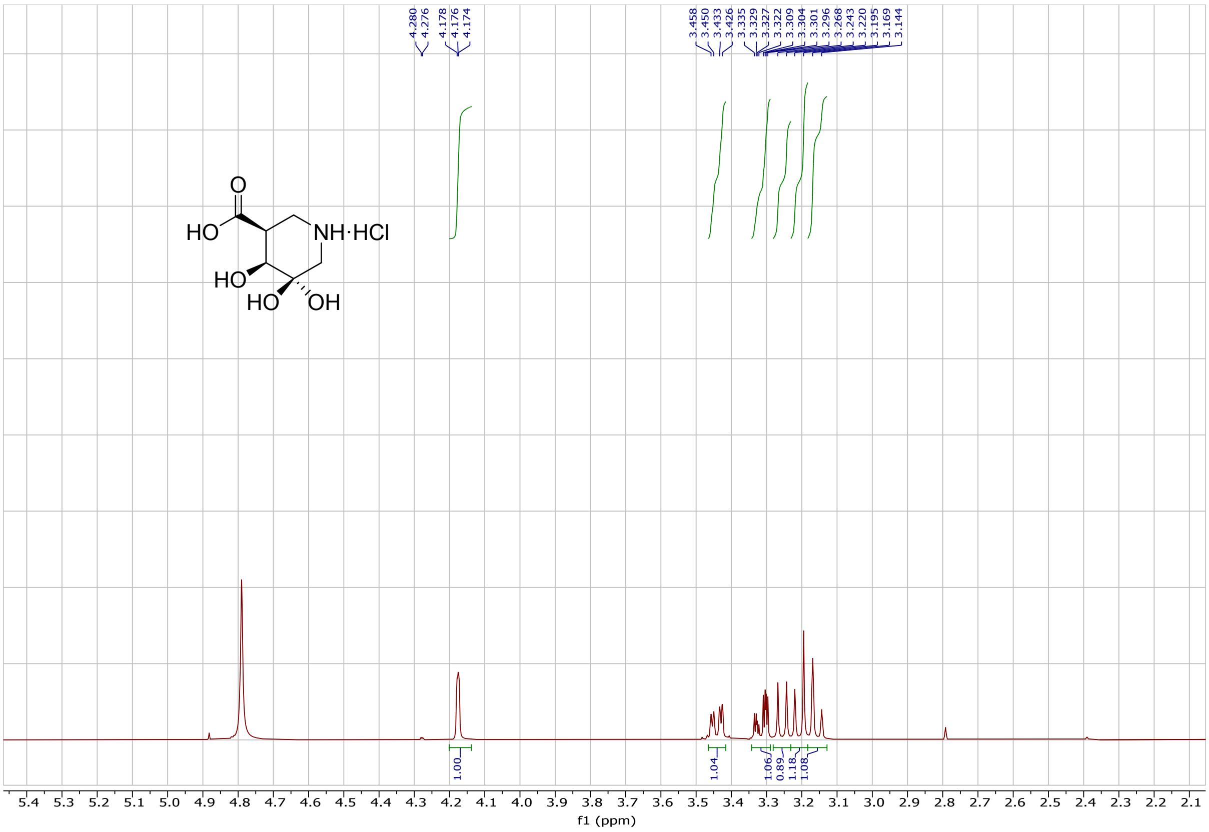

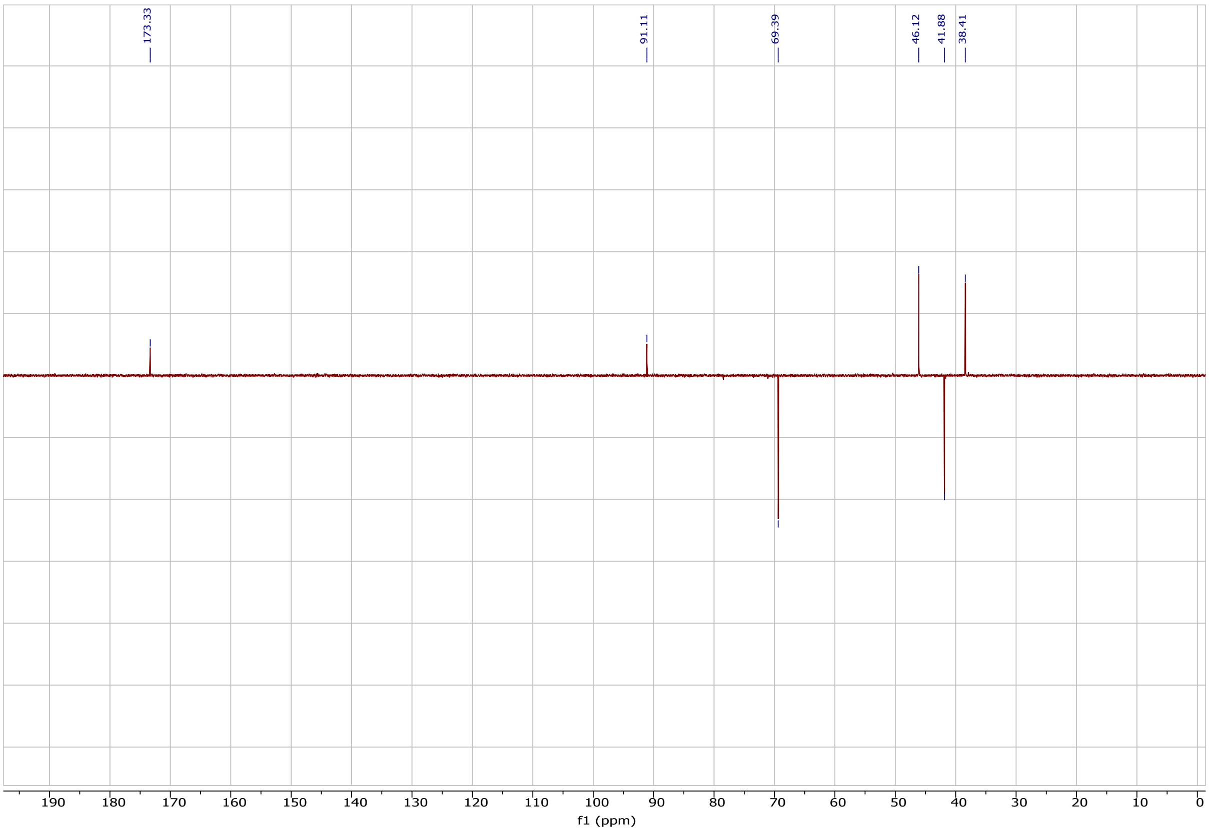

[1]H and ^{13}C of **21** in $CDCl_3$ at 293 K

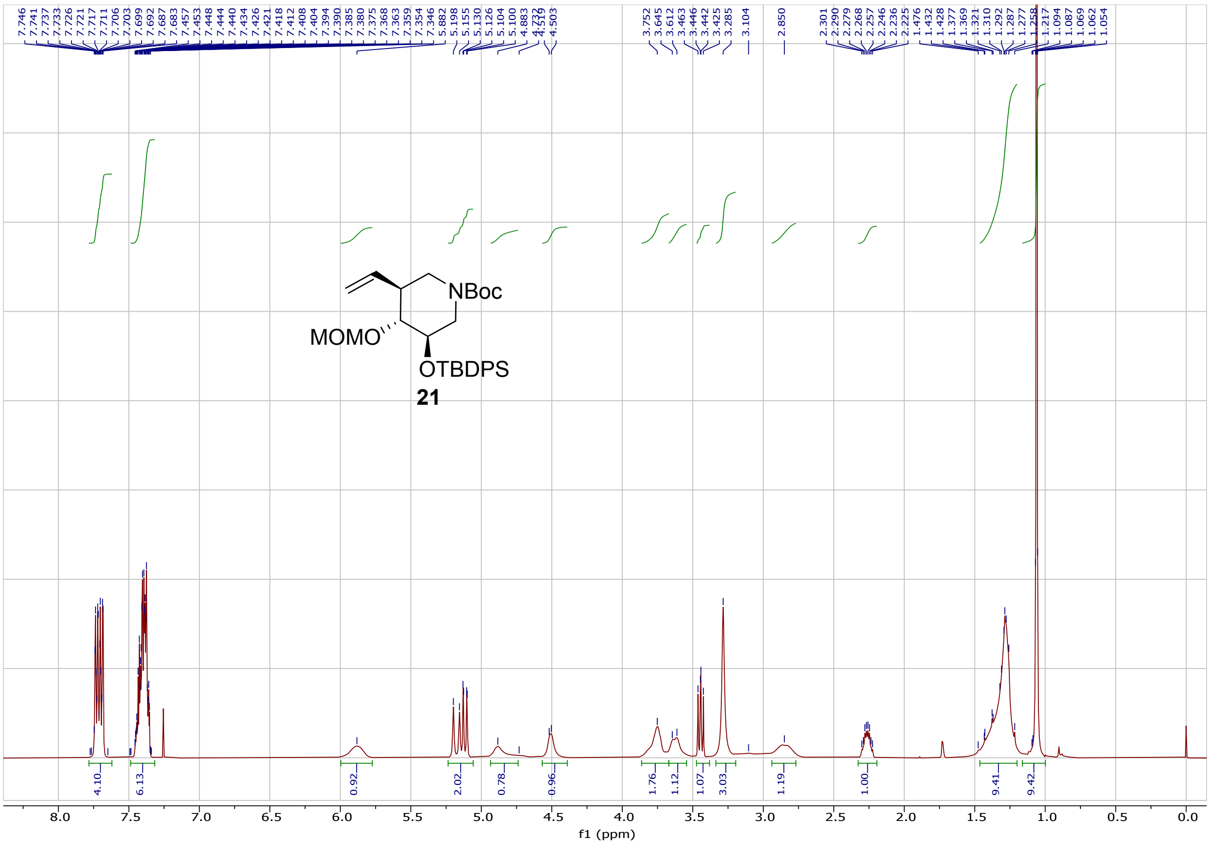

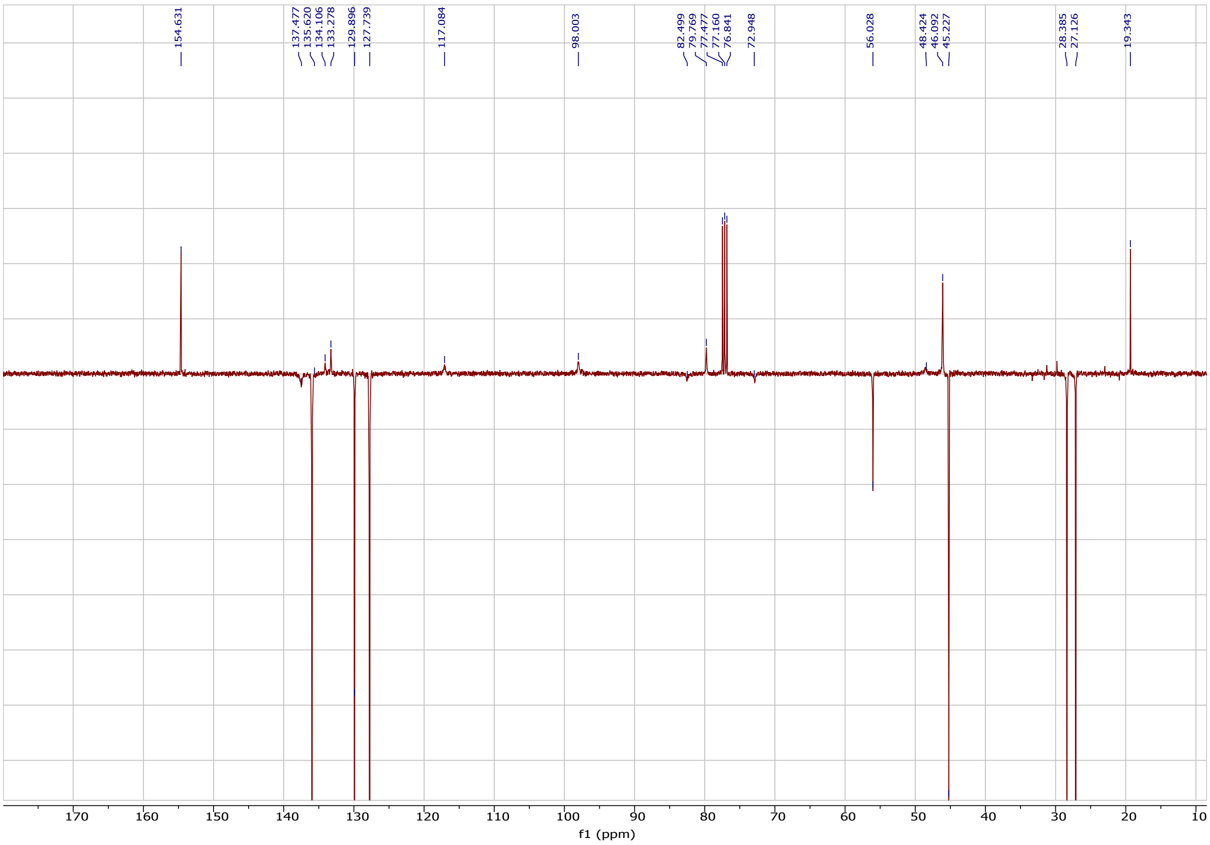

^1H and ^{13}C of **22** in $CDCl_3$ at 293 K

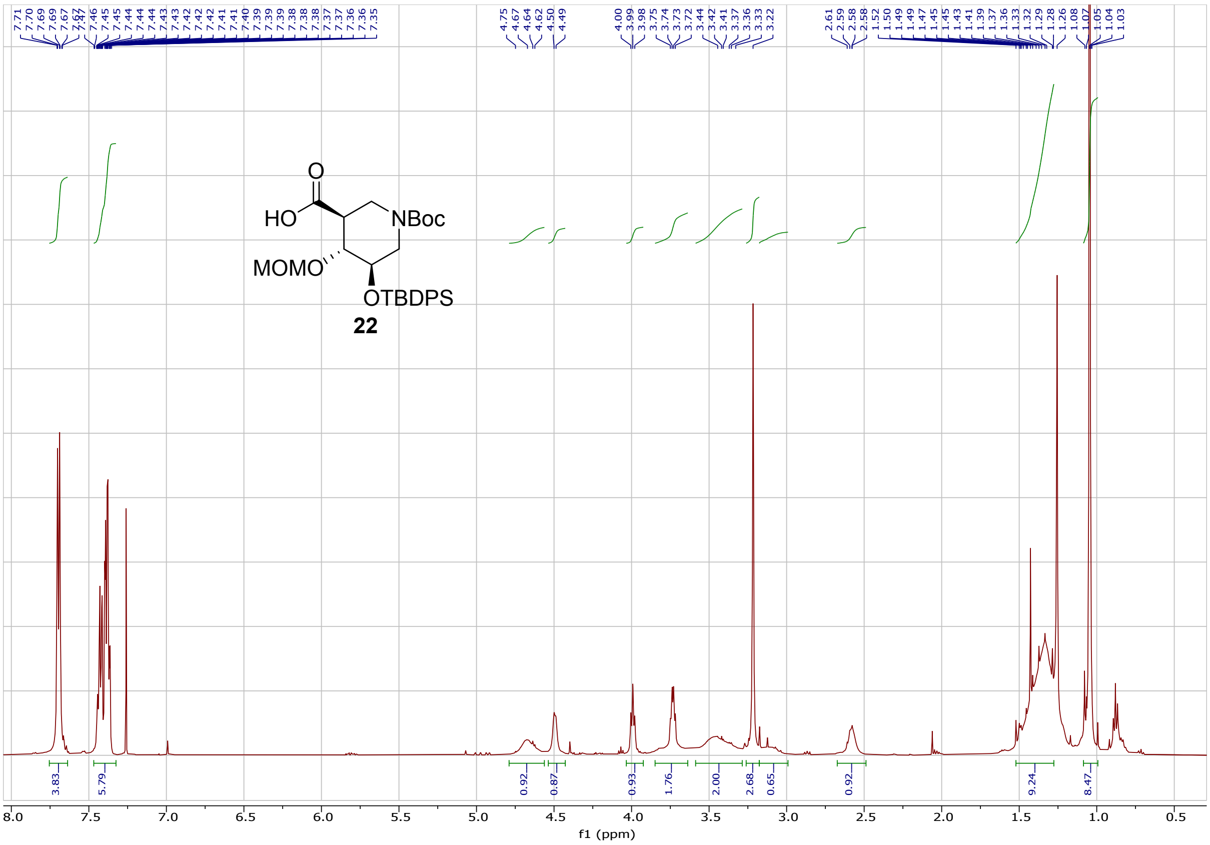

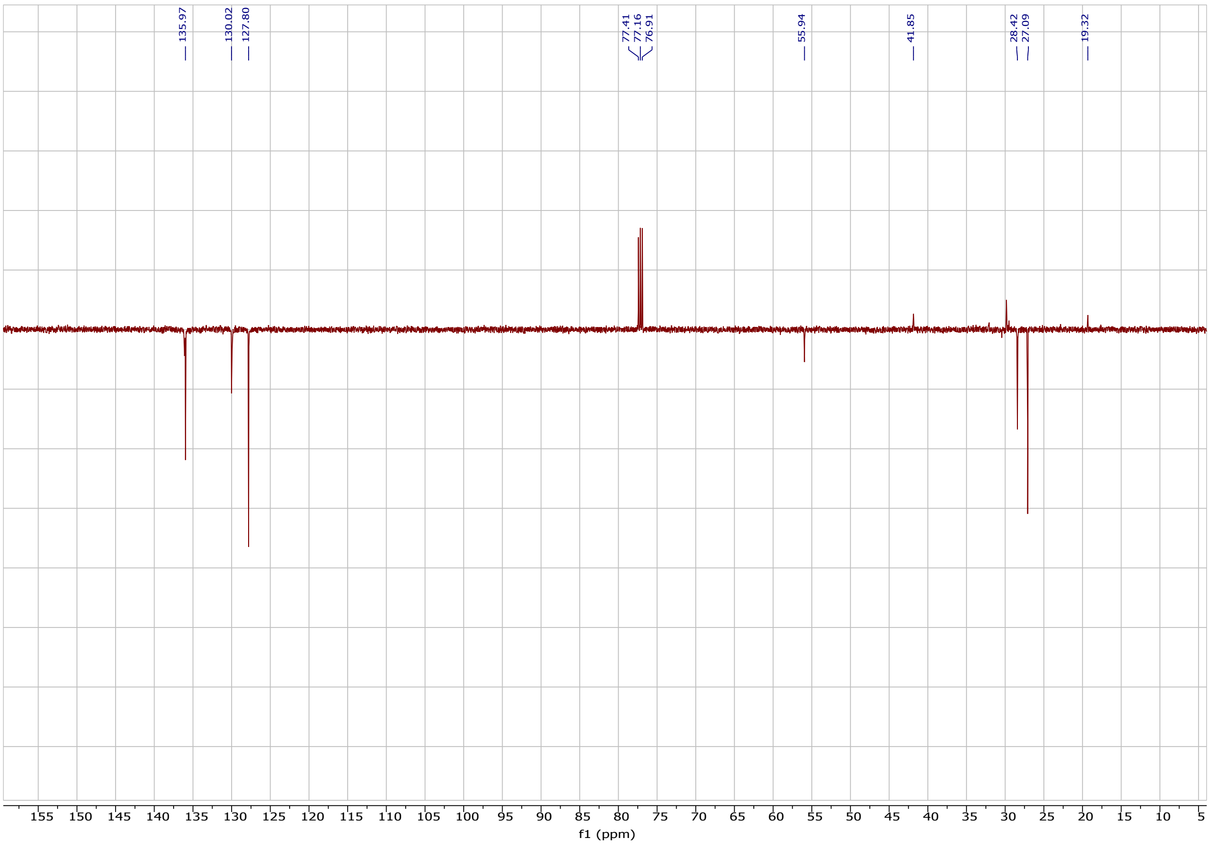

^{1}H and ^{13}C of **11** in D_2O at 293 K

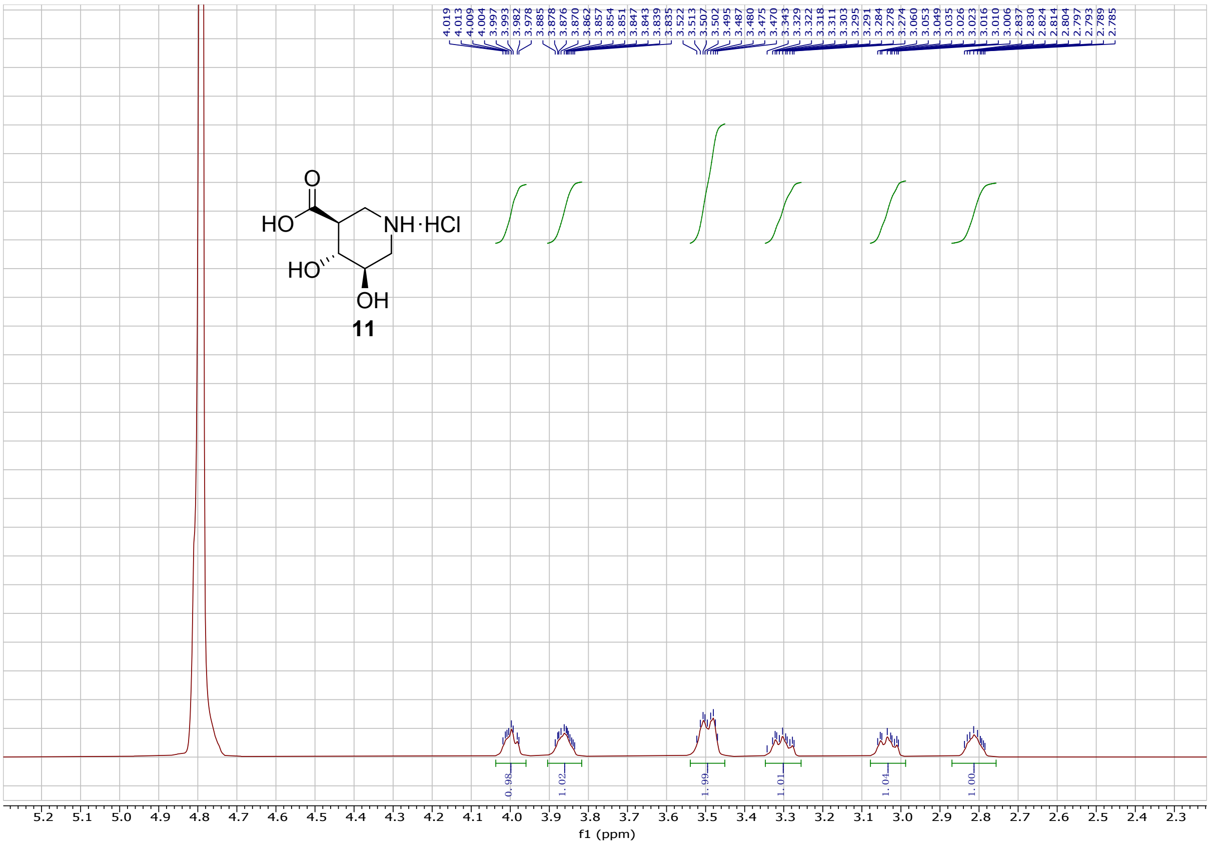

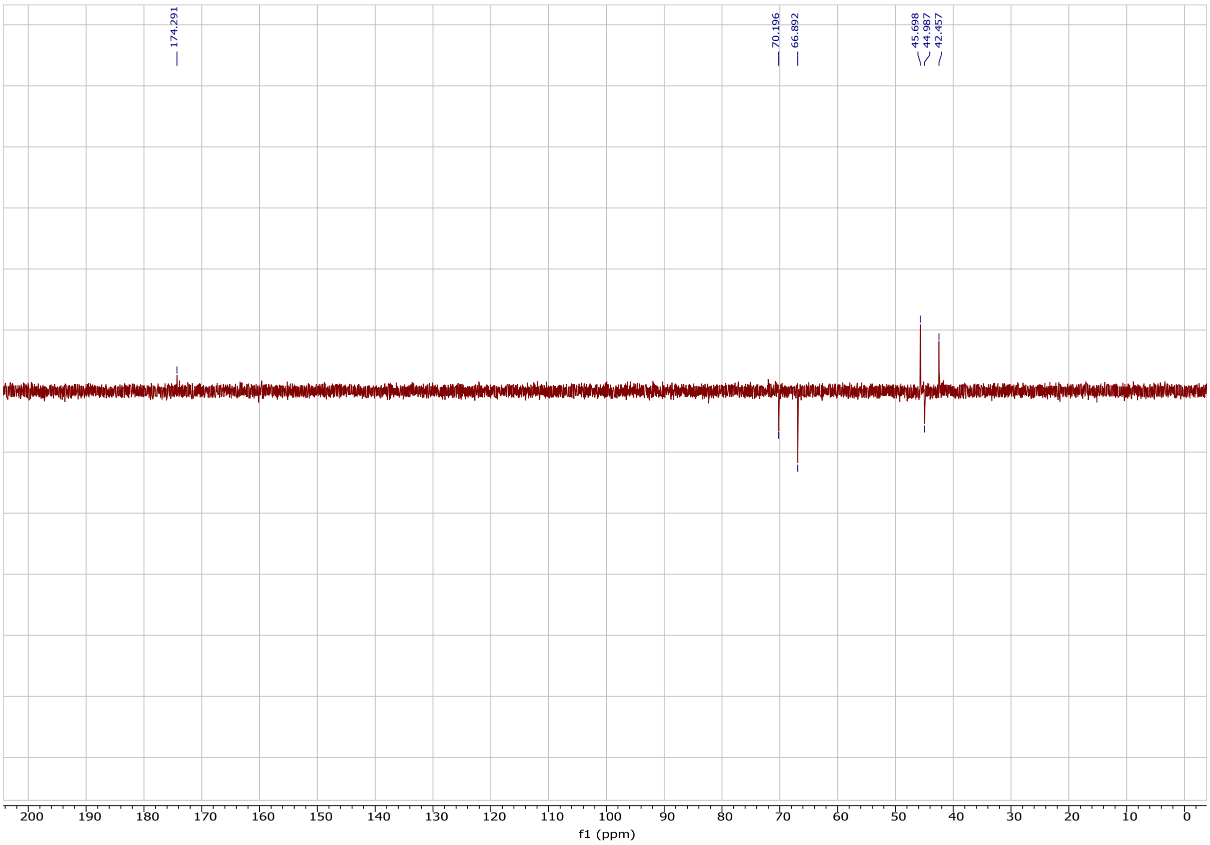

^1H and ^{13}C of **23** in $CDCl_3$ at 293 K

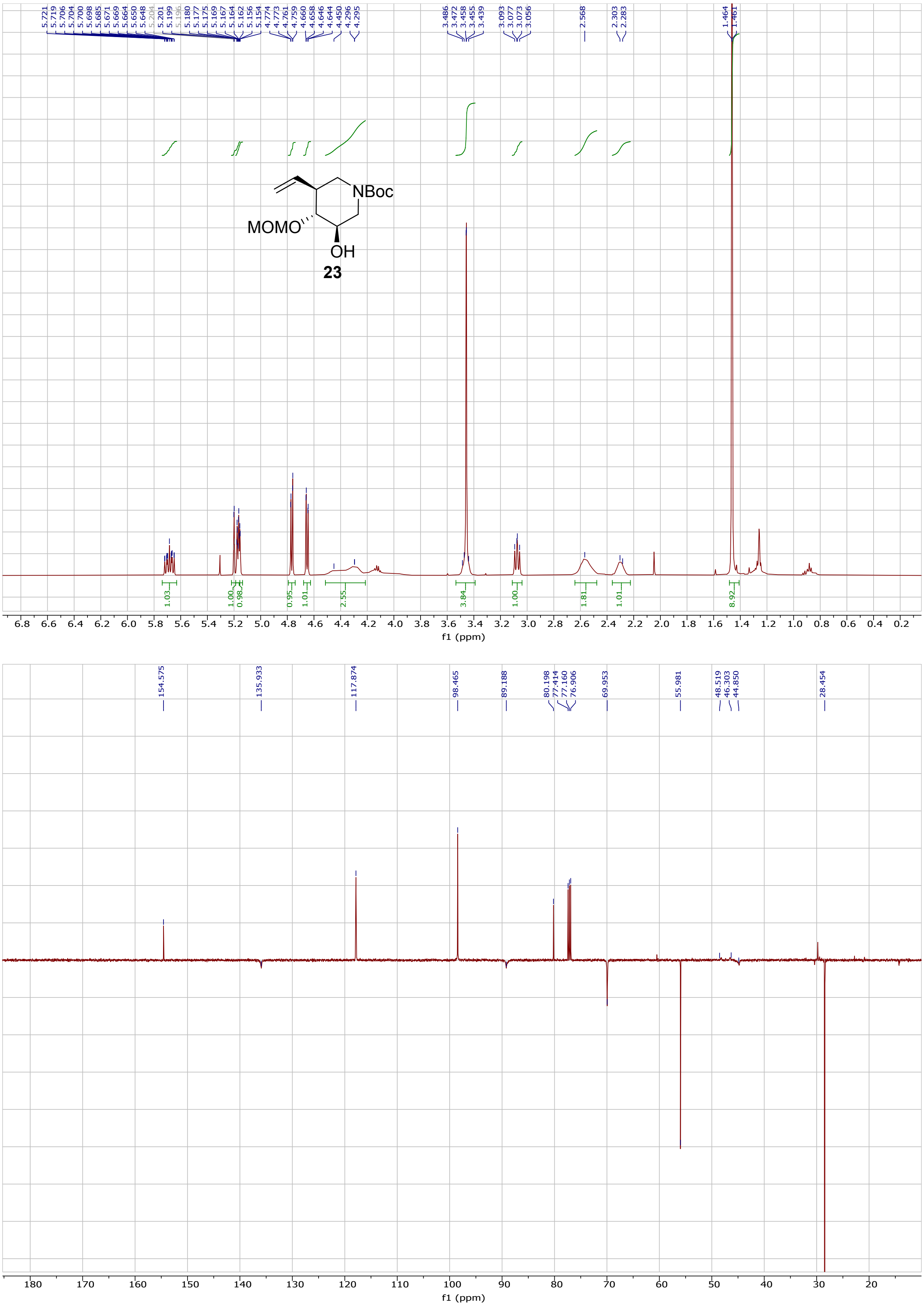

^{1}H and ^{13}C of **24** in $CDCl_3$ at 293 K

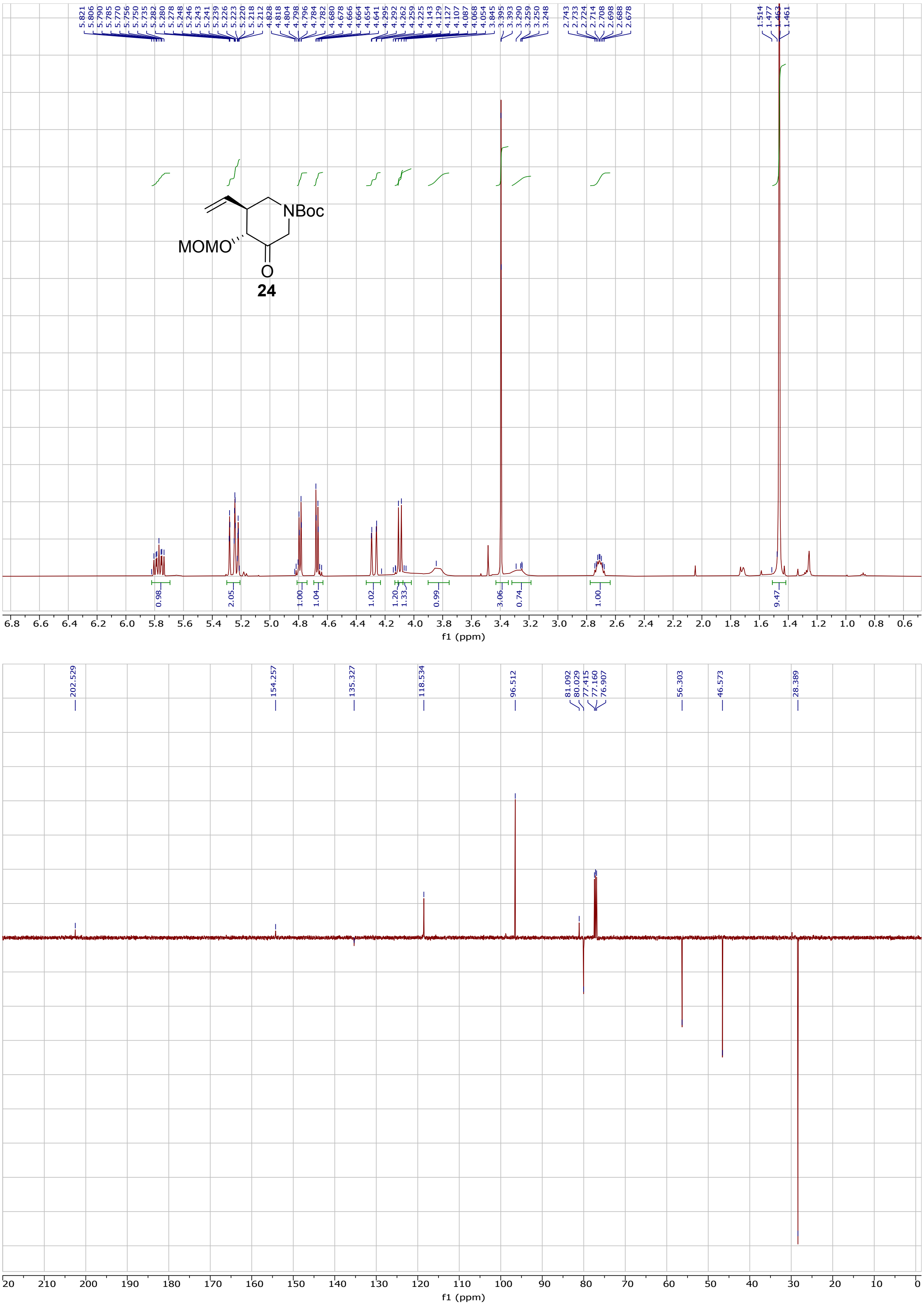

^{1}H and ^{13}C of **25** in $CDCl_3$ at 333 K

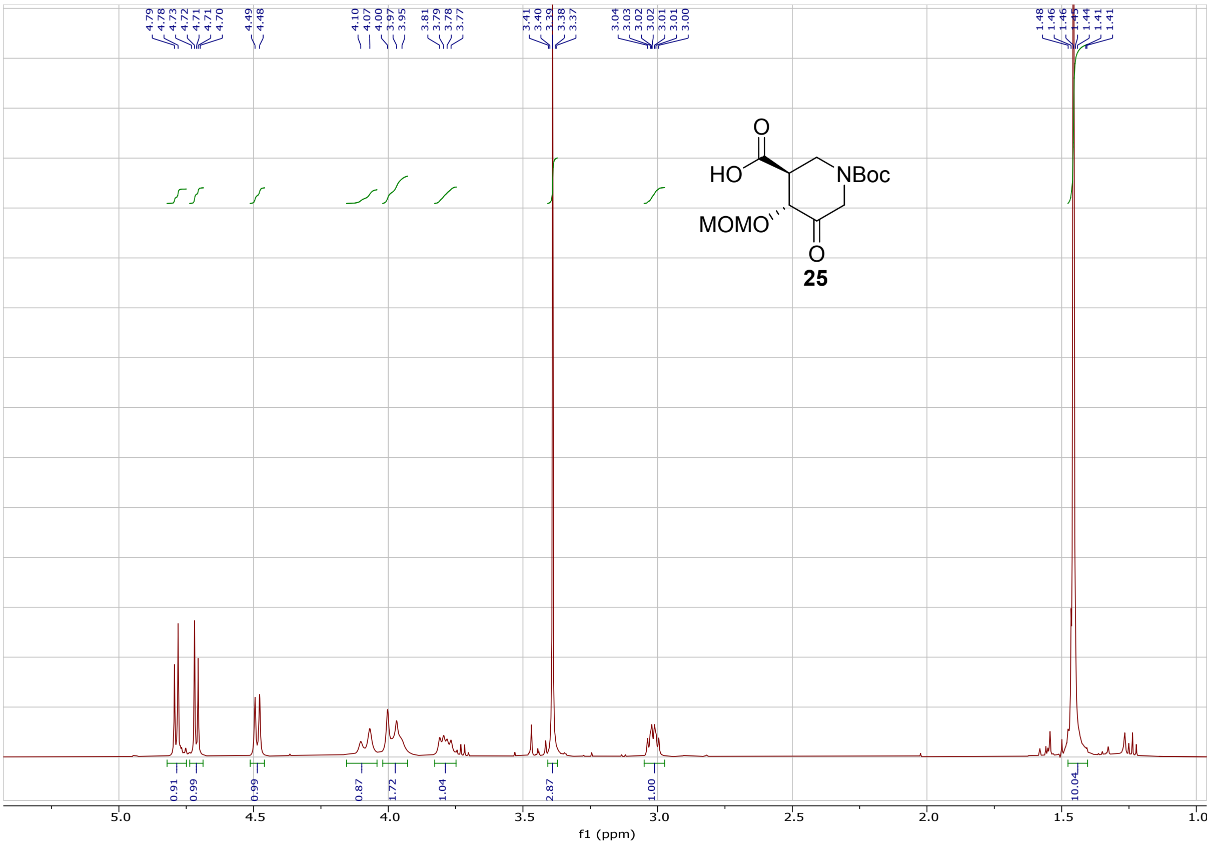

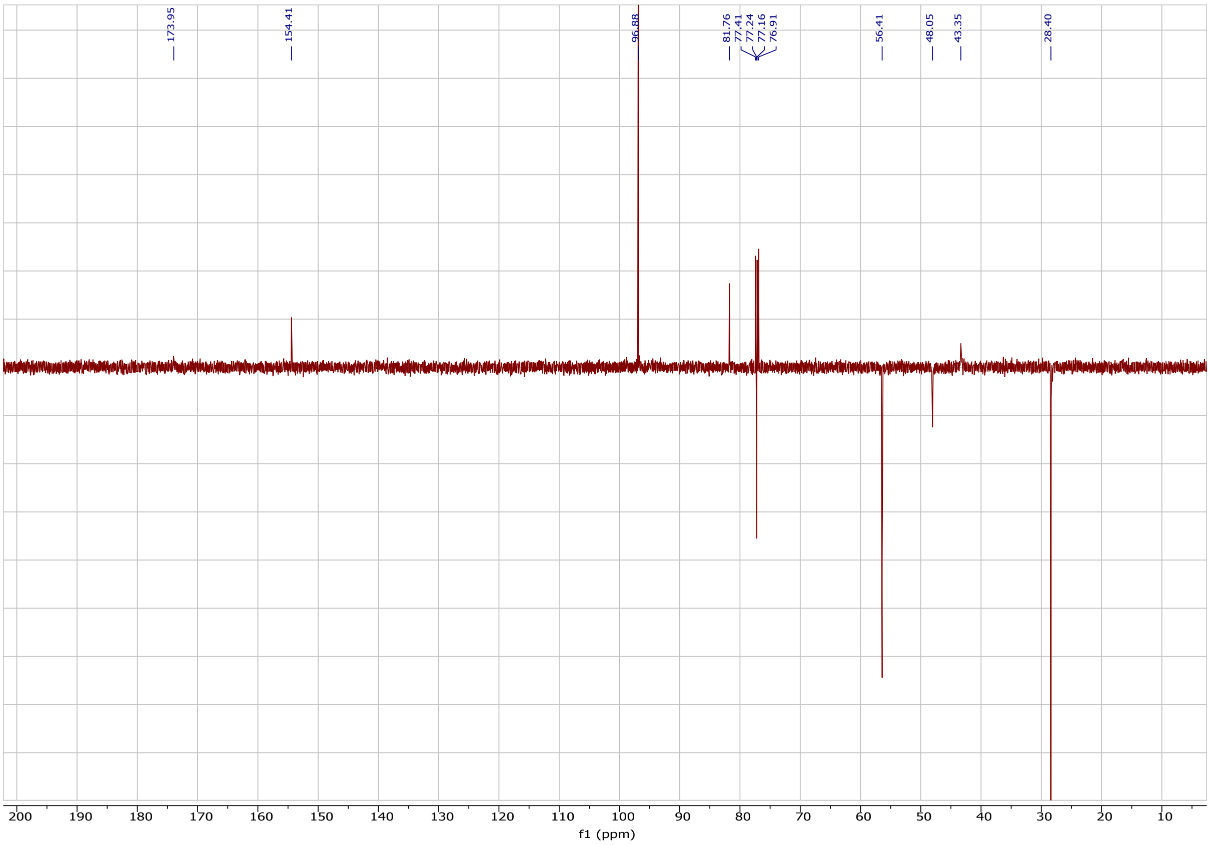

^{1}H and ^{13}C of **9** in D_2O at 293 K

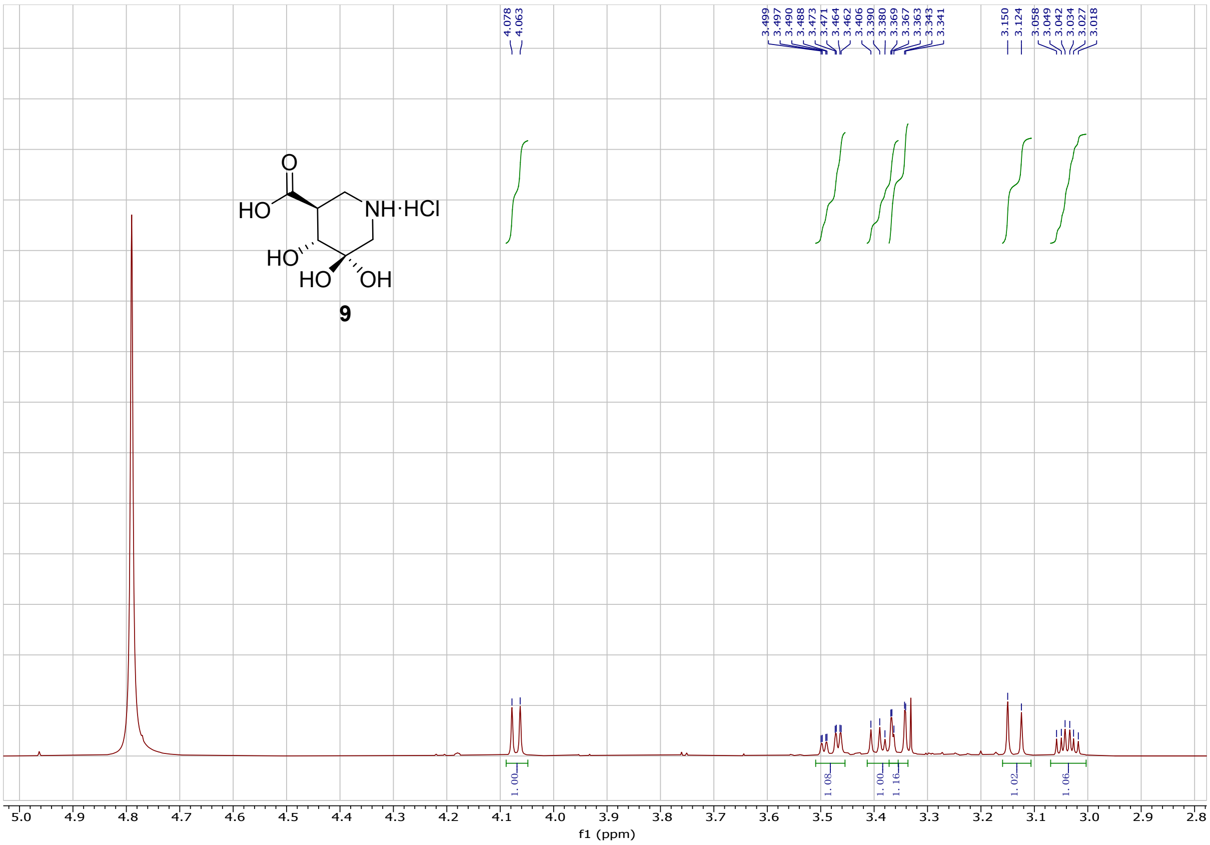

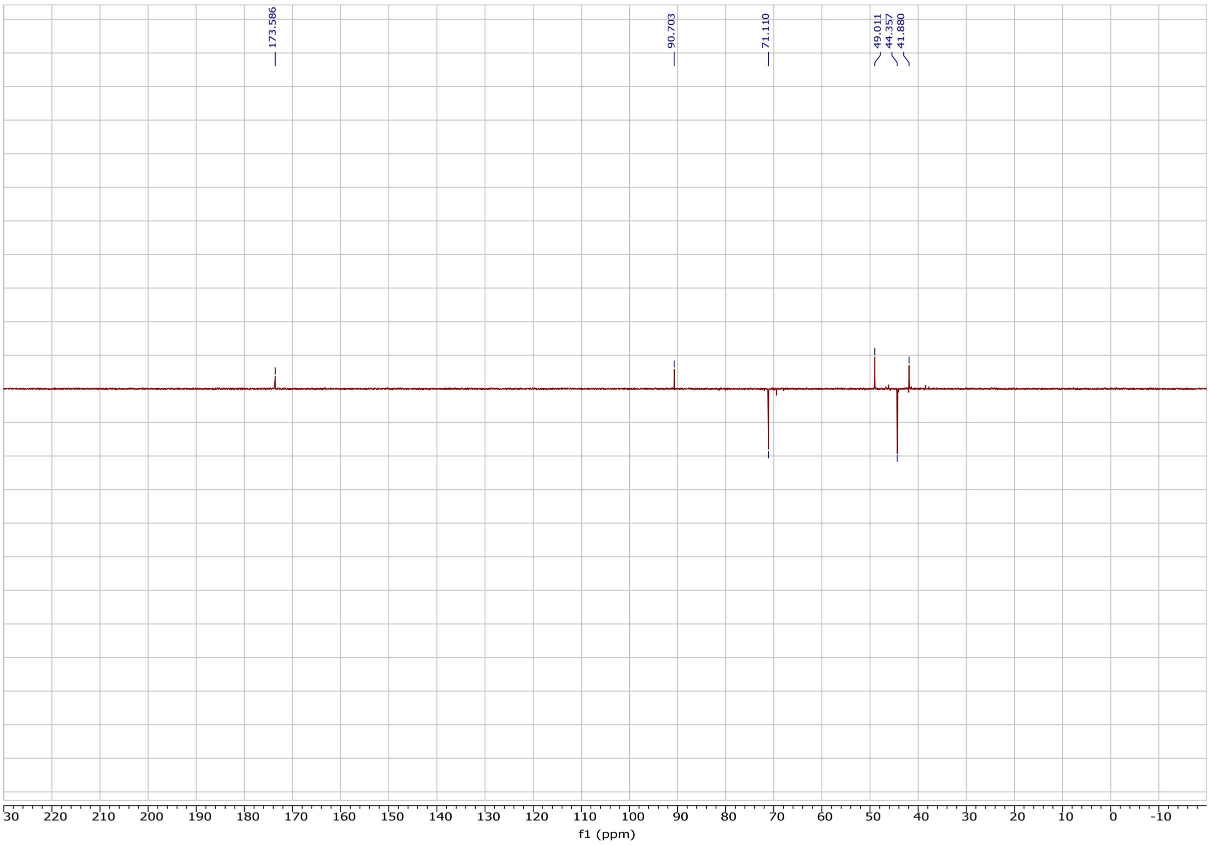

Supplement: Supplementary file 1 — ja3c04162_si_001.pdf [file ja3c04162_si_001.pdf]
